# A Modular Engineered DNA Nanodevice for Precise Profiling of Telomerase RNA Location and Activity

**DOI:** 10.1002/advs.202409344

**Published:** 2024-12-27

**Authors:** Shi‐Yi Zhang, Jian Lv, Ze‐Rui Zhou, Peter X. Geng, Da‐Wei Li, Ruo‐Can Qian, Huangxian Ju

**Affiliations:** ^1^ Key Laboratory for Advanced Materials Feringa Nobel Prize Scientist Joint Research Center Joint International Laboratory for Precision Chemistry Frontiers Science Center for Materiobiology & Dynamic Chemistry School of Chemistry and Molecular Engineering East China University of Science and Technology Shanghai 200237 P. R. China; ^2^ Department of Biomedical Engineering College of Future Technology Peking University Beijing 100871 P. R. China; ^3^ State Key Laboratory of Analytical Chemistry for Life Science School of Chemistry and Chemical Engineering Nanjing University Nanjing 210023 P. R. China

**Keywords:** DNA engineering, human telomerase RNA, telomerase activity

## Abstract

Increased telomerase activity has been considered as a conspicuous sign of human cancers. The catalytic cores of telomerase involve a reverse transcriptase and the human telomerase RNA (hTR). However, current detection of telomerase is largely limited to its activity at the tissue and single‐cell levels. To reveal the precise distribution of subcellular hTR and telomerase activity, here a modular engineered DNA nanodevice (DNA‐ND) is designed capable of imaging hTR and telomerase activity in cytoplasm and nucleus, enabling colocalization analysis. DNA‐ND is a modular DNA complex comprising hTR and telomerase activity detection modules, which respectively sense intercellular hTR and telomerase activity via target‐sensitive allosteric transition of DNA switches, actuating orthogonal activation of fluorescence signals to achieve in situ co‐imaging of hTR and telomerase activity. By integrating DNA‐ND with specific localized signals, the DNA‐ND based precise profiling of colocalization of hTR and telomerase activity in different cell lines as well as their dynamic changes during pharmacological interventions is demonstrated. Notably, the results suggest that the locations of hTR and telomerase activity are not exactly overlapped, indicating the influence of intracellular environment on the binding of hTR to telomerase.

## Graphical abstract

Telomerase is a key cancer biomarker vital for cellular processes. A modular DNA nanodevice is designed to profile hTR location and telomerase activity, enabled in situ co‐imaging in cytoplasm and nucleus. A logic imaging method distinguished colocalized from non‐colocalized subcellular regions, aiding statistical analysis of hTR/active telomerase colocalization in various cell lines, improving understanding of telomerase‐related pathologies and cancer activation.

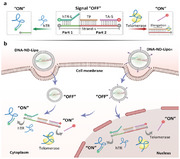

## 1 Introduction

Human telomeres are repetitive G‐rich hexameric sequences (TTAGGG) located at the end of chromosomes.^[^ ^1^ ^]^ Telomere homeostasis is fundamental to various cellular processes including cell senescence, chromosome replication, and tumor progression.^[^ ^2^ ^]^ In normal somatic cells, the length of telomeres decreases during each cycle of cell division, and this progressive shortening is the major timing mechanism to control the lifespan of cells.^[^ ^3^ ^]^ In contrast, in most tumor cell lines, the length of telomeres can be maintained upon the activation of telomerase, a reverse transcriptase that is able to add telomere repeats to the end of chromosomes.^[^ ^4^ ^]^ It is overexpressed in various cancer cells, and has been considered as a conspicuous sign of tumor.^[^ ^5^ ^]^ The catalytic core of human telomerase is composed of a catalytic protein subunit and the template RNA (human telomerase RNA, hTR).^[^ ^6^ ^]^ Since hTR is necessary for telomere elongation by telomerase, its expression has been used to reveal telomerase activity.^[^ ^7^ ^]^ However, several works have demonstrated that the expression of hTR is not in proportional to telomerase activity.^[^ ^8^ ^]^ Therefore, it is critically significant to design the detection tools for precise profiling of hTR location and telomerase activity, and understanding of telomerase‐related pathologies and telomerase activation in cancer. Up to now, a lot of probes and sensors have been developed for the detection of hTR or telomerase. Various probes with logic gate design and different carriers have been developed to achieve the physiological activity of telomerase in cells.^[^ ^9^ ^]^ However, detection of hTR is still rare and the simultaneous detection in live tumor cells can not be achieved.^[^ ^10^ ^]^ Therefore, it is still an urgent need to develop a strategy for in situ subcellular co‐imaging of hTR and telomerase activity in living cells.

Recently, DNA‐based fluorescent reporters have been widely applied for sensing and imaging of intracellular cancer biomarkers.^[^ ^11^ ^]^ The advantages of these reporters are their high sensitivity, real‐time imaging abilities,^[^ ^12^ ^]^ high programmable flexibility, and versatility.^[^ ^13^ ^]^ Benefiting from the convenient synthesis and modification with various dyes and quenchers, a number of DNA‐based fluorescent probes have been developed for biomarker imaging in living cells.^[^ ^14^ ^]^ For example, several cancer cell‐selective DNA‐based fluorescent reporters have been designed for targeted imaging and detection of biological molecules among the multiple interactions of cells,^[^ ^15^ ^]^ and some multiple fluorescent DNA‐based probes have also been designed for precise cell manipulation and imaging.^[^ ^16^ ^]^

Inspired by previous achievements, here we report the rational design of a modular engineered DNA nanodevice (DNA‐ND) that enables fluorescence co‐imaging of hTR and telomerase activity at the subcellular level. The DNA‐ND is target‐activatable and integrated with a specific subcellular site localized signal. The fluorescence signals of DNA‐ND can be orthogonally turned “on” via hTR‐sequence hybridization and telomerase‐induced strand elongation. A logic image processing method is used to distinguish colocalized and non‐colocalized regions of hTR and activated telomerase in cytoplasm or nucleus, which facilitates the statistical analysis of hTR/active telomerase colocalization in different cell lines. The dynamic change of hTR and telomerase activity during pharmacological interventions has demonstrated that BIBR1532 can lead to a fall of the telomerase activity, especially in cytoplasm, by triggering the combination inhibition between hTR and the C‐terminal extension (CTE) region of telomerase protein. In contrast, Sinefungin can upregulate the level of hTR, along with the enhanced telomerase activity in both cytoplasm and nucleus, by recruiting hTR in nucleus.

## 2 Results and Discussion

### 2.1 Design and Implementation of Modular Engineered DNA‐ND System

**Scheme 1:**
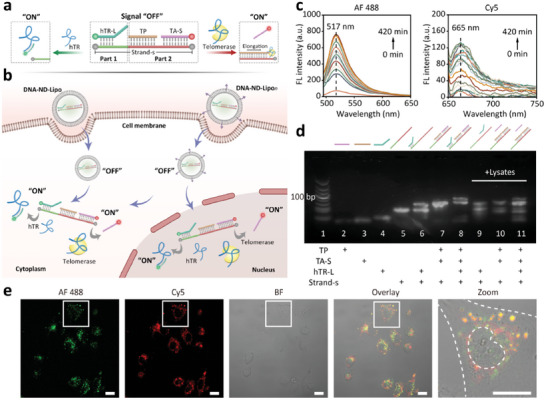
Working principle of the modular engineered DNA‐ND. a) Modular design of DNA‐ND for orthogonally turning “on” the fluorescence signals of hTR and active telomerase. b) In situ localization and fluorescence imaging of hTR and telomerase activity in cytoplasm and nucleus with DNA‐ND‐Lipo and DNA‐ND‐Lipo*_n_*. c) Fluorescence spectra of AF 488 and Cy5 of DNA‐ND in 1 × PBS solution treated by HeLa cell lysates for different times (from 0 to 420 min). d) Gel electrophoresis to verify the working principle of the DNA‐ND with HeLa lysates. Lane 1: DNA ladder. Lane 2: TP. Lane 3: TA‐S. Lane 4: hTR‐L. Lane 5: Strand‐S. Lane 6: hybridized strands formed by hTR‐L and Strand‐S. Lane 7: hybridized strands formed by TP, TA‐S, and Strand‐S. Lane 8: DNA‐ND. Lane 9–11: samples of hybridized strands corresponding to Lane 6–8 treated by HeLa cell lysates at 37 °C for 2 h. e) Confocal fluorescence images of HeLa cells incubated with DNA‐ND‐Lipo/DNA‐ND‐Lipo*_n_* for 2 h. AF 488: green fluorescence. Cy5: red fluorescence. BF: bight‐field. Overlay: mixed fluorescence & bright‐field channel. Zoom: enlarged image of single cell of mixed florescence & bright‐field channel. Scale bar: 20 µm.

DNA‐ND contains two modular parts (parts 1 and 2) (**Scheme** **1**a), which are composed via the hybridization of a substrate strand and three functional strands (Table S1 and Figures S1 and S2, Supporting Information). Part 1 is the hTR detection module, as the 3′‐end of the substrate strand (Strand‐S) is partially hybridized with a hTR‐targeting ligand strand (hTR‐L). The 3′‐end of hTR‐L is designed to be the sticky end for hTR recognition. The 5′‐end of hTR‐L is labeled with a fluorophore, Alexa Fluor 488 (AF 488), which is quenched by BHQ1 via fluorescence resonance energy transfer (FRET).^[^ ^17^ ^]^ In the presence of hTR, the sticky end of hTR‐L binds with hTR to induce the disruption of the FRET pair, thus the fluorescence signal (AF 488) corresponding to hTR can be turned “on”. Part 2 is the detection module for telomerase activity. It contains two functional strands hybridized with Strand‐S: a telomerase primer strand (TP) and a telomerase activated signal strand (TA‐S). TA‐S is labeled with Cy5 at 3′‐end, which is quenched by BHQ3 tagged at the 5′‐end of Strand‐S. The TP sequence can be elongated at the 3′‐end to produce a telomeric repeated sequence complementary to the TA‐S hybridized part in Strand‐S, which leads to substitutional hybridization to remove the TA‐S. This process turns on the fluorescence of Cy5 as a result of the departure of Cy5 from BHQ3, thus lighting up the activated telomerase. After encapsulating DNA‐ND in a liposome, a typical nuclear localized sequence (NLS), CGYGPKKKRKVGG,^[^ ^18^ ^]^ is conjugated on the surface of liposome (DNA‐ND‐Lipo*_n_*) to deliver DNA‐ND into nucleus. The unmodified liposome is used to deliver DNA‐ND into cytoplasm (DNA‐ND‐Lipo). Upon incubation with DNA‐ND‐Lipo/DNA‐ND‐Lipo*_n_*, subcellular translocation of DNA‐ND can be achieved, which enables subcellular localization of hTR and telomerase activity in living cells (Scheme 1b).

The feasibility of DNA‐ND was first assessed in PBS solution (1 ×, pH = 7.2) by fluorescence spectra. After adding 100 µL HeLa cell lysates in DNA‐ND dispersion, the fluorescence intensity of AF 488 (hTR) and Cy5 (telomerase activity) significantly enhanced in a time‐dependent manner (Scheme 1c), indicating that DNA‐ND responded rapidly to cancer cell lysates containing hTR and active telomerase. The structure change of DNA‐ND was validated by gel electrophoresis (Table S2 and Figure S3, Supporting Information). As illustrated in Scheme 1d, Lanes 7, and 8 represented hTR and telomerase activity recognition modules, respectively. Upon the treatment of HeLa cell lysates (2 h), a short band in lane 11 corresponding to TA‐S emerged, suggesting that the telomerase‐triggered elongation of TP induced the departure of TA‐S. The longer band corresponded to the hybridized structure of TP, TA‐S, and Stand‐s (lanes 10, 11). The sticky end of hTR‐L could be specifically recognized by hTR, resulting in the hybridization between hTR‐L and hTR to trigger the detachment of hTR‐L from DNA‐ND (lanes 9, 11). Therefore, the hybridized hTR‐L and Strand‐S could be unwound to generate a shorter band corresponding to Strand‐S upon the treatment of cell lysates. Control experiments using heat‐inactivated telomerase and unrelated RNA showed negligible fluorescence before and after incubation (Figures S4 and S5, Supporting Information), which confirmed the targeted response of DNA‐ND to activated telomerase and hTR. A negative control experiment using mismatched DNA strands did not show the fluorescence recovery, indicating a specific response of DNA‐ND to hTR and telomerase (Figures S6–S8, Supporting Information). The absence of new band in gel electrophoresis demonstrated the failure of recognition between mismatched DNA‐ND and hTR, verifying the specific binding mechanism (Figures S9 and S10, Supporting Information and DNA sequences were listed in Table S3, Supporting Information). After DNA‐ND was mixed with cell lysates corresponding to different amounts of HeLa cells, and the fluorescence intensity of AF 488 (hTR) and Cy5 (telomerase activity) increased with the cell amount (Figure S11 and S12, Supporting Information), which demonstrated the positive correlation between the fluorescence and the level of hTR and telomerase in cell lysates. The fluorescence signal of DNA‐ND was stable after 24 h in different solutions, which indicated the fluorescence reliability of DNA‐ND (Figure S13, Supporting Information).

To construct the nanodevice and improve its biocompatibility, liposomes were used to encapsulate the DNA‐ND (Figure S14, Supporting Information), which facilitated the transmembrane delivery (Figures S15 and S16, Supporting Information).^[^ ^19^ ^]^ The size of DNA‐ND‐Lipo became larger comparing to bare liposome (Figure S17, Supporting Information). The particle size of DNA‐ND‐Lipo*_n_* increased further when NLS was modified. The ability of the mixture of DNA‐ND‐Lipo and DNA‐ND‐Lipo*_n_* (1: 1 by volume) to image intracellular hTR and telomerase activity in HeLa cells was tested with confocal laser scanning microscopy (CLSM). After HeLa cells (1 mL, 5.0 × 10^4^ mL^−1^) were treated with DNA‐ND‐Lipo/DNA‐ND‐Lipo*_n_* (220 µL, 300 nm) for initial 30 min, no obvious fluorescence signal was observed. After 60 min, the fluorescence of AF 488 (hTR) and Cy5 (telomerase activity) occurred in the cytoplasm and nucleus, and the fluorescence intensity gradually increased with the increasing incubation time, which reached the maximum value at 120 min (Figure S18, Supporting Information). Similarly, the concentration of DNA‐ND‐Lipo/DNA‐ND‐Lipo*_n_* was optimized to be 300 nm (Figure S19 and Table S4, Supporting Information). As shown in Scheme 1e, bright fluorescence could be observed in the confocal images of HeLa cells incubated with DNA‐ND‐Lipo/DNA‐ND‐Lipo*_n_* under optimal conditions. In contrast, the control experiments using mismatched DNA strands showed negligible fluorescence in HeLa cells (Figures S20 and S21, Supporting Information). Moreover, cell viability assays showed negligible cytotoxicity of DNA‐ND‐Lipo/DNA‐ND‐Lipo*_n_* (Figures S22–S25, Supporting Information). These results validated the design of DNA‐ND, which enabled fluorescence imaging of subcellular hTR and telomerase activity.

### 2.2 Using DNA‐ND to Show Subcellular Profiles of hTR and Telomerase Activity in Different Cell Lines

**Figure 1:**
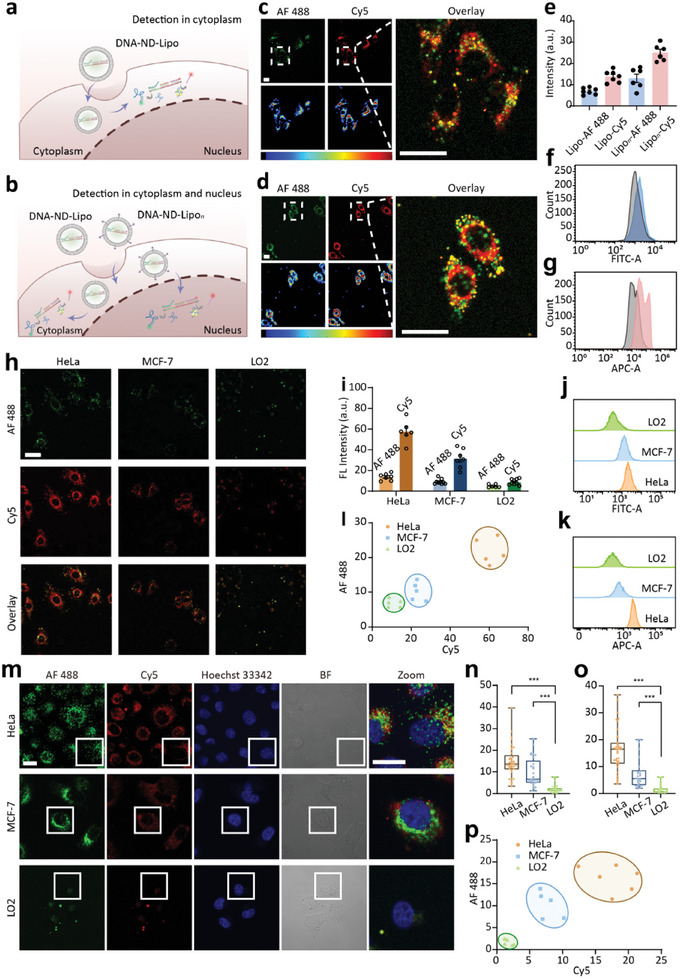
Detection of hTR and telomerase activity in different cell lines. a) In situ imaging of hTR and telomerase activity in cytoplasm. b) In situ imaging of hTR and telomerase activity in cytoplasm and nucleus. c,d) Confocal fluorescence images of HeLa cells incubated with DNA‐ND‐Lipo and DNA‐ND‐Lipo/DNA‐ND‐Lipo*_n_* for 2 h. The fluorescence intensity of different channels was shown by pseudocolor images. Color bar: 10 to 150 from left to right. e) The fluorescence intensity corresponding to confocal fluorescence images in (c) and (d). f,g) Flow cytometric analysis of HeLa cells treated with DNA‐ND‐Lipo and DNA‐ND‐Lipo/DNA‐ND‐Lipo*_n_*. h) Confocal fluorescence images of different cells incubated with DNA‐ND‐Lipo/DNA‐ND‐Lipo*_n_*. i) The fluorescence intensity corresponding to confocal fluorescence images in (h). j) k) Flow cytometric analysis of different cells treated with DNA‐ND‐Lipo/DNA‐ND‐Lipo*_n_*. l) Scatter plot showing fluorescence distribution of AF 488 versus Cy5 corresponding to cells in (h). m) Confocal images showing the fluorescence of AF 488 and Cy5 in nucleus region of different cells. n,o) The fluorescence intensity of AF 488 and Cy5 in nucleus region corresponding to cells in (m). The significance of the mean fluorescence intensity was assessed by *t*‐test. Each data point represents one cell. (*n* = 30, ^***^ *p* < 0.001). (p) Fluorescence distribution of AF 488 and Cy5 corresponding to cells in (m). Each dot corresponded to the average fluorescence intensity of 5 cells. AF 488: green fluorescence. Cy5: red fluorescence. Overlay: mixed fluorescence channel. Zoom: enlarged images of a single cell of mixed fluorescence channel. Scale bar: 20 µm.

We next explored the feasibility of DNA‐ND for the imaging of hTR and telomerase activity in different cell lines (**Figure** **1**). DNA‐ND‐Lipo and the mixture of DNA‐ND‐Lipo/DNA‐ND‐Lipo*_n_* (1: 1 by volume) were used for the delivery of DNA‐ND respectively (Figure 1a,b). We first checked the fluorescent distribution of AF 488 (hTR) and Cy5 (telomerase activity) in HeLa cells (Figure 1c,d). By comparing the fluorescent distribution in merge and pseudocolor images, the fluorescence areas of AF 488 (hTR) and Cy5 (telomerase activity) were not exactly overlapped, especially in the nucleus (Figure 1d). Notably, compared with HeLa cells treated with only DNA‐ND‐Lipo, the average fluorescence intensity was significantly higher in HeLa cells treated with DNA‐ND‐Lipo/DNA‐ND‐Lipo*_n_* (Figure 1e), which was also validated by flow cytometric analysis (Figure 1f,g). These results demonstrated the nuclear‐targeting ability of DNA‐ND‐Lipo*_n_*. Therefore, DNA‐ND‐Lipo/DNA‐ND‐Lipo*_n_* mixture (1: 1 by volume) was used for all the following cell imaging experiments to show the subcellular profiles of hTR and telomerase activity in both cytoplasm and nucleus.

According to previous reports,^[^ ^20^ ^]^ the expression levels of hTR and telomerase activity vary across different cell lines. We evaluated the performance of DNA‐ND in three different cell lines, including HeLa cells, MCF‐7 cells, and LO2 normal cells. The overall fluorescence intensity of AF 488 (hTR) in HeLa cells was higher than that in MCF‐7 cells, while LO2 normal cells expressed the lowest average fluorescence intensity (Figure 1h). The same trend was observed in the overall fluorescence intensity of Cy5 (telomerase activity). Corresponding fluorescence histograms and flow cytometric analysis confirmed these results (Figure 1i–k). Moreover, the scatter plot showed the fluorescence distribution of AF 488 (hTR) versus Cy5 (telomerase activity), from which different cells could be well distinguished (Figure 1l). Specifically, hTR was inclined to reflect the level of mature telomerase complex during its post‐transcriptional maturation,^[^ ^21^ ^]^ thus hTR could be used to indicate the upper limit of telomerase quantity content, rather than an indicator of telomerase activity.^[^ ^22^ ^]^

Next, we sought to investigate the distribution of hTR and telomerase activity in nuclear areas of HeLa cells, MCF‐7 cells, and LO2 normal cells (Figure 1m). From confocal cell images showing the nuclear areas, it could be seen that the fluorescence of AF 488 and Cy5 was dispersed throughout the nucleus in both cancer cells. The fluorescence intensity of AF 488 (hTR) was the highest in the nucleus of HeLa cells, while negligible fluorescence was observed in the nucleus of LO2 normal cells (Figure 1n,o). The fluorescence of Cy5 (telomerase activity) exhibited a similar tendency. These data revealed that only a small fraction of the telomerase protein‐hTR chimera was located in the nucleus of normal cells, but the proportion was upregulated in cancer cells.^[^ ^21^ ^]^ In addition, the scatter plot showing the fluorescence distribution of AF 488 (hTR) versus Cy5 (telomerase activity) in the nucleus could effectively distinguish different cells (Figure 1p). All these results validated the feasibility of DNA‐ND as an imaging nanosensor to achieve in situ and orthogonal co‐imaging of hTR and telomerase activity.

### 2.3 Interpretation of Confocal Fluorescence Images Obtained by DNA‐ND by Logic Processing

In order to interpret the subcellular fluorescence distributions observed in different cells, a logical operation based on MATLAB was employed to illustrate quantitatively the colocalization of AF 488 (corresponding to hTR) and Cy5 (corresponding to telomerase activity) by analyzing the mono‐channel fluorescence images with MATLAB code (Supporting Information). Depending on the fluorescence intensity, each pixel on the confocal fluorescence images was extracted to determine the presence (defined as 1) or absence (defined as 0) of fluorescence signal. Next, these binary values were transferred into two matrices corresponding to the green (AF 488) and red (Cy5) fluorescence channels, respectively. By applying a logical “XOR” operation, pixels with a value of 1 were endowed with white color to generate grayscale images (Figure S26, Supporting Information). Specifically, the existence of either green or red fluorescence could generate an output value of 1. This pixel‐based conversion significantly enhanced the visibility and clarity of non‐colocalized regions in cell images. Similarly, a logical “AND” operation was used to enhance the colocalized regions where both green and red fluorescence were presented. By constructing the “XOR” and “AND” logical grayscale images, the non‐colocalized and colocalized areas of AF 488 (hTR) and Cy5 (telomerase activity) could be visually distinguished, and the area corresponding to each channel could be quantified to show the subcellular distribution profiles of hTR and telomerase activity. Moreover, the colocalized pixels could be used to calculate the ratio of colocalized areas in different subcellular regions by the following formula:

(1) $$\begin{array}{ccc} & & Colocalized\;percent\left(\%\right)\\ & & =\frac{Average\;colocalized\;pixels\;in\;nucleus\left(orcytoplasm\right)}{Average\;pixels\;counted\;in\;nucleus\left(orcytoplasm\right)} \end{array}$$

**Figure 2:**
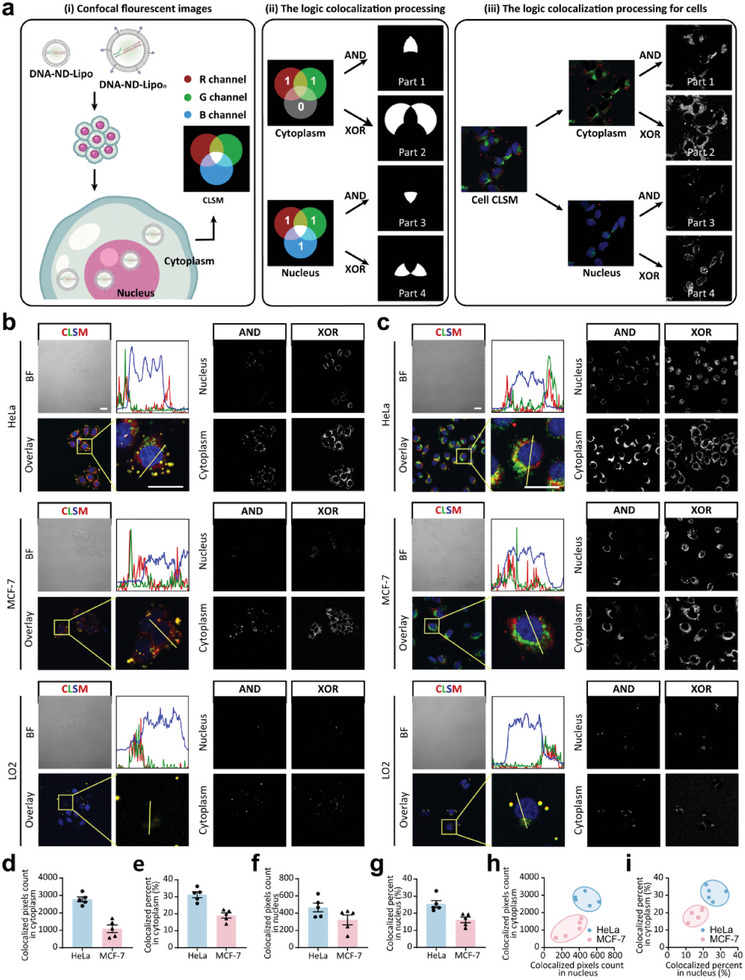
Logic processing of confocal fluorescence images. a) Schematic illustration of the operational mechanism of logic processing. Cells were treated with Hoechst 33342 to highlight the nuclear region. Gray‐scale images showing the co‐localization and non‐co‐localization regions in the nucleus and cytoplasm were extracted from the confocal fluorescence images by logic processing. b,c) Confocal fluorescence images and logic output images of HeLa cells, MCF‐7 cells, and LO2 normal cells treated with DNA‐ND‐Lipo and DNA‐ND‐Lipo/DNA‐ND‐Lipo*_n_*. AF 488: green fluorescence. Cy5: red fluorescence. Hoechst 33342: blue fluorescence. BF: bight‐field. Overlay: mixed fluorescence channel. Scale bar: 20 µm. d) Colocalized pixels were counted in the cytoplasm of HeLa cells and MCF‐7 cells treated with DNA‐ND‐Lipo/DNA‐ND‐Lipo*_n_*. e) Proportion of colocalized region in cytoplasm according to statistical data in (d). f) Colocalized pixels counted in the nucleus of HeLa cells and MCF‐7 cells treated with DNA‐ND‐Lipo/DNA‐ND‐Lipo*_n_*. g) Colocalized pixels are counted in the nucleus according to statistical data in (f). h) Colocalization distribution of pixels in the nucleus and cytoplasm according to statistical data in (d) and (f). i) Colocalization distribution of colocalized percent in the nucleus and cytoplasm according to statistical data in (e) and (g).

Based on these logic operations, the fluorescent distributions in different cell lines could be clearly distinguished. Upon the processing of previous confocal fluorescence images, (Figure 1m) of the cells incubated with DNA‐ND‐Lipo/DNA‐ND‐Lipo*_n_*, the green and red channels were not exactly overlapped, especially in the nucleus. Thus, we could divide the cell area with fluorescence signal into four different parts (**Figure** **2**a): colocalized area in cytoplasm (part 1), non‐colocalized area in cytoplasm (part 2), colocalized area in the nucleus (part 3) and non‐colocalized area in the nucleus (part 4). Corresponding grayscale images of these parts of HeLa and MCF‐7 cancer cells are shown in Figure 2b,c. Colocalized pixels and the proportion of colocalized region in cytoplasm and nucleus were shown in Figure 2d–g. From the above results, we observed that the colocalized area in HeLa cells was significantly higher than that in MCF‐7 cells, the proportion of colocalization within the cytoplasm in HeLa cells was 1.5 times of that in MCF‐7 cells. Notably, the proportion of colocalization within the nucleus in MCF‐7 cells was obviously lower than that in HeLa cells, suggesting a lower binding rate between telomerase protein and hTR. In addition, different cancer cells (HeLa cells and MCF‐7 cells) could be easily discriminated by the scatter plots showing the colocalization distribution (Figure 2h,i). Our results proved that the logic processing could distinguish subtle differences in the proportion of subcellular expression between hTR and telomerase activity, and could help us understand underlying factors behind the differences in tumor cell proliferation.

### 2.4 Profiling Distribution Changes of hTR and Telomerase Activity Under Drug Treatment by DNA‐ND

**Figure 3:**
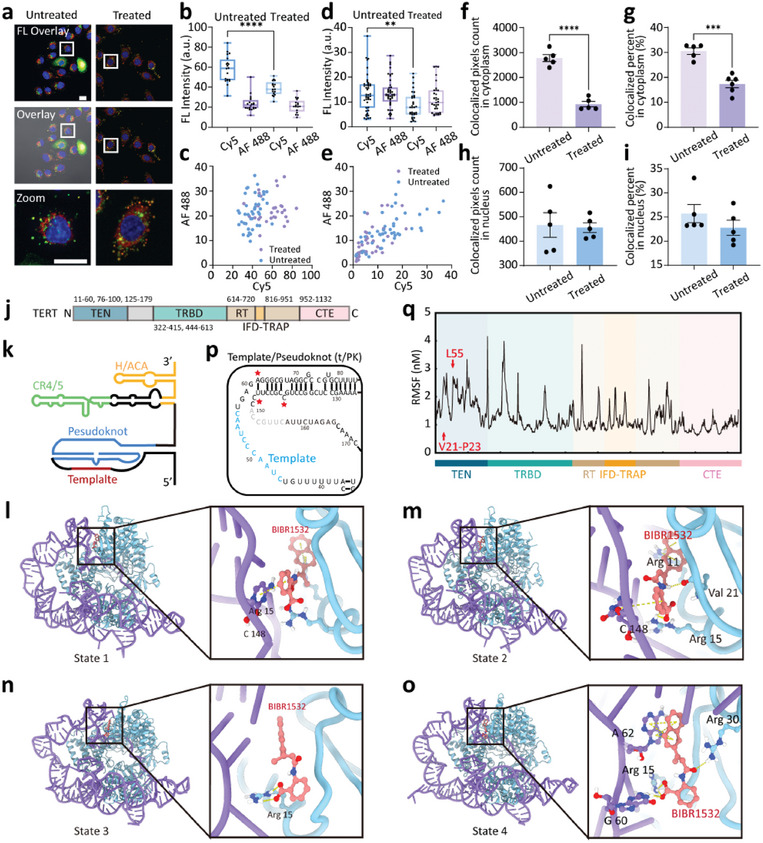
Expression of hTR and telomerase activity upon BIBR1532 treatment. a) Confocal fluorescence images of HeLa cells incubated with DNA‐ND‐Lipo/DNA‐ND‐Lipo*_n_* and without or with BIBR1532 treatment. FL Overlay: mixed fluorescence channel. Overlay: mixed fluorescence & bright‐field channel. Zoom: enlarged image of a single cell of mixed fluorescence. Scale bar: 20 µm. b) Fluorescent intensity of hTR (AF 488) and telomerase activity (Cy5) in cytoplasm region corresponding to cell images in (a). Each point represents one cell (*n* = 20). c) Fluorescence distribution of hTR (AF 488) and telomerase activity (Cy5) in cytoplasm corresponding to cells without or with BIBR1532 treatment. d) Fluorescent intensity of hTR (AF 488) and telomerase activity (Cy5) in the nucleus region corresponding to cell images in (a). Each data point represents one cell (*n* = 30). e) Fluorescence distribution of hTR (AF 488) and telomerase activity (Cy5) in the nucleus corresponding to cells without or with BIBR1532 treatment. f) Colocalized pixels counted in cytoplasm of HeLa cells without or with BIBR1532 treatment. Each dot represents the average of 5–10 cells (*n* = 5). g) Proportion of colocalized region in cytoplasm according to statistical data in (f). Each dot represents the average of 5–10 cells (*n* = 5). h) Colocalized pixels counted in the nucleus of HeLa cells without or with BIBR1532 treatment. i) Colocalized pixels counted in the nucleus according to statistical data in (h). Each dot corresponded to the average of 10–20 cells. j) Domain architecture of TERT. k) Secondary structure of hTR. Regions invisible in the cryo‐EM map are shown in grey. l–o) The telomerase‐specific inhibitor BIBR1532 was docked onto the TERT TEN‐BIBR1532 structure (PDB: T7RD) with four states. Residues comprising for BIBR1532 binding were labeled in the zoomed regions. (p) Schematic illustration of the template/pseudoknot in hTR secondary structure. (q) RMSFs of TERT in the presence of BIBR1532. ^**^ *p* < 0.01, ^***^ *p* < 0.001, ^****^ *p* < 0.0001.

We proceeded to explore the cooperative expression of hTR and telomerase activity upon drug treatment in HeLa cells using the DNA‐ND based dual imaging protocol. We first tested BIBR1532 (2‐[(E)‐3‐naphtalen‐2‐yl‐but‐2‐enoylamino]‐benzoic acid), a synthetic non‐nucleoside compound that served as a specific inhibitor of TERT53. BIBR1532 can inhibit the catalytic activity of telomerase via binding with a non‐catalytic site at the telomerase core.^[^ ^23^ ^]^ CCK‐8 assay of HeLa cells treated by BIBR1532 showed negligible apoptosis (Figure S27, Supporting Information). The confocal fluorescence images of HeLa cells before and after BIBR1532 treatment showed the decrease in fluorescence area of Cy5 in both the nucleus and cytoplasm upon BIBR1532 treatment (**Figure** **3**a), indicating decreased telomerase activity. It was worth noting that the fluorescence intensity of AF 488 (hTR) was almost unchanged (Figure 3b), suggesting the constant level of hTR. Moreover, the cells without or with BIBR1532 treatment could be easily discriminated by the fluorescence distribution of AF 488 (hTR) and Cy5 (telomerase activity) in cytoplasm (Figure 3c). Notably, compared with the situation in cytoplasm, the fluorescence intensity in the nucleus did not show obvious change before and after BIBR1532 treatment (Figure 3d,e). Next, we checked the colocalized area in cytoplasm and nucleus of cells with or without BIBR1532 treatment by using logic processing (Figure 3f–I; Figure S28, Supporting Information), which was in accordance with previous results. Thus, the telomerase in cytoplasm was more accessible to BIBR1532, and the main location where the BIBR1532 drug works was the cytoplasm.

We further exploited the BIBR1532 based inhibition mechanism of telomerase activity by molecular simulation. As shown in the Cryo‐electron microscopy (cryo‐EM) image, the catalytic core of telomerase consisted of telomerase reverse transcriptase (TERT), hTR template‐pseudoknot (t/PK), and conserved regions 4–5 (CR4/5). TERT contained an RNA‐binding domain (RBD), a reverse transcriptase (RT), and a C‐terminal extension (CTE), which formed a ring‐like structure. The N‐terminal domain (TEN) and the telomerase RAP motif (TRAP) of TERT could affect telomerase activity (Figure 3j,k).^[^ ^24^ ^]^ Considering the above characteristics of TERT, a docking simulation between BIBR1532 and TERT was performed (Figure S29, Supporting Information). The simulation results (Figure 3l–o; Figures S30–S33, Supporting Information) showed that BIBR1532 could bind to the active pocket of the TERT, forming two π cation bonds with the protein amino acid residue ARG11, a hydrogen bond and a salt bridge with ARG15, and a *π–π* bond with the 148th C base of hTR in State 1. At 0 ns, BIBR1532 was bound in the active pocket of TERT, forming two π cation bonds with the protein amino acid residue ARG11, a hydrogen bond and a salt bridge with ARG15, a hydrogen bond with VAL21, and a *π–π* bond with the 148th C base of hTR. At 15 ns, an allosteric reaction occurred to form an unstable state, forming a hydrogen bond and a salt bridge with the protein amino acid residue ARG15, with the rest of the binding sites lost. At 100 ns, BIBR1532 re‐entered a new stable state by forming a hydrogen bond and a salt bridge with the protein amino acid residue ARG15, a hydrogen bond with ARG30, a hydrogen bond with the 60th G base of hTR, and four *π–π* bonds with the 62nd A base.

Root Mean Square Deviation (RMSD) was used to measure the average change in displacement of a selection of atoms for a particular frame with respect to a reference frame (Figure S34, Supporting Information). These results showed that the binding between BIBR1532 and TERT was stable after 30 ns, and the system was in equilibrium. Next, Root Mean Square Fluctuation (RMSF) was used for characterizing local changes along the protein chain. The protein residues that interacted with BIBR1532 were marked in Figure 3p. Molecular dynamics showed that the binding between the hTR template and the CTE portion of the telomerase protein in telomerase could be inhibited by BIBR1532, resulting in the decrease of telomerase activity (Figures S35–S40, Supporting Information). Moreover, TPP1 could specifically recruit telomerase to telomeres.^[^ ^24^, ^25^ ^]^ The binding between BIBR1532 and telomerase could cause the deviation of RNA from its optimal functional region, thus the binding between RNA and DNA was hindered, leading to the inhibition of RNA‐dependent DNA extension (Figure 3q).

**Figure 4:**
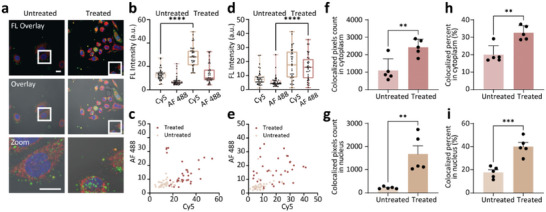
Expression of hTR and telomerase activity upon Sinefungin treatment. a) Confocal fluorescence images of HeLa cells incubated with DNA‐ND‐Lipo/DNA‐ND‐Lipo*_n_* and without or with Sinefungin treatment. FL Overlay: mixed fluorescence channel. Overlay: mixed fluorescence & bright‐field channel. Zoom: enlarged image of a single cell of mixed fluorescence. Scale bar: 20 µm. b) Fluorescent intensity of hTR (AF 488) and telomerase activity (Cy5) in cytoplasm region corresponding to cell images in (a). Each data point represents one cell (*n* = 30). c) Fluorescence distribution of hTR (AF 488) and telomerase activity (Cy5) in cytoplasm corresponding to cells without or with Sinefungin treatment. d) Fluorescent intensity of hTR (AF 488) and telomerase activity (Cy5) in the nucleus region corresponding to cell images in (a). Each data point represents one cell (*n* = 30). e) Fluorescence distribution of hTR (AF 488) and telomerase activity (Cy5) in the nucleus corresponding to cells without or with Sinefungin treatment. f) Colocalized pixels counted in cytoplasm of HeLa cells without or with Sinefungin treatment. Each dot represents the average of 5–10 cells (*n* = 5). g) Proportion of colocalized region in cytoplasm according to statistical data in (f). Each dot represents the average of 5–10 cells (*n* = 5). h) Colocalized pixels counted in the nucleus of HeLa cells without or with Sinefungin treatment. Each dot represents the average of 5–10 cells (*n* = 5). i) Colocalized pixels counted in the nucleus according to statistical data in (h). Each dot corresponded to the average of 5–10 cells (*n* = 5) ^**^ *p* < 0.01, ^***^ *p* < 0.001, ^****^ *p* < 0.0001).

Next, we tested another drug molecule, Sinefungin, to explore its influence on the cooperative expression of hTR and telomerase activity in HeLa cells. Sinefungin is the analog of S‐adenosylmethionine, which serves as a trimethylguanosine synthase 1 (TGS1) inhibitor to interfere with the maturation of hTR by blocking the formation of 2,2,7‐trimethylguanosine (TMG) cap.^[^ ^21^ ^]^ TGS1 is an important factor in stabilizing telomere‐Cajal body interactions, and negatively regulates hTR abundance and telomerase activity.^[^ ^26^ ^]^ Sinefungin can upregulate hTR expression and telomerase activity by reducing 2,2,7‐TMG capping. CCK‐8 assay of HeLa cells treated by Sinefungin showed negligible apoptosis (Figure S41, Supporting Information). From the confocal fluorescence images of HeLa cells before and after Sinefungin treatment for 7 days (**Figure** **4**a), the fluorescent area of AF 488 and Cy5 increased in both nucleus and cytoplasm, indicating upregulated hTR and telomerase activity by Sinefungin. The fluorescence intensity of AF 488 (hTR) and Cy5 (telomerase activity) also increased in both the nucleus and cytoplasm (Figure 4b). Moreover, cells without or with Sinefungin treatment could be easily discriminated by the fluorescence distribution of AF 488 (hTR) and Cy5 (telomerase activity) in cytoplasm (Figure 4c). Notably, obvious hTR accumulation in the nucleoli was observed (Figure 4d,e). Next, we checked the colocalized area in cytoplasm and nucleus of cells with or without Sinefungin treatment by using logic processing (Figure 4f–I; Figure S42, Supporting Information), which was in accordance with previous results. Thus, compared with cytoplasm, the colocalized percentage was significantly increased in the nucleus after Sinefungin treatment, indicating that Sinefungin could prevent the degradation of telomerase by guiding telomerase to Cajal bodies in the nucleus.

## 3 Conclusion

Telomerase‐related pathologies and telomerase activation in cancer has been a longstanding research focus. In this study, we constructed a DNA‐based sensing platform to in situ localize and image hTR and telomerase activity by integrating a target‐activatable DNA nanodevice (DNA‐ND). This platform possesses the rapid design capabilities of DNA nanotechnology, enabling the convenient localization and imaging of two commonly used telomerase markers in living cells. Notably, the fluorescence imaging comparison reveals that the locations of hTR and telomerase activity do not exactly overlap. This finding underscores the intricate spatial distribution of these two components within different subcellular areas, shedding light on the complexity of telomerase dynamics. One of the key advantages of our approach is the orthogonal fluorescence imaging of hTR and telomerase by DNA‐ND in living cells, thus it overcomes the limitations associated with the use of expensive immunofluorescence reagents and the complex operations involving lengthy cell culture periods for stable fluorescence expression. Moreover, our platform enables continuous observation of changes in cell state, providing valuable insights into the dynamic behavior of telomerase. The changes of cell state can also be observed continuously. Thus, we achieve an intriguing observation regarding the impact of BIBR1532 and Sinefungin treatment on the profile of hTR and telomerase activity in cancer cells. Overall, this work may bring new insights into the subtle interactions between hTR and telomerase at the subcellular level. Advanced therapeutic applications are also expected in future.^[^ ^1^ ^]^

## Conflict of Interest

The authors declare no conflict of interest.

## Supporting information



Supporting Information

## Data Availability

The data that support the findings of this study are available from the corresponding author upon reasonable request.
