# Supplementary material for: A Modular Engineered DNA Nanodevice for Precise Profiling of Telomerase RNA Location and Activity
Source: Adv Sci (Weinh). 2024 Dec 27;12(7):2409344. doi: 10.1002/advs.202409344 (PMC11831533; doi:10.1002/advs.202409344)
Supplement: Supplementary file 1 — Supporting Information [file ADVS-12-2409344-s001.docx]

**Supporting Information for**

**A modular engineered DNA nanodevice for precise profiling of telomerase RNA location and activity**

Shi-Yi Zhang,^[+, a]^ Jian Lv^[+, a]^, Ze-Rui Zhou^[+, a]^, Peter X. Geng^[b]^, Da-Wei Li^[a]^, Ruo-Can Qian*^[a]^ and Huangxian Ju*^[c]^

[a] S. Y. Zhang, Dr J. Lv, Dr Z. R. Zhou, Prof. D. W. Li, Dr R. C. Qian
Key Laboratory for Advanced Materials, Feringa Nobel Prize Scientist Joint Research Center, Joint International Laboratory for Precision Chemistry, Frontiers Science Center for Materiobiology & Dynamic Chemistry, School of Chemistry and Molecular Engineering
East China University of Science and Technology, Shanghai 200237, P. R. China
E-mail: ruocanqian@ecust.edu.cn

[b] P. X. Geng
Department of Biomedical Engineering, College of Future Technology
Peking University, Beijing 100871, P. R. China.

[c] Prof. H. X. Ju
State Key Laboratory of Analytical Chemistry for Life Science, School of Chemistry and Chemical Engineering
Nanjing University, Nanjing 210023, P. R. China.
E-mail: hxju@nju.edu.cn

[^+^] These authors contributed equally to this work.

TABLE OF CONTENTS

**Figure S1.** Detailed structure of DNA nanodevice ………………………………………………11

**Figure S2.** Mass spectra of DNA sequences for FL spectra and CLSM ………………………11

**Figure S3.** Mass spectra of DNA sequences for gel electrophoresis …………………………12

**Figure S4**. FL spectra of DNA-ND treated by heat-inactivated telomerase …………………13

**Figure S5.** FL spectra of DNA-ND treated with telomerase and unrelated RNA……………13

**Figure S6.** Detailed structure of DNA nanodevice in control experiment……………………14

**Figure S7.** Mass spectra of mismatched DNA sequence for control experiment……………14

**Figure S8**. FL spectra of mismatched DNA-ND …………………………………………………15

**Figure S9.** Mass spectra of mismatched DNA sequences for gel electrophoresis…………15

**Figure S10.** Specific recognition between DNA-ND and HeLa lysates evaluated by agarose gel electrophoresis.……………………………………………………………………16

**Figure S11**. Fluorescence responses of DNA-ND incubated with different concentrations of cytoplasm telomerase extracts………………………………………………………16

**Figure S12**. Fluorescence responses of DNA-ND incubating with different concentrations of nuclear telomerase extracts…………………………………………………………17

**Figure S13**. FL spectra of DNA strands for verifying the fluorescent stability…………………18

**Figure S14.** TEM images of liposomes……………………………………………………………18

**Figure S15.** Confocal images of control group using DNA-ND without liposomes……………19

**Figure S16**. Optimization of NLS concentration …………………………………………………19

**Figure S17.** Dynamic light scattering (DLS) characterization for the modification processing of DNA-ND-Lipo and DNA-ND-Lipo/DNA-ND-Lipo*_n_* ………………………………20

**Figure S18.** Optimization of incubation time ……………………………………………………21

**Figure S19.** Optimization of DNA-ND-Lipo/DNA-ND-Lipo*_n_* concentration……………………22

**Figure S20.** Negative control using mismatched DNA-ND-Lipo/DNA-ND-Lipo*_n_* ……………23

**Figure S21.** Fluorescence intensity between control and experimental groups………………23

**Figure S22.** Cytotoxicity of DNA-ND-Lipo ………………………………………………………24

**Figure S23.** Cytotoxicity of DNA-ND-Lipo in HeLa cells by confocal fluorescent images..…24

**Figure S24.** Cytotoxicity of DNA-ND-Lipo/DNA-ND-Lipo*_n_*………………………………………25

**Figure S25.** Cytotoxicity of DNA-ND-Lipo/DNA-ND-Lipo*_n_* by confocal fluorescent images…25

**Figure S26.** Schematic diagram of fluorescence distribution analysis using logic gates …26

**Figure S27.** Cytotoxicity of BIBR1532 ……………………………………………………………27

**Figure S28.** Logic output of HeLa cells with and without BIBR1532 treatment ………………27

**Figure S29.** Detailed ligand atom interactions with the TERT residues ………………………28

**Figure S30.** Template/pseudoknot of hTR and its interaction with TERT TEN domains in state 1 …………………………………………………………………………………29

**Figure S31.** Template/pseudoknot of hTR and its interaction with TERT TEN domains in state 2………… ………………………………………………………………………30

**Figure S32.** Template/pseudoknot of hTR and its interaction with TERT TEN domains in state 3 …………………………………………………………………………………31

**Figure S33.** Template/pseudoknot of hTR and its interaction with TERT TEN domains in state 4 …………………………………………………………………………………32

**Figure S34.** RMSD results for TERT and BIBR1532 …………………………………………33

**Figure S35.** Protein secondary structure ………………………………………………………34

**Figure S36.** Ligand RMSF ………………………………………………………………………35

**Figure S37.** Contribution of amino acids at binding sites to TERT binding …………………36

**Figure S38.** BIBR1532 interactions with specific amino acids of TERT over time ……………37

**Figure S39.** Ligand torsion diagram and dial (or radial) diagram ………………………………38

**Figure S40.** Ligand properties ……………………………………………………………………39

**Figure S41.** Cytotoxicity of Sinefungin ……………………………………………………………40

**Figure S42.** Logic output images of HeLa cells with and without Sinefungin treatment.……40

**Table S1.** DNA sequences….………………………………………………………………………41

**Table S2.** DNA sequences for gel electrophoresis ………………………………………………41

**Table S3.** Mismatched DNA sequences …………………………………………………………41

**Table S4.** Experimental parameters in CLSM experiments ……………………………………42

**Table S5.** XP&MM-GBSA results …………………………………………………………………42

**Table S6.** BIBR1532 changes in binding energy with TERT……………………………………42

**Table S7.** Model of TERT used in molecular docking and MD simulation……………………43

**MATLAB code used in logic analysis**…………………………………………………………43

**Supplementary Reference**………………………………………………………………………48

**Methods**

**Materials and reagents**

75 mM KH_2_PO_4_, 137 mM NaCl and 2.65 mM KCl. 1× TE buffer (pH 7.8) contained 10 mM/L Tris-HCl and 1 mM EDTA. Lipofectamine 2000 was purchased from ThermoFisher Scientific. All aqueous solutions were prepared using ultrapure water (≥ 18 MΩ, Milli-Q, Millipore). Dulbecco’s modified Eagle medium (DMEM), FBS, penicillin G and streptomycin and trypsin, nuclear/cytoplasmic protein extraction kit, dNTPs, CCK-8 kit and 0.4% trypan blue used in cell experiments were purchased from Sangon Biological Engineering Technology Co., Ltd. (Shanghai, China). Cell lysis buffer was purchased from Shanghai Macklin Biochemical CO., LTD, containing 20 mM Tris (pH 7.5), 150 mM NaCl, 1% Triton X-100, sodium pyrophosphate, β-glycerophosphate, EDTA, Na_3_VO_4_, and leupeptin. 10× Tris Borate EDTA (TBE) buffer, agarose gel, SYBR Gold nucleic acid gel stain, ultra low range DNA ladder and sample loading buffer used in gel electrophoresis were purchased from ThermoFisher Scientific. BIBR1532 and Sinefungin were purchased from AbMole BioScience. BIBR1532 was diluted with DMSO. DMSO was purchased from LingFeng Chemical Co., Ltd. All DNA sequences and peptides were purchased from Sangon Biological Engineering Technology Co., Ltd. (Shanghai, China). DNA sequences are given in Supplementary Information and their characterizations are provided in Figure S2, 3, 7 and 9.

**Instruments**

Fluorescence spectra were recorded on an F97pro spectrometer (Shanghai Lengguang Technology Co., Ltd.). The fluorescent images of human telomerase RNA and telomerase activity were captured by confocal fluorescent scanning microscope (Nikon A1R, Japan). Cell viability was examined on a microplate reader (Synergy 5, Biotech, USA). Flow cytometric analysis was performed using a NovoCyte 2060R flow cytometer (Agilent Technologies Inc., USA). The gel was imaged on a Bio-Rad molecular imager under blue light.

**Construction of DNA-ND-Lipo and DNA-ND-Lipo/DNA-ND-Lipo*_n_***

DNA probes were annealed in 1 × TE buffer at 94 °C for 2 min and then gradually cooled down at room temperature to obtain the desired structure. 2 μL 1 mg/L lipofectamine 2000 was applied to encapsulate the DNA-ND and construct DNA-ND-Lipo. Briefly, after 2 μL lipofectamine 2000 was added in 50 μL DMEM (no FBS) for about 5 min, and DNA-ND of different concentrations was added in 50 μL DMEM, they were mixed for 30 min to obtain DNA-ND-Lipo. 0.5 mg/L NLS was modified on the liposome to construct DNA-ND-Lipo*_n_*. The solution was finally mixed in a 1 : 1 ratio (by volume) with a final volume of 220 μL. The concentration was calculated by the DNA.

**Cell culture**

HeLa cells were cultured with DMEM containing 10% FBS. MCF-7 cells were cultured with MEM containing 10% FBS and 10 μg/mL Insulin. LO2 normal cells were cultured with RPMI-1640 supplemented with 10% FBS. All the media contain penicillin (100 µg mL^-1^), and streptomycin (100 µg mL^-1^). Cells were cultured at 37 °C in a humidified atmosphere containing 5% CO_2_. HeLa cells were kindly provided by Stem Cell Bank, Chinese Academy of Sciences. MCF-7 cells were kindly provided by Procell Life Science&Technology Co., Ltd. LO2 normal cells were purchased from Fu Heng Biology Co., Ltd.

**Nuclear telomerase extraction**

The extraction of nuclear telomerase was operated according to the protocol. In short, the cells were scraped and centrifuged at 1000 g for 3 min at 4 °C. The resulting precipitate was dissolved in Buffer A. 200 μL Buffer A (1 mL Buffer A + 1 μL DTT + 10 μL PMSF +1 μL protease inhibitor) was added in 20 μL of cells and allowed to stand on ice for 10 min. 11 μL Buffer B was then added and stood on ice for 4 min. The sample was centrifuged at 14000 g for 5 min at 4 °C. The resulting supernatant contained the cytoplasmic fraction. The resulting middle pellet was resuspended in 100 μL Buffer C (1 mL Buffer C + 1 μL DTT + 10 μL PMSF +1 μL protease inhibitor) and placed in a shaker and ice bath for 40 min. After centrifugation at 14000 g for 5 min at 4 °C, the resulting supernatant was obtained as the nuclear fraction. The corresponding nuclear/cytoplasmic fractions were packaged and stored at -80 °C to avoid repeated freezing and thawing. DNA-ND was incubated with the nuclear telomerase extracts for 4 h at 37 °C for following experiments.

**Detection of telomerase activity in cell lysates**

The cells were collected in the exponential phase of growth and counted with Beckman cell counter. Approximately, 1.0 × 10^6^ cells were centrifuged at 2000 rpm for 5 min and washed twice with PBS (1×, pH 7.4) at 4 °C. Then, the cells were lysed in 200 μL ice-cold cell lysis buffer for 30 min on ice. The lysate was centrifuged at 12000 rpm for 20 min at 4 °C. The supernatant was transferred carefully and stored at -80 °C for further experiments. As control, the telomerase extract was heat-treated at 95 °C for 10 min prior to the detection. For the detection of telomerase activity, 10 µL of 10 mM dNTPs, 100 µL telomerase extract solution and 100 nM DNA were mixed in 1× PBS and incubated at 37 °C for different times to measure the change of fluorescence intensity.

**Gel electrophoresis analysis**

3% agarose gel was first prepared in 0.5 × TBE buffer, run at 70 V for 40 min, and then stained with 7 μL SYBR Gold nucleic acid gel stain. Finally, the gel was imaged using a Bio-Rad molecular imager under blue light. In detail, the strands were diluted with 1 × PBS solution to 1 μM, and 1 μL loading buffer was added to 5 μL strand solution in each lane. The indicated DNA in lanes 9, 10 and 11 was diluted to 1 μM after mixing with cell lysates at 37 °C for 2 h in each lane. The DNA sequences are given in Supplementary Information and their characterizations are provided in Figure S3 and 9.

**Flow cytometric analysis**

HeLa cells, MCF-7 cells and LO2 normal cells were seeded in a 6-well plate (NEST Biotechnology, China) at a density of 5 × 10^5^ cells per well. After treated with 300 nM DNA-ND-Lipo and DNA-ND-Lipo/DNA-ND-Lipo*_n_* for 2 h, the cells were washed 3 times with PBS and detached with trypsin. After centrifugation (1000 rpm, 5 min), the cells were resuspended in 500 μL PBS for flow cytometry analysis.

**Confocal fluorescent images of cells**

About 5 × 10^4^ cells were seeded in a confocal dish (NEST Biotechnology, China). After incubation with solution, confocal fluorescence images were taken by confocal microscope. For the blue (Hoechst 33342) channel, 405-nm laser was utilized and the emissions were collected from 425 to 475 nm. For the green (AF 488) channel, 488-nm laser was utilized to collect the emissions from 500 to 550 nm. For the Cy5 channel, 561-nm laser was utilized to collect the emissions from 662 to 737 nm.

**Evaluation of cytotoxicity of DNA-ND-Lipo and DNA-ND-Lipo/DNA-ND-Lipo*_n_***

The cytotoxicity was evaluated by CCK-8 counting kit. Briefly, after HeLa cells (100 µL, 1.0 × 10^5^ mL^-1^) were seeded in the wells of 96-well plate for 24 h, the medium was discarded. Then the cells were incubated with 100 µL culture medium containing DNA-ND-Lipo and DNA-ND-Lipo/DNA-ND-Lipo*_n_* for different times, while the cells were incubated with 100 µL culture medium without DNA nanodevices as control. After washing with PBS (1×, pH 7.4), 100 µL medium (without FBS) and 10 µL CCK-8 was added to each well. After incubation at 37 °C for 2 h, the absorbance of each well was measured by using microplate reader at 450 nm. The relative cell viability (%) was calculated by (A_test_/A_control_) ×100. The concentration was calculated based on DNA.

**Evaluation of cytotoxicity of drugs**

The cytotoxicity of BIBR1532 and Sinefungin was examined by CCK-8 counting kit. Briefly, after HeLa cells (100 µL, 1.0 × 10^5^ mL^-1^) were seeded in the wells of 96-well plate for 24 h, the medium was discarded. The cells were then incubated with 100 µL culture medium containing BIBR1532 or Sinefungin of different concentrations and 1% DMSO for 24 h. The cells were incubated with 100 µL culture medium without BIBR1532 as control. After washing with PBS (1×, pH 7.4), 100 µL medium (without FBS) and 10 µL CCK-8 was added to each well. After incubation at 37 °C for 2 h, the absorbance of each well was measured by using a microplate reader at 450 nm. The relative cell viability (%) was calculated by (A_test_/A_control_) ×100.

**Statistical Analysis**

The fluorescence intensity values for each cell were measured using Image J, following adjustments to ensure uniform luminance parameters across all fluorescence images. The sample size (n) for each experiment is provided in the respective figure legends. Data are presented as mean ± SEM. Statistical analysis was conducted using a two-sided Student’s t-test in GraphPad Prism 9. Statistical significance was determined as follows: **P*<0.05, ***P*<0.01, ****P*<0.001 and ****: *P*<0.0001.

**Protein pretreatment**

The crystal structure of TERT catalysis core was obtained from RCSB PDB database. (PDB: 7TRD) Protein preprocess, regenerate states of native ligand, H-bond assignment optimization, protein energy minimization, and removal of waters were performed separately on the obtained protein crystals using the Protein Preparation Wizard module of Schrodinger Software.

**Ligand pretreatment**

The 2D SDF structure file of compound BIBR1532 was processed by LigPrep module in Schrodinger, and all 3D chiral conformations were generated.

**Identification of active sites**

The best binding site was predicted by the SiteMap module in Schrödinger, and then by the Receptor Grid Generation module in Schrödinger, the Enclosing box was set to wrap the predicted binding sites perfectly. On this basis, the active site of TERT was obtained.

**Molecular docking**

The processed ligand compound BIBR1532 was docked with the active sites of the RNA & protein complex (with the highest precision XP docking). At lower score, the free energy of binding between the compound and the protein was lower, and the stability of binding was better.

**MM-GBSA analysis**

The number of active sites of the complex of the ligand compound BIBR1532 and TERT was calculated and analyzed by MM-GBSA. MM-GBSA DG Bind could approximately represent the binding free energy of small molecules to protein, and the lower the binding free energy is, the higher the binding free energy is, indicating that the binding stability of the ligand to the protein is higher.

**MD Simulation**

To further optimize the binding mode of compound-protein complex, we performed conventional molecular dynamics simulations using the Desmond program. The OPLS4 force field was employed to parameterize the protein and small molecules, while the SPCE model was used for the water solvent. The compound-protein complex was placed in a cubic water box and solvated. The system's charge was neutralized by adding 0.150 M chloride and sodium ions. The energy of the system was initially minimized using the steepest descent minimization method for 50,000 steps. Subsequently, the positions of heavy atoms were restrained for NVT and NPT equilibration for an additional 50,000 steps. The system temperature was maintained at 300 K, and the system pressure was maintained at 1 bar. After completing the two equilibration stages, an unrestricted simulation was performed for 100 ns. The interactions were analysed, and dynamic trajectory animations were generated using Maestro 2023.


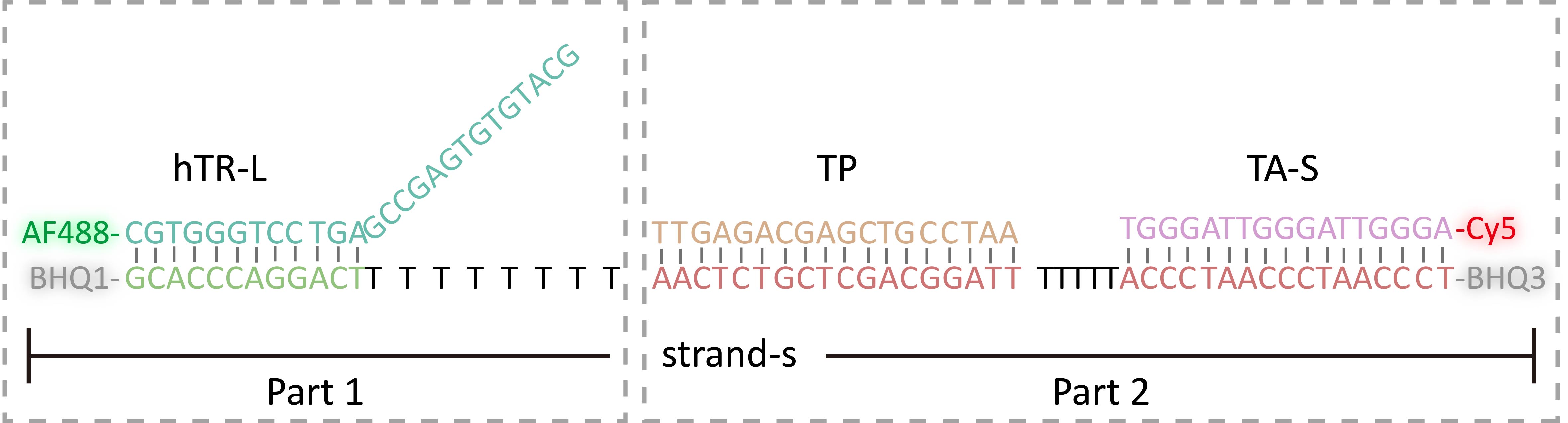


**Figure S1.** Detailed structure of DNA nanodevice.

**
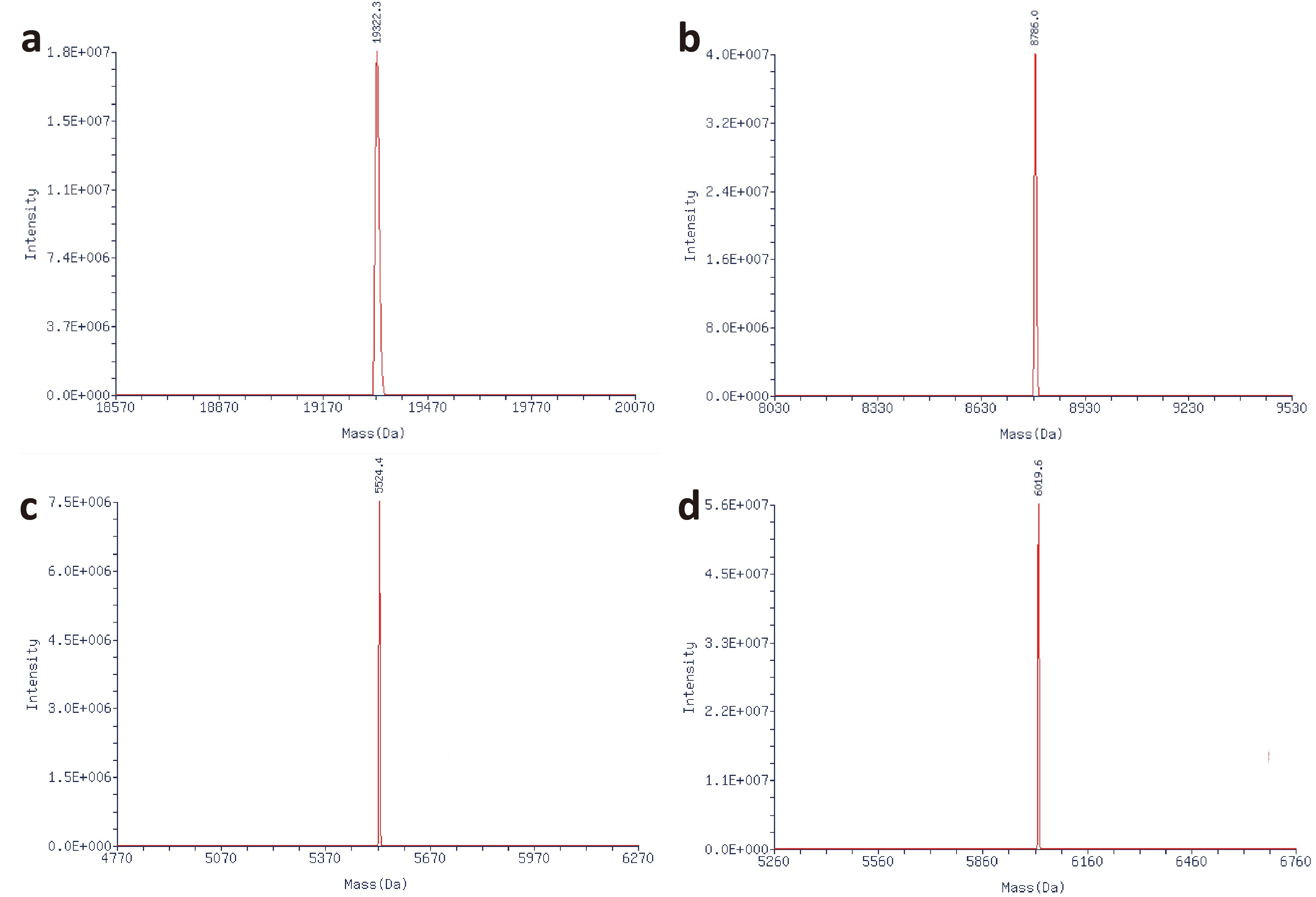
**

**Figure S2.** Mass spectra of DNA sequences used for FL spectra and CLSM. a, Strand-S. b, hTR-L. c, TP. d, TA-S.

**
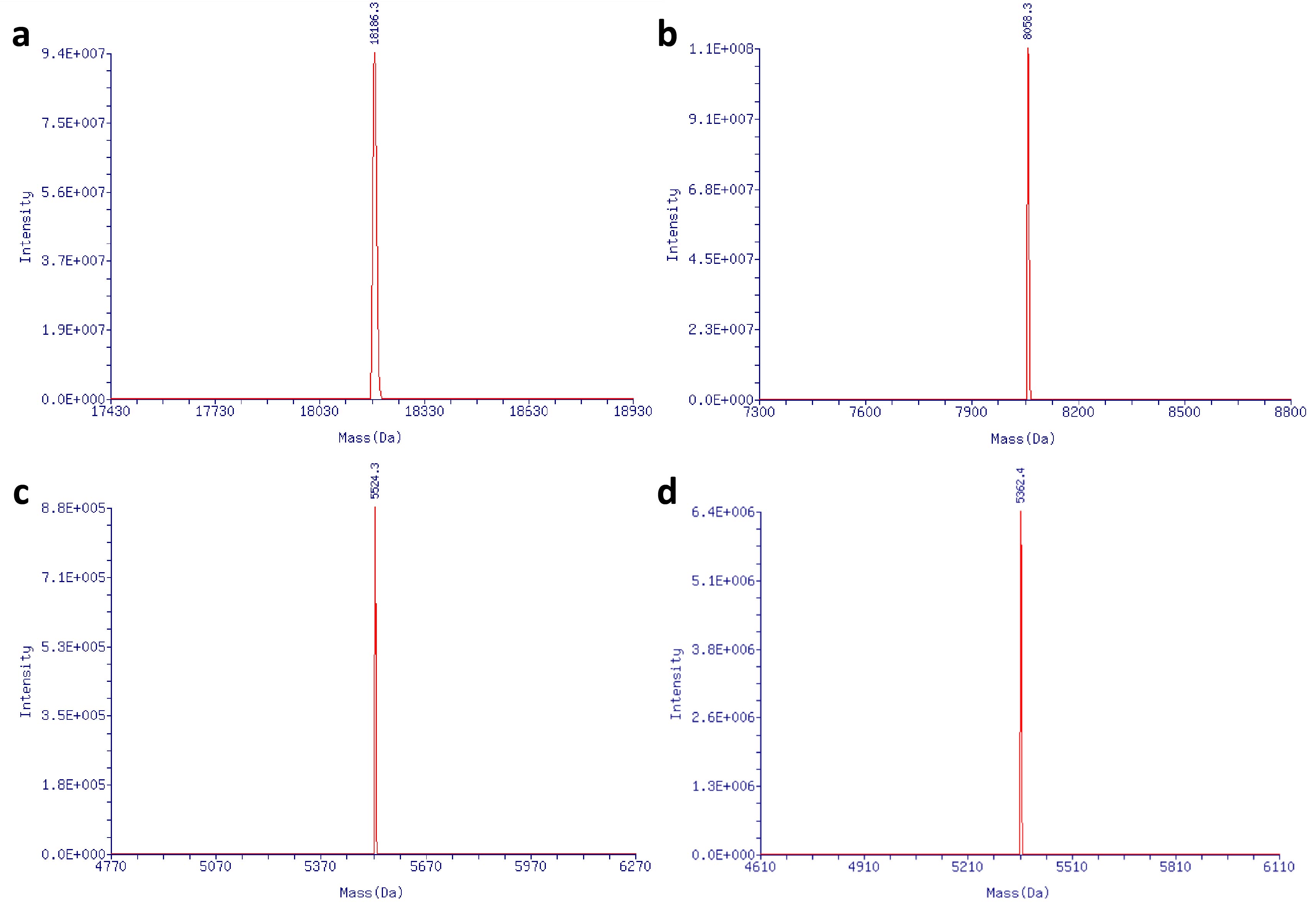
**

**Figure S3.** Mass spectra of DNA sequences used for gel electrophoresis. a, Strand-S. b, hTR-L. c, TP. d, TA-S.


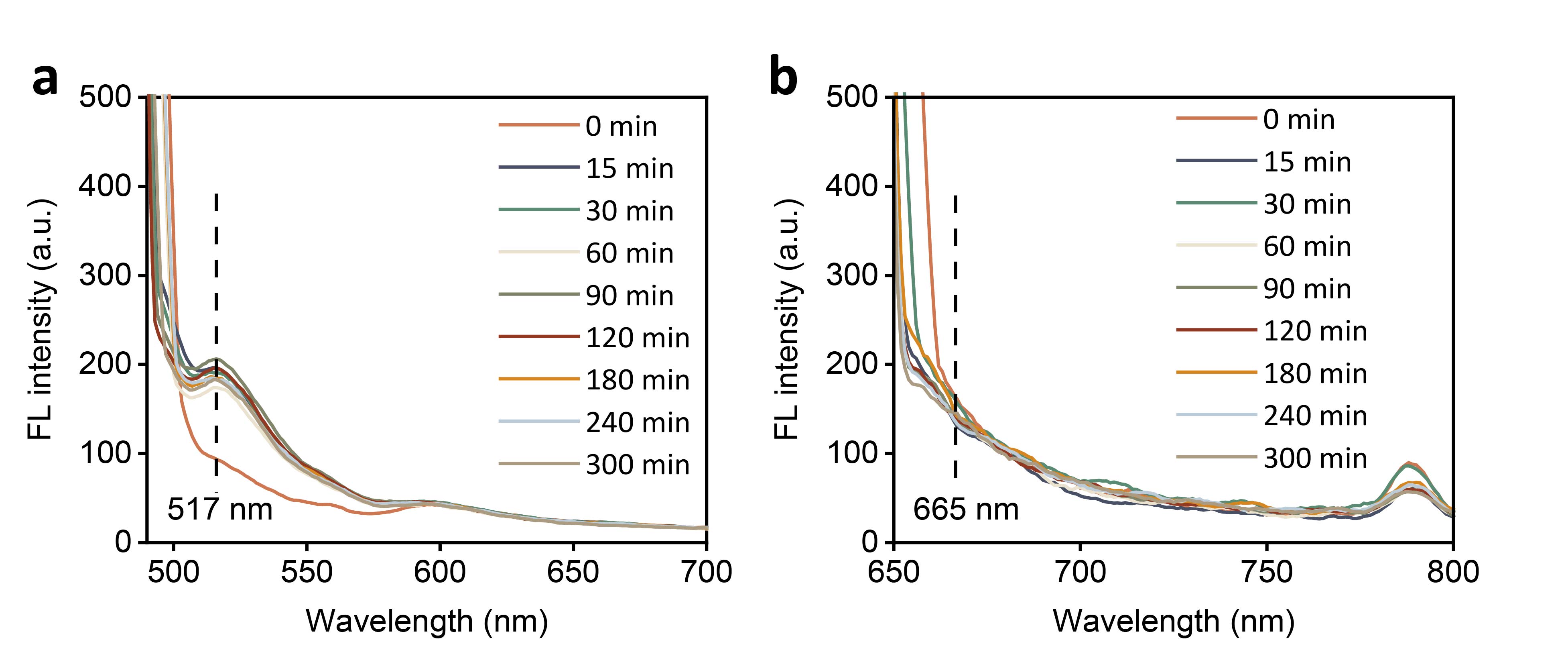


**Figure S4.** FL spectra of DNA-ND treated with heat-inactivated telomerase. a,b AF 488 (excitation: 470 nm) (a) and Cy5 (excitation: 630 nm) (b) of DNA-ND in 1× PBS solution treated by heat-inactivated lysates for different times (from 0 min to 420 min). Control experiments in the presence of heat-inactivated telomerase at an excitation of 470 nm (a) or 630 nm (b).


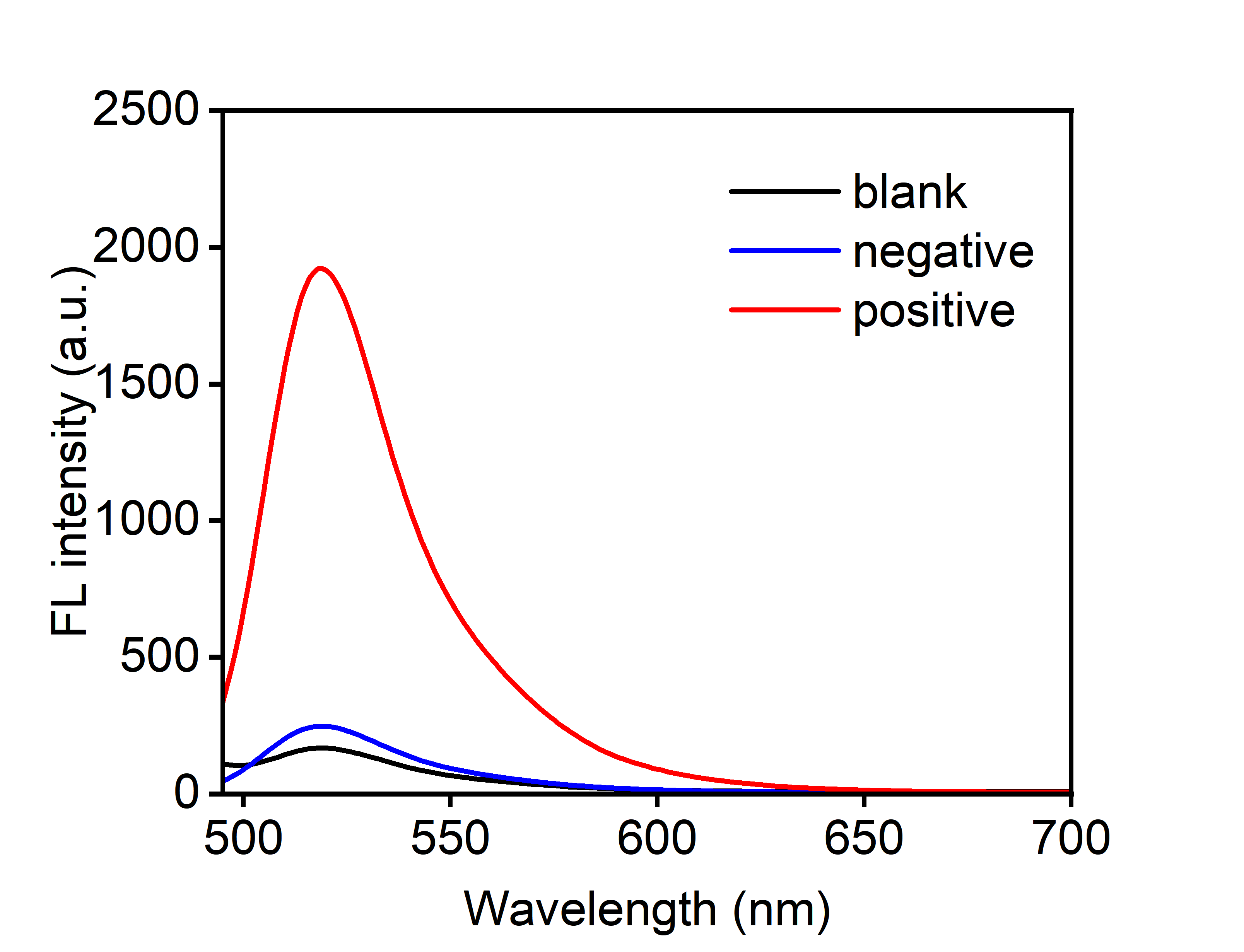


**Figure S5.** FL spectra of DNA-ND at AF 488 (excitation: 470 nm). DNA-ND was incubated with HeLa cell lysates (positive) and unrelated RNA (negative).


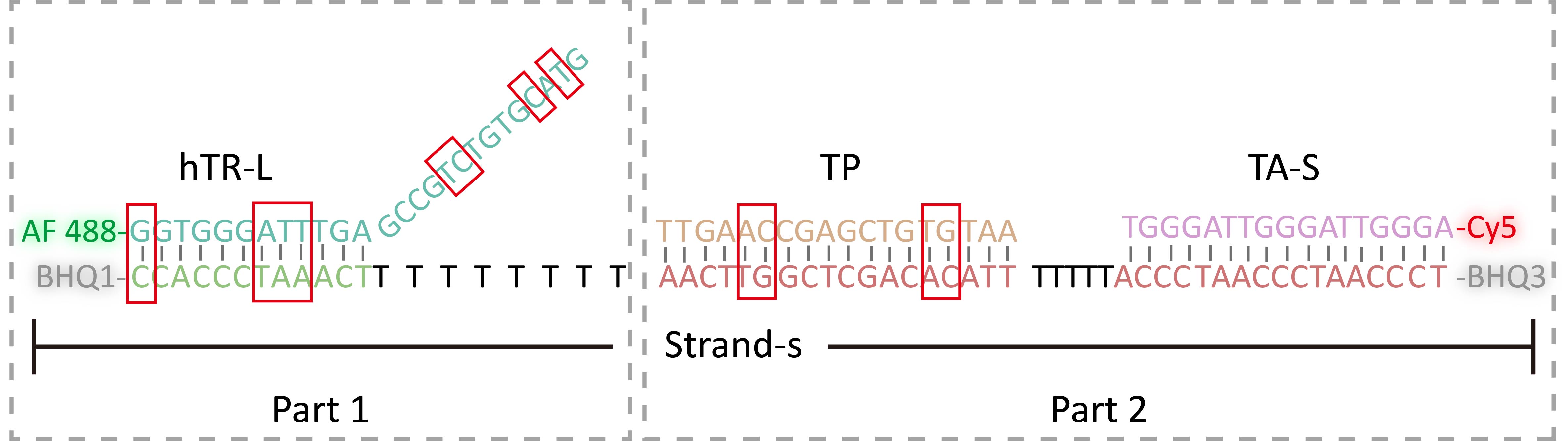


**Figure S6.** Detailed structure of DNA nanodevice in control experiment.

**
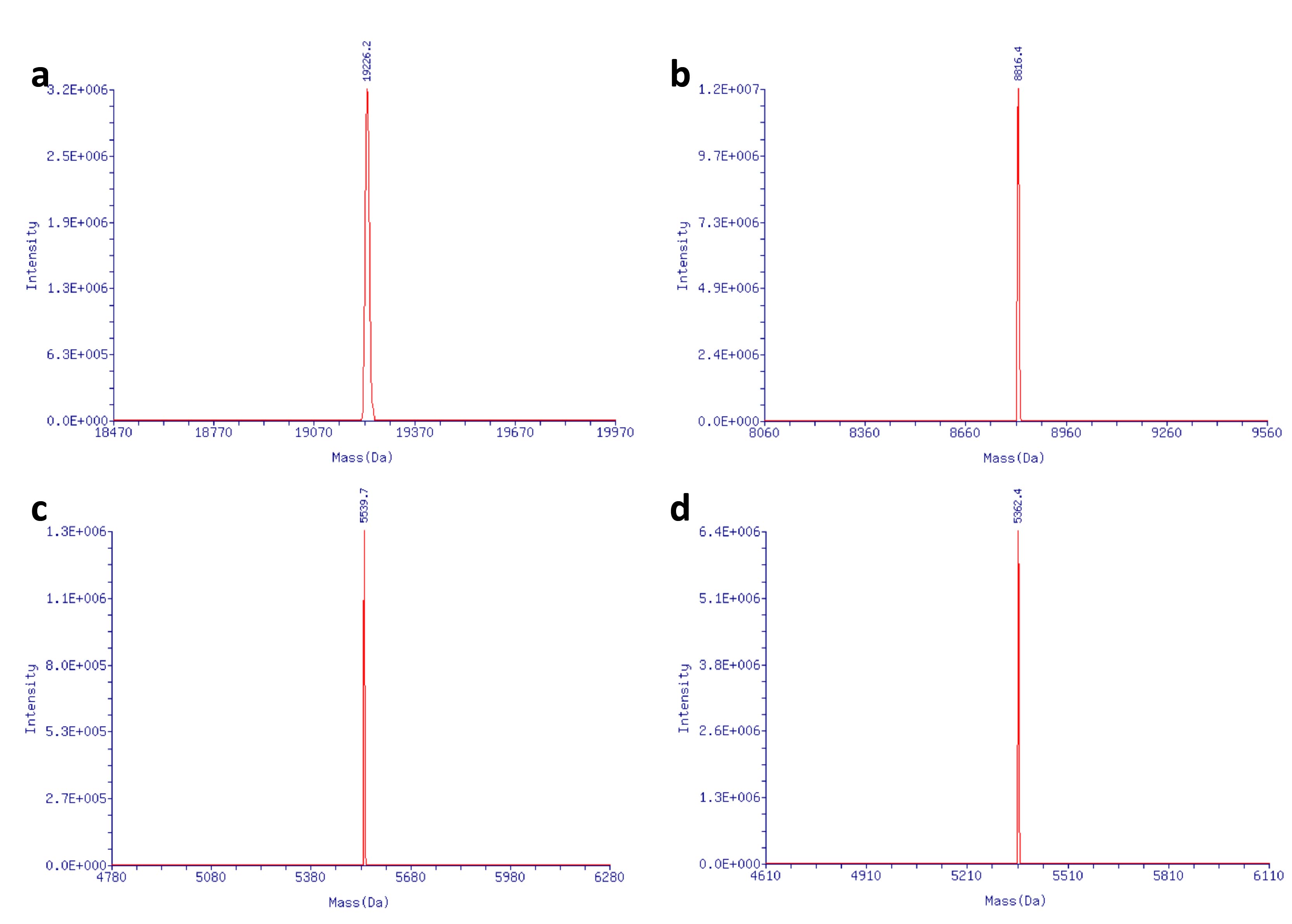
**

**Figure S7.** Mass spectra of mismatched DNA sequences for control experiment. a, Strand-S. b, hTR-L. c, TP. d, TA-S. These sequences were used for FL spectra and CLSM in Figure S8,13.


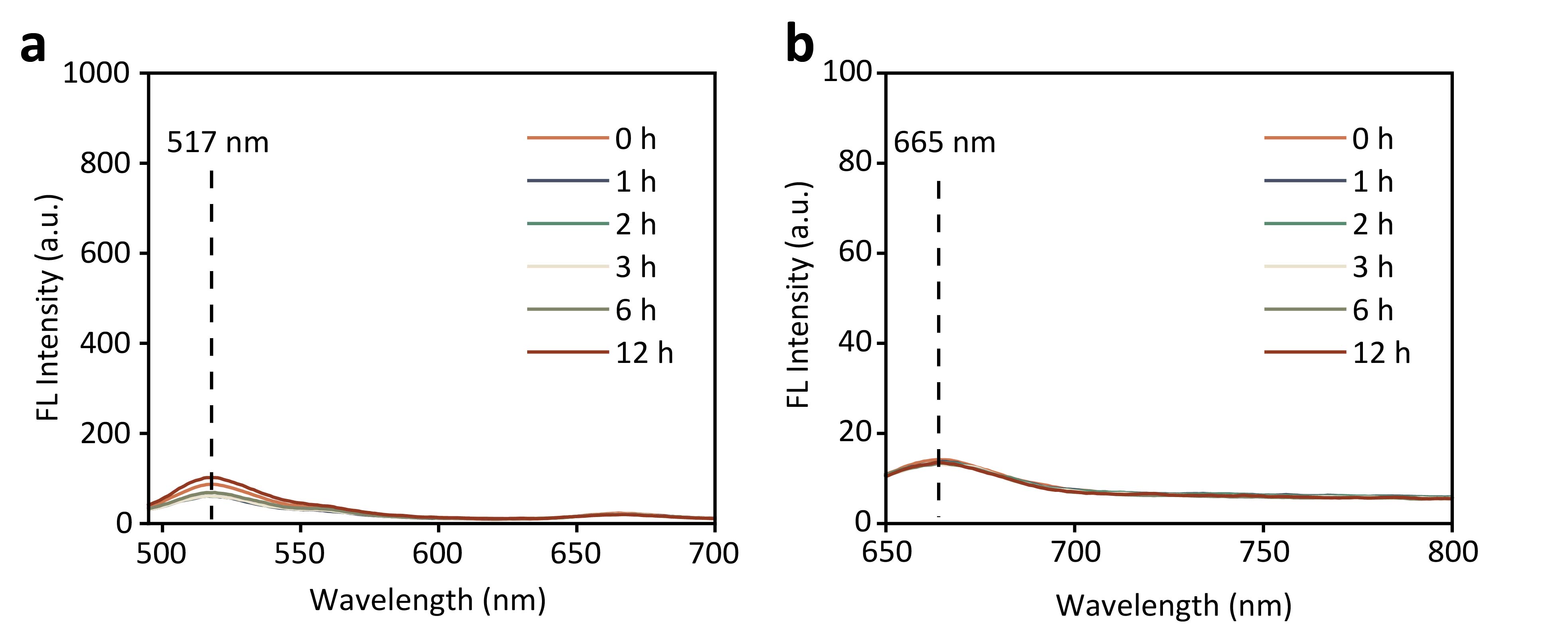


**Figure S8.** FL spectra of mismatched DNA-ND. a,b, Fluorescence spectra of AF 488 (excitation: 470 nm) (a) and Cy5 (excitation: 630 nm) (b) of mismatched DNA-ND in 1× PBS solution treated by HeLa cell lysates for different times (from 0 h to 12 h). The similar fluorescent intensity indicated that the DNA strand was not opened.

**
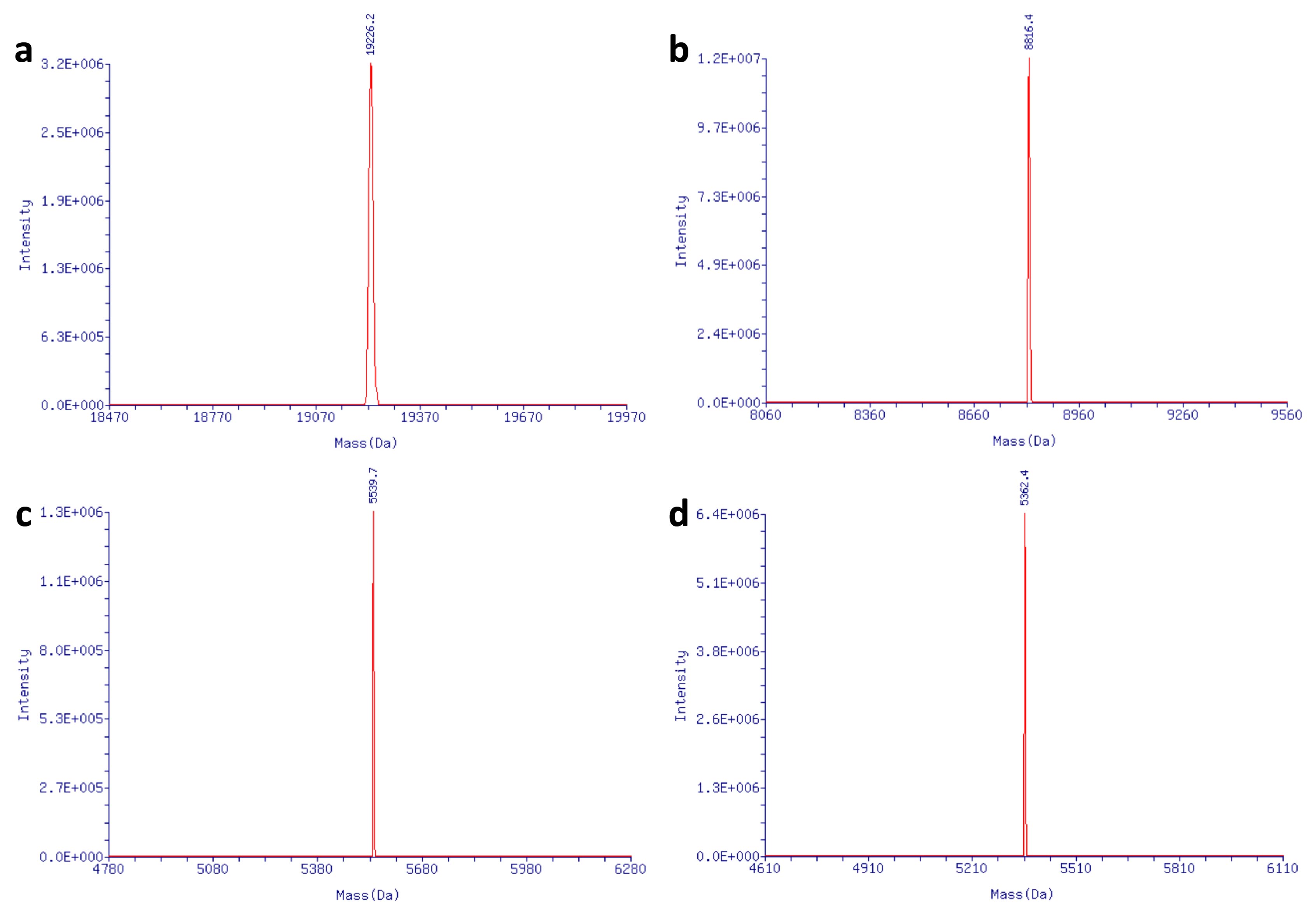
**

**Figure S9.** Mass spectra of mismatched DNA sequences used for gel electrophoresis. a, Strand-S'. b, hTR-L'. c, TP'. d, TA-S. These sequences were used for the control experiment in Figure S10.

**
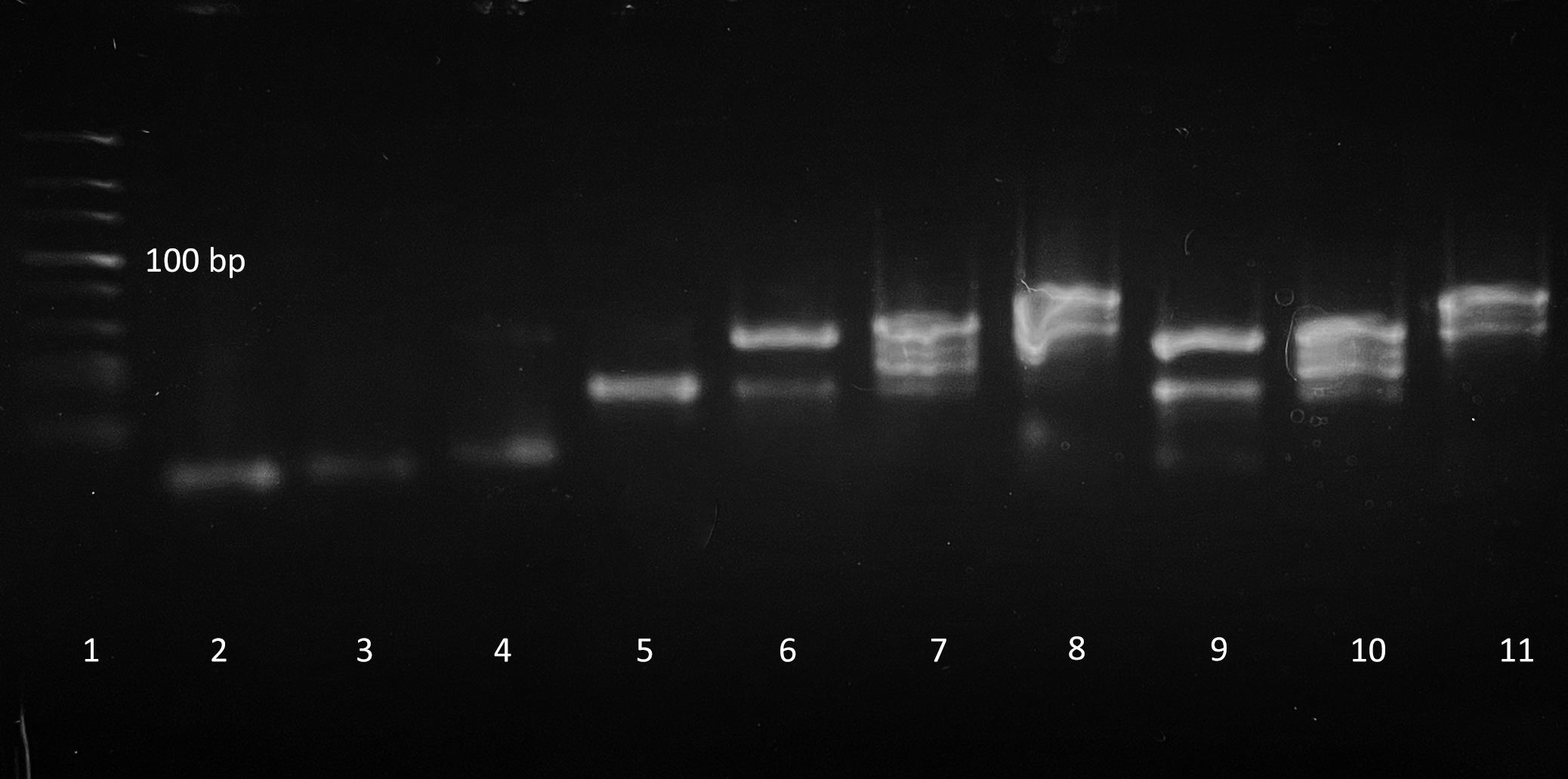
**

**Figure S10.** Specific recognition between DNA-ND and HeLa lysates evaluated by agarose gel electrophoresis. Lane 1, DNA ladder. Lane 2, TP'. Lane 3, TA-S. Lane 4, hTR-L'. Lane 5, Strand-S'. Lane 6, hybridized strands formed by hTR-L' and Strand-S'. Lane 7, hybridized strands formed by TP', TA-S, and Strand-S'. Lane 8, mismatched DNA-ND. Lane 9-11, samples of hybridized strands corresponding to Lane 6-8 treated by HeLa cell lysates at 37 °C for 2 h.

**
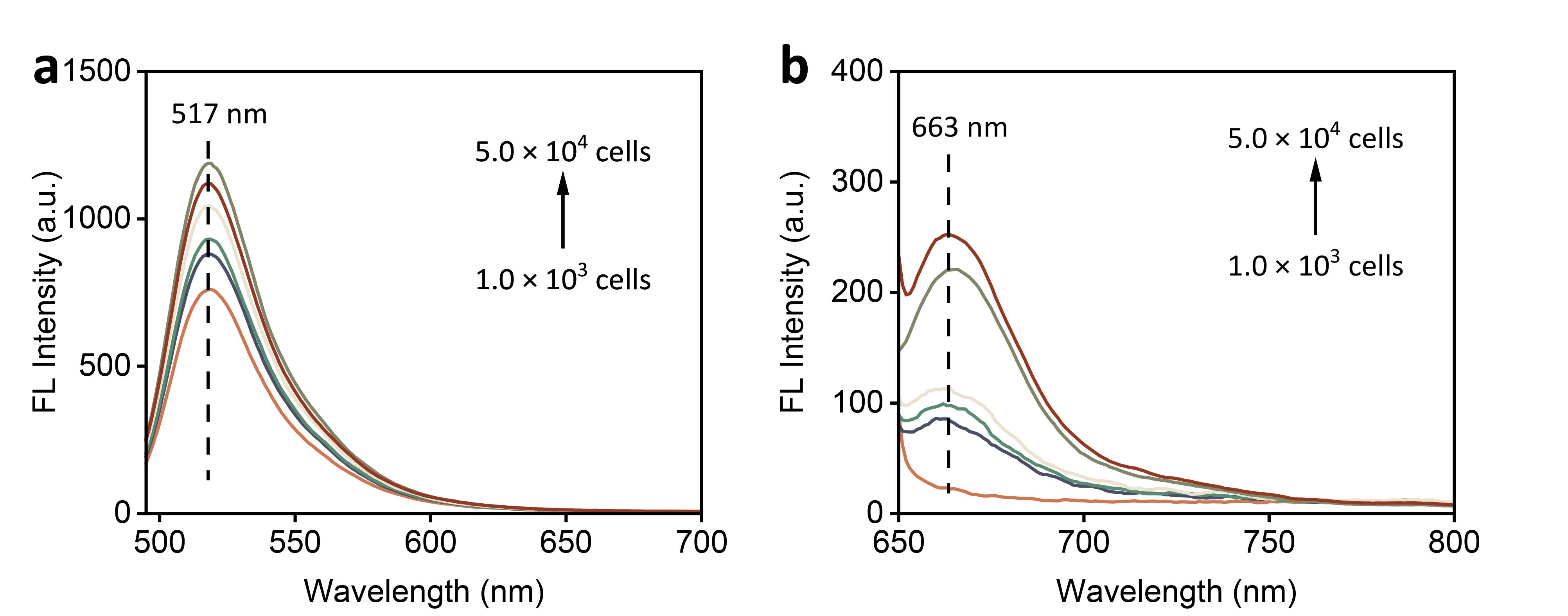
**

**Figure S11.** Fluorescence responses of DNA-ND incubating with different concentrations of cytoplasm telomerase extracts. a, b, Fluorescence spectra of AF 488 (excitation: 470 nm) (a) and Cy5 (excitation: 630 nm) (b) of 300 nM DNA-ND in 1× PBS solution treated with cytoplasm telomerase extracts from 1.0 × 10^3^ to 5.0 × 10^4^ at 37 °C for 2 h.

**
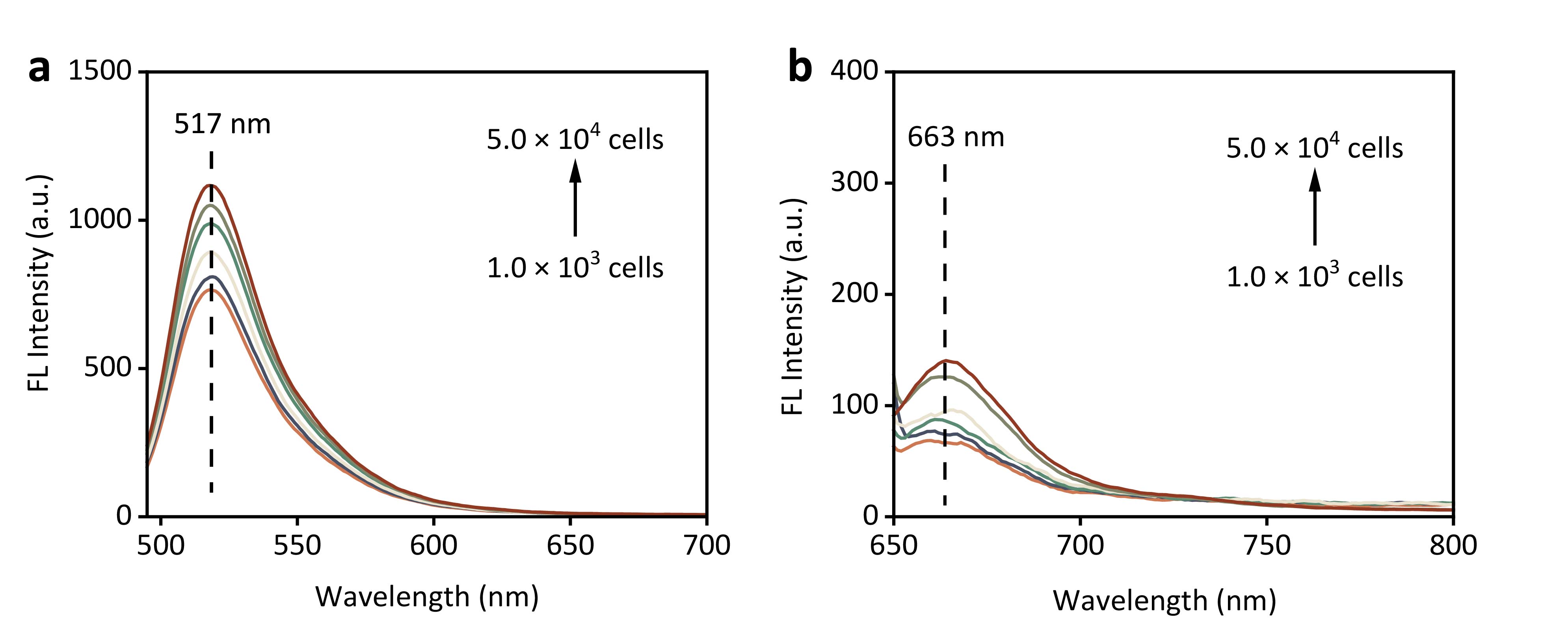
**

**Figure S12.** Fluorescence responses of DNA-ND incubating with different concentrations of nuclear telomerase extracts. a, b, Fluorescence spectra of AF 488 (excitation: 470 nm) (a) and Cy5 (excitation: 630 nm) (b) of 300 nM DNA-ND in 1× PBS solution treated with nuclear telomerase extracts from 1.0 × 10^3^ to 5.0 × 10^4^ at 37 °C for 2 h.

**
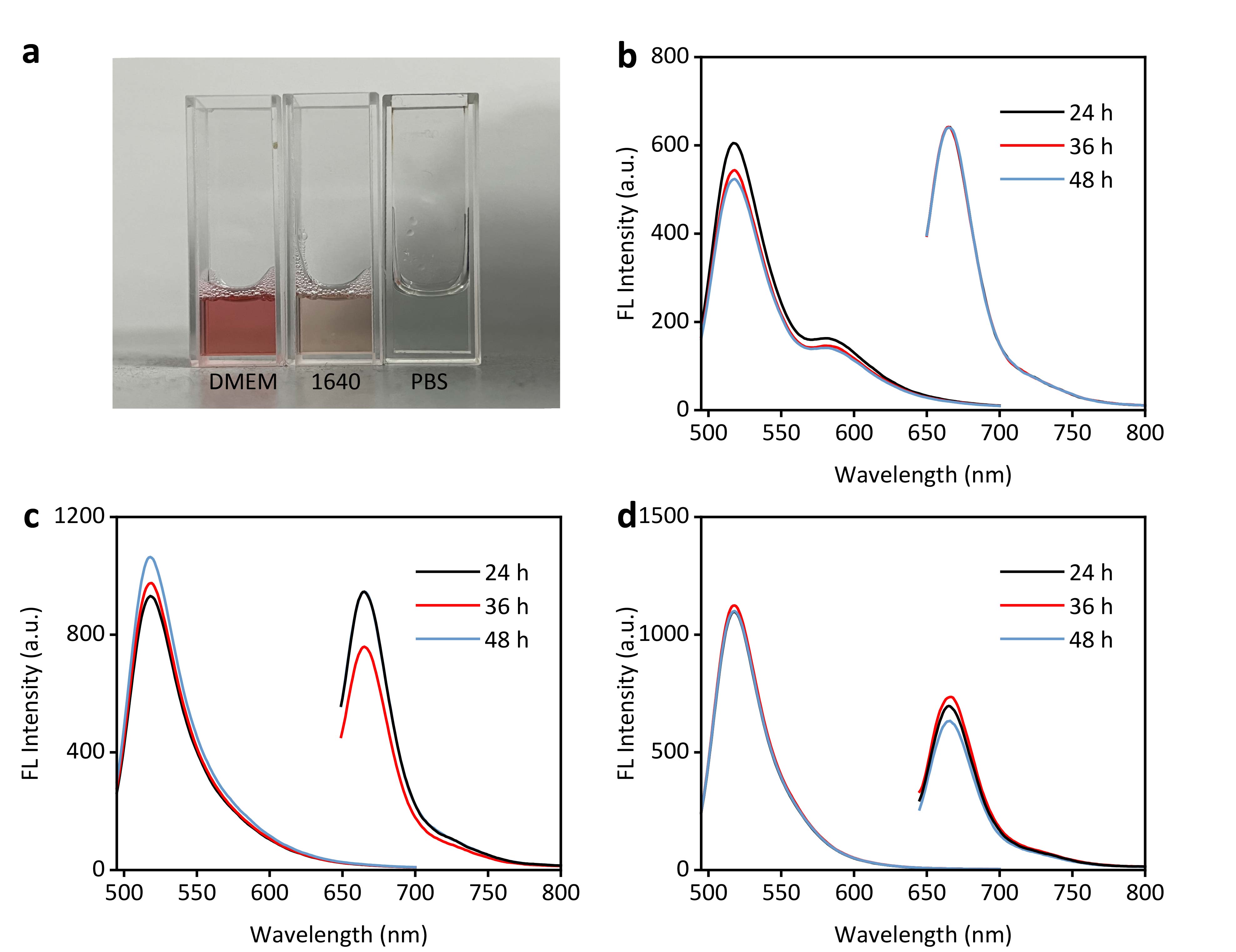
**

**Figure S13.** FL spectra of DNA strands for verifying DNA stability. a, Photos of DNA in different solutions. From left to right: DMEM medium, RPMI 1640 medium and 1× PBS solution. b-d, Fluorescence spectra of AF 488 (excitation: 470 nm) and Cy5 (excitation: 630 nm) of 100 nM hTR-L and TA-S in DMEM medium, RPMI 1640 medium and 1× PBS solution at 37 °C for different times.

**
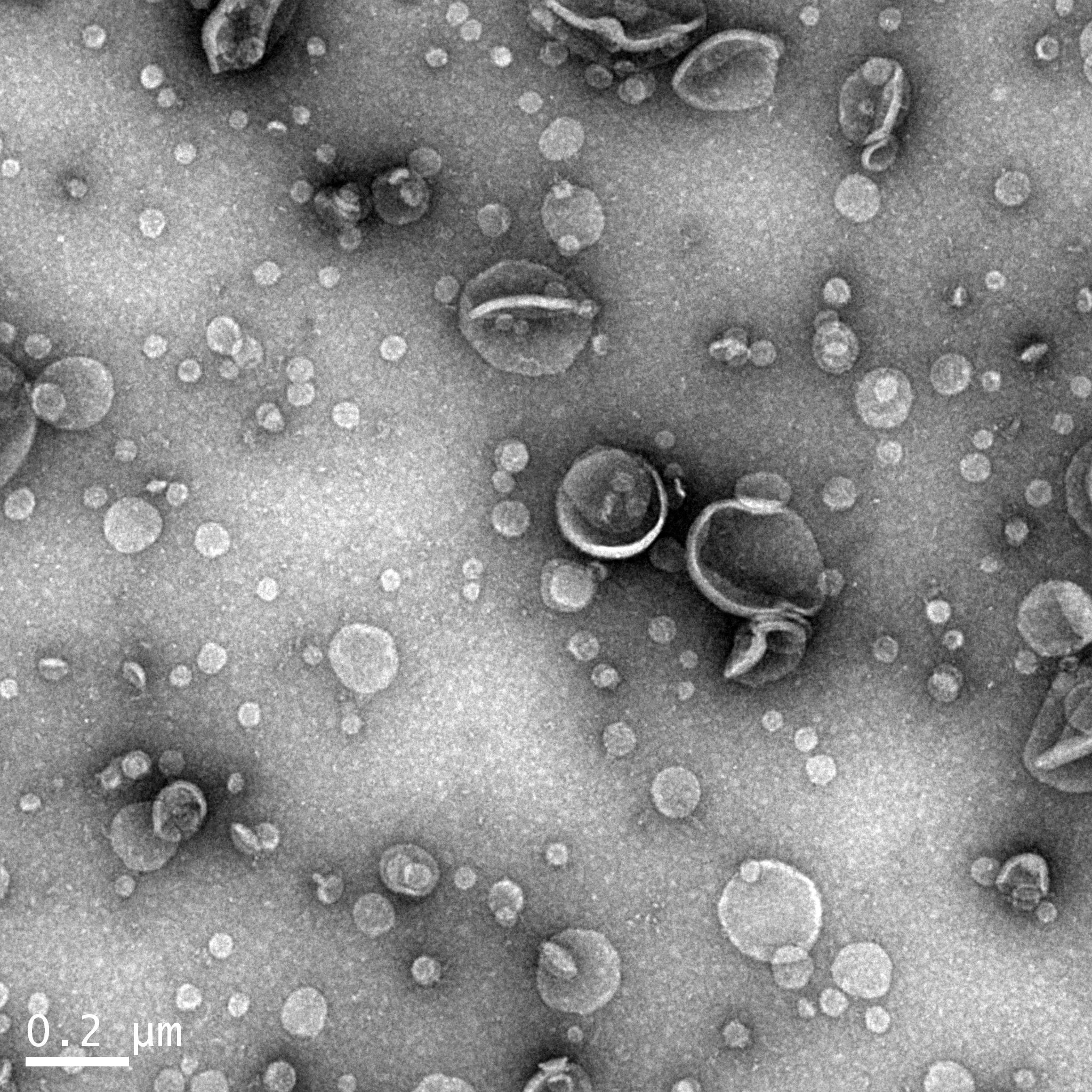
**

**Figure S14.** TEM images of liposomes.

**
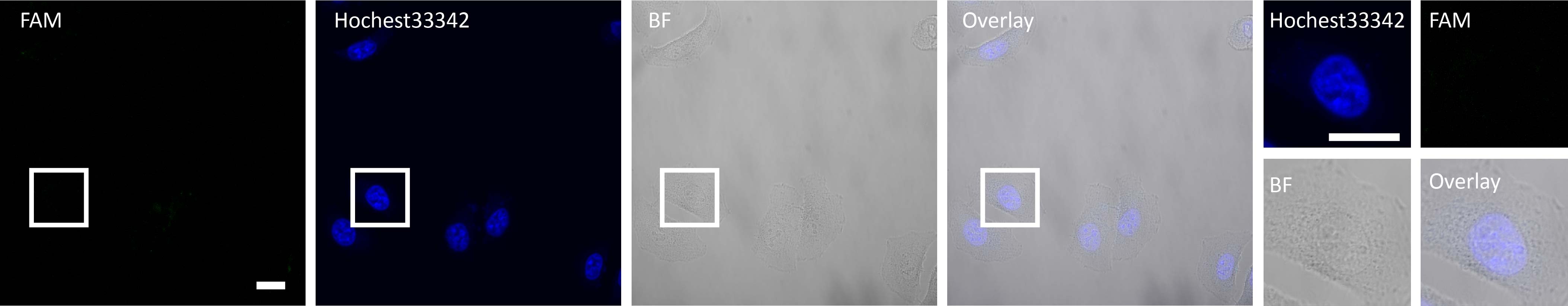
**

**Figure S15.** Control experiment using DNA-ND without liposomes. The confocal images did not show significant fluorescence, indicating little DNA entered cells. Scale bar: 20 μm.


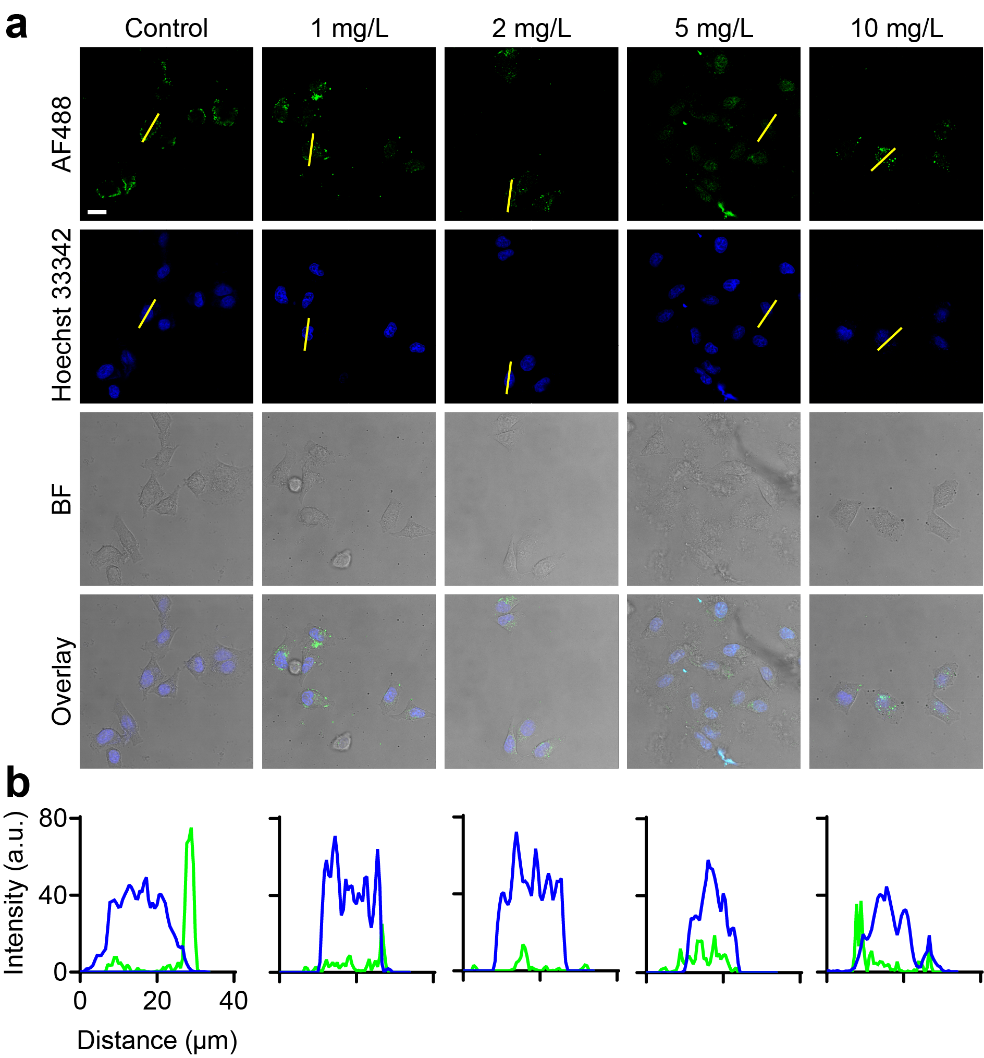


**Figure S16**. Optimization of NLS concentration. a, Confocal images of HeLa cells incubated with DNA-ND-Lipo/DNA-ND-Lipo*_n_* with 0 mg/L (control), 1 mg/L, 2 mg/L, 5 mg/L and 10 mg/L. AF 488: green fluorescence. Hoechst 33342: blue fluorescence. BF: bright field. Overlay: mixed fluorescence channel. Scale bar: 20 μm. b, Intensity profiles of interest in a as indicated by the yellow lines.

**
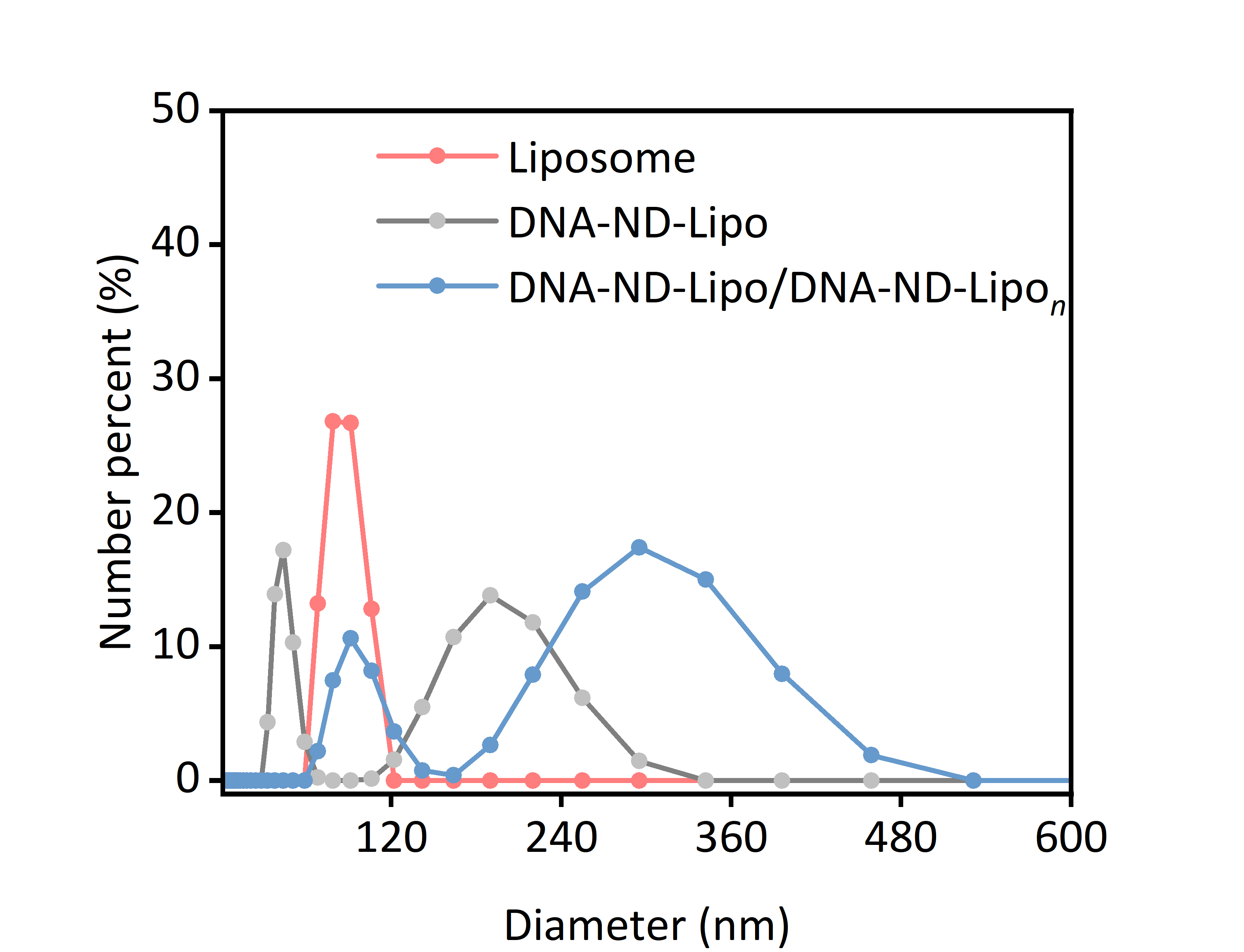
**

**Figure S17.** Dynamic light scattering (DLS) characterization for the modification processing of DNA-ND-Lipo and DNA-ND-Lipo/DNA-ND-Lipo*_n_*.

**
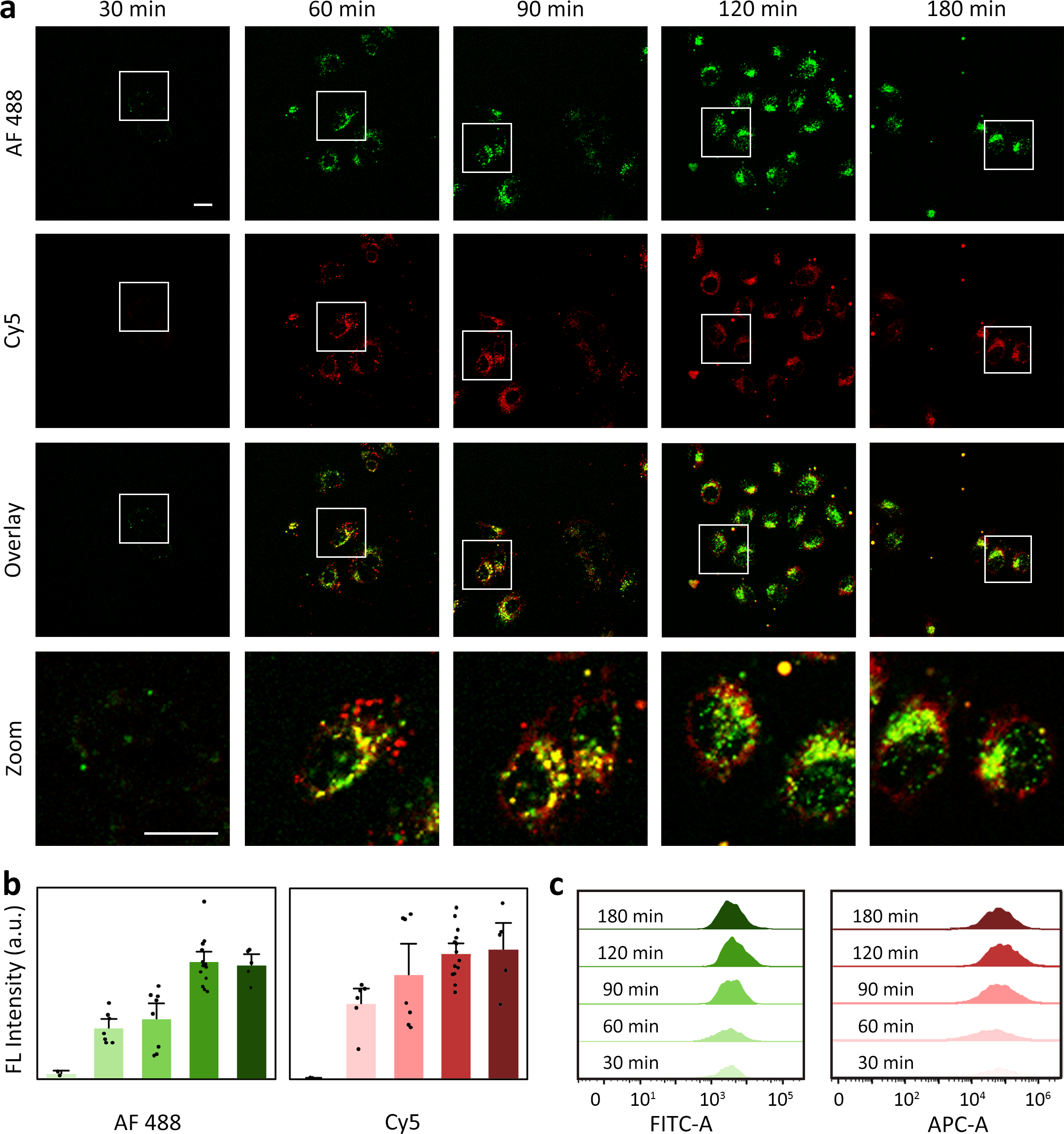
**

**Figure S18.** Optimization of incubation time. a, Confocal images of HeLa cells incubated with DNA-ND-Lipo/DNA-ND-Lipo*_n_* for 30, 60, 90, 120 and 180 min. AF 488: green fluorescence. Cy5: red fluorescence. Overlay: mixed fluorescence channel. Zoom: magnified images of single cell in mixed fluorescence channel. Scale bar: 20 μm. b, Fluorescent intensity corresponding to confocal fluorescence images in a. From left to right: 30 min to 180 min. c, Flow cytometric analysis of HeLa cells treated with DNA-ND-Lipo/DNA-ND-Lipo*_n_* for different times according to CLSM images.


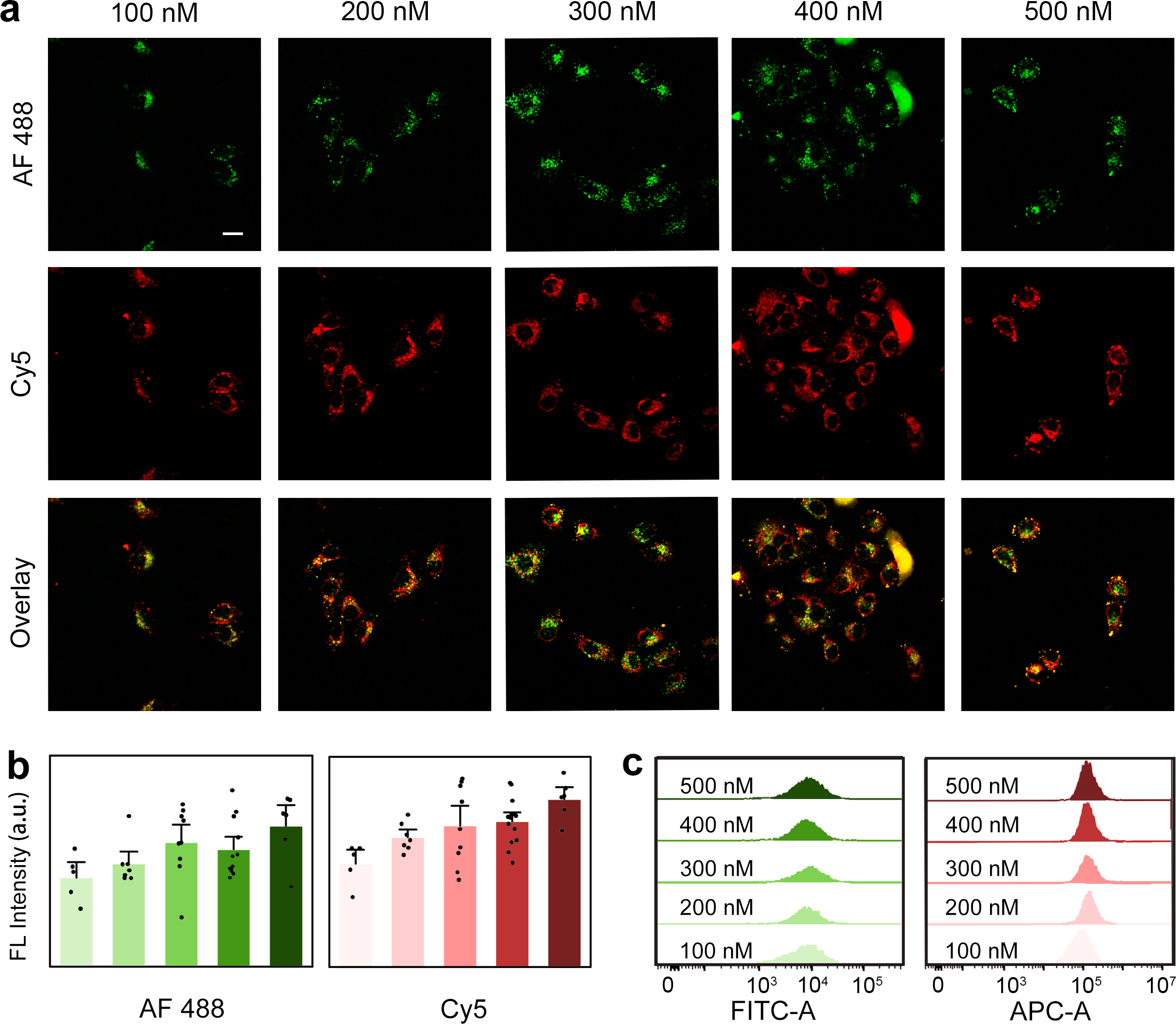


**Figure S19**. Optimization of dye concentration. a, Confocal images of HeLa cells incubated with DNA-ND-Lipo/DNA-ND-Lipo*_n_* at 100, 200, 300, 400 and 500 nM. AF 488: green fluorescence. Cy5: red fluorescence. Overlay: mixed fluorescence channel. Zoom: magnified images of a single cell in mixed fluorescence channel. Scale bar: 20 μm. b, Fluorescent intensity corresponding to confocal fluorescence images in a. From left to right: 100 to 500 nM. c, Flow cytometric analysis of HeLa cells treated with DNA-ND-Lipo/DNA-ND-Lipo*_n_* at different concentrations according to CLSM images.

**
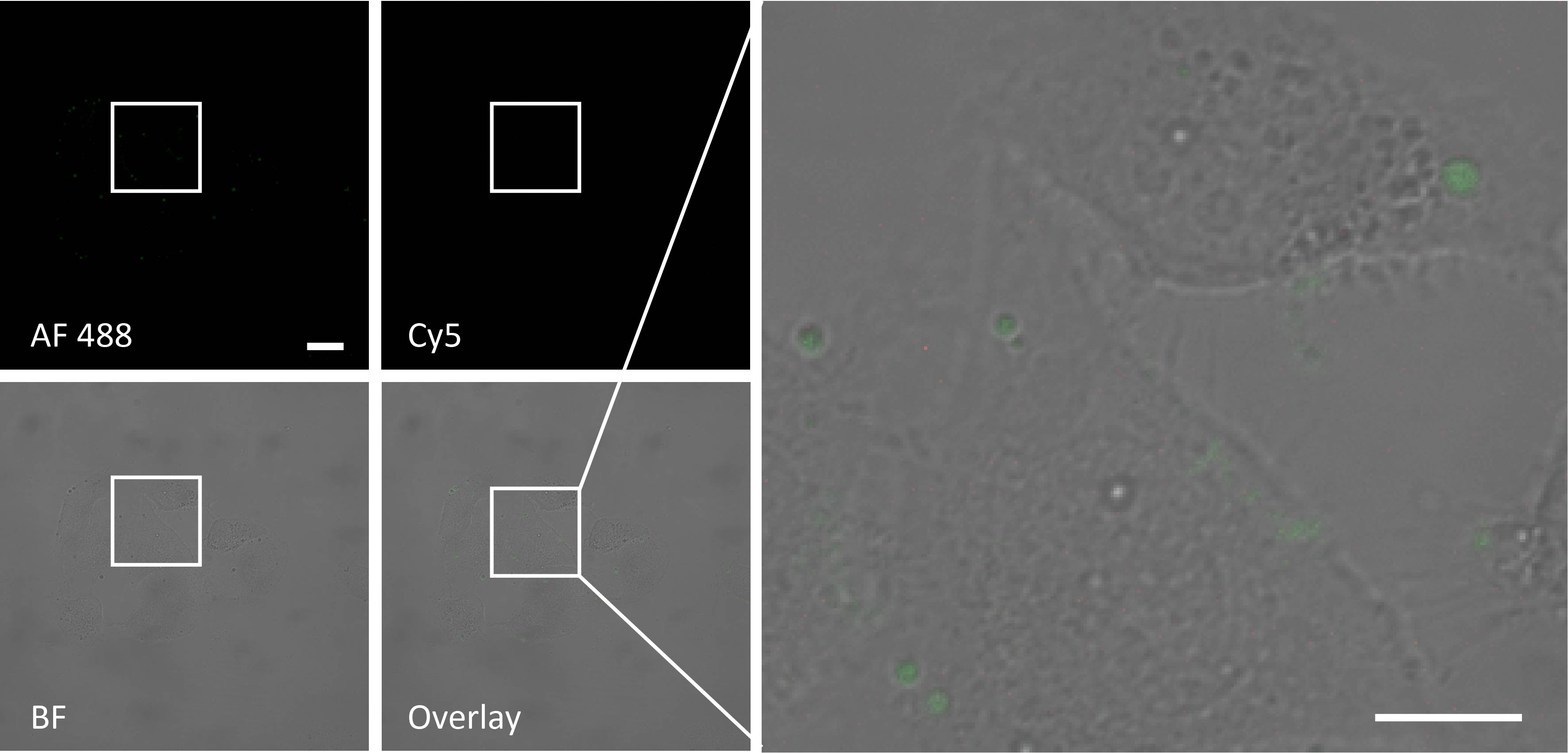
**

**Figure S20**. Negative control using mismatched DNA-ND-Lipo/DNA-ND-Lipo*_n_*. Confocal images of HeLa cells incubated with mismatched DNA-ND-Lipo/DNA-ND-Lipo_n_. Scale bar: 20 μm.

**
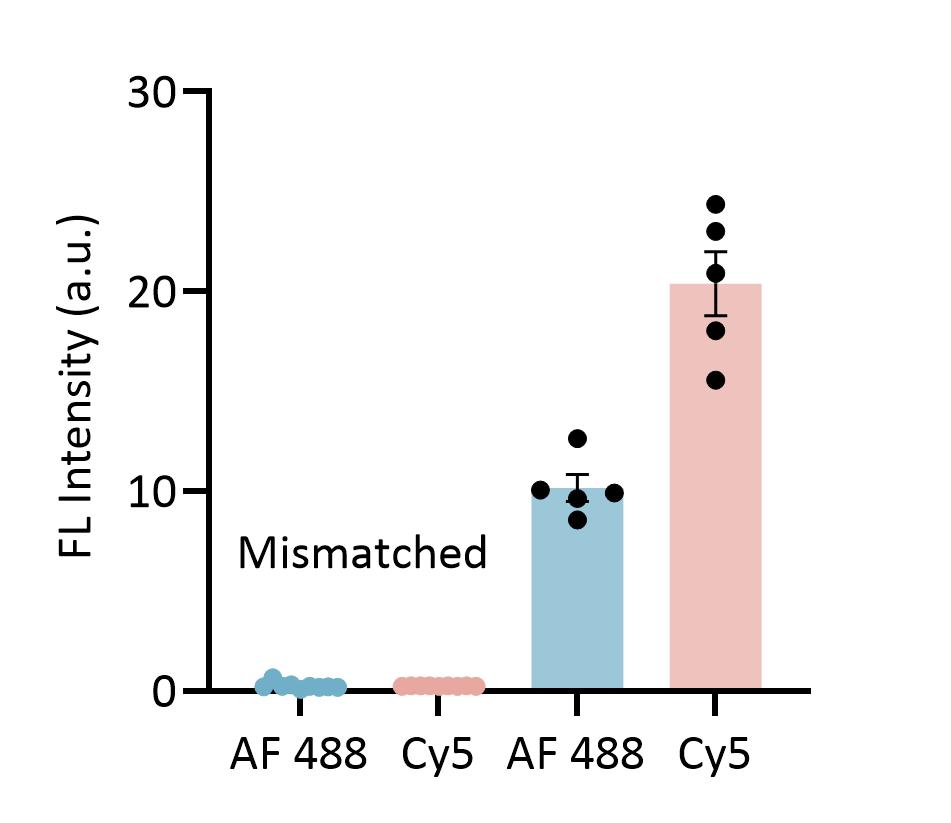
**

**Figure S21.** Fluorescence intensity of control and experimental groups. The fluorescence intensity corresponding to confocal fluorescence images in Fig. 1e and Figure S20.


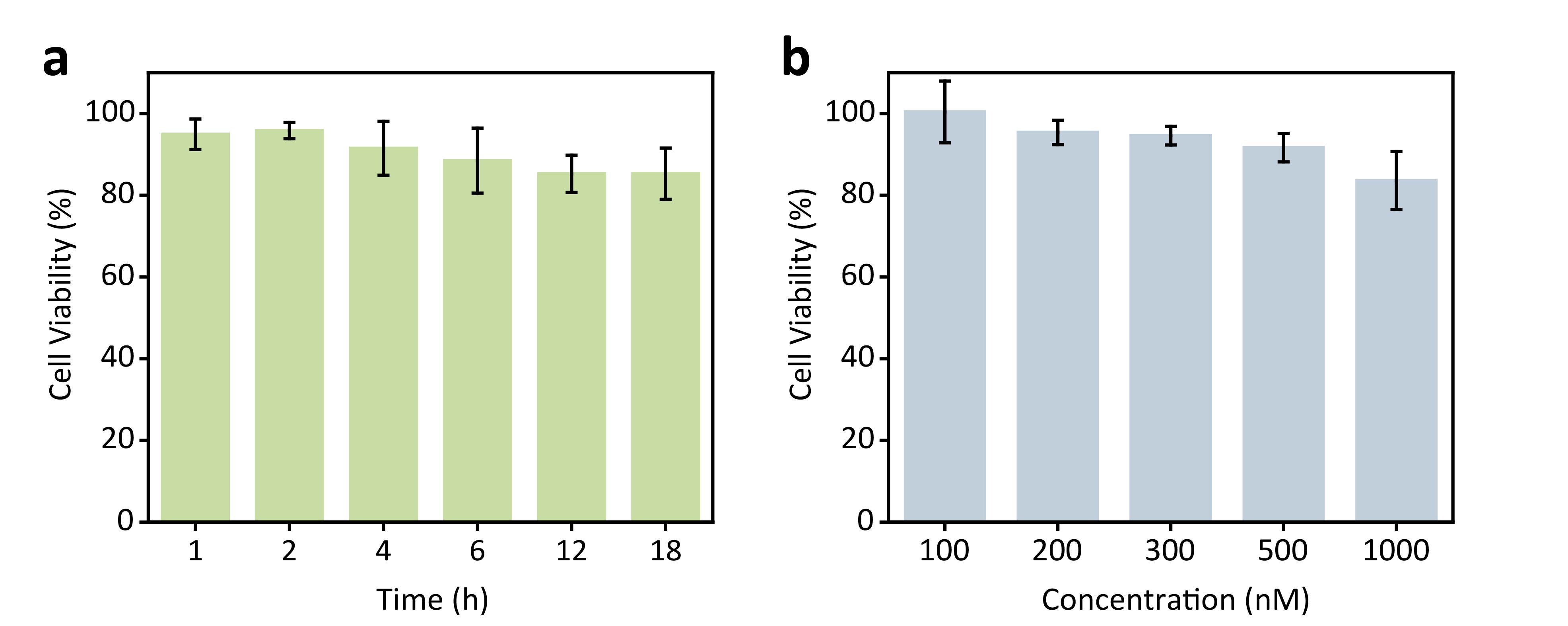


**Figure S22.** Cytotoxicity of DNA-ND-Lipo. a, Cell viability (%) of HeLa cells incubated with DNA-ND-Lipo after different times. b, Cell viability (%) of HeLa cells incubated with DNA-ND-Lipo of different DNA-ND concentrations for 3 h. The cell viability was tested by CCK-8 assay, using HeLa cells (100 μL, 1.0×10^5^ mL^-1^) treated with 10 μL probe solution and incubated for different times at 37 °C.

The DNA-ND-Lipo was not found apparent toxicity on HeLa cells after 24 h treatment. When the concentration of DNA-ND reached 1 μM, HeLa cells could still maintain a high level of activity.

**
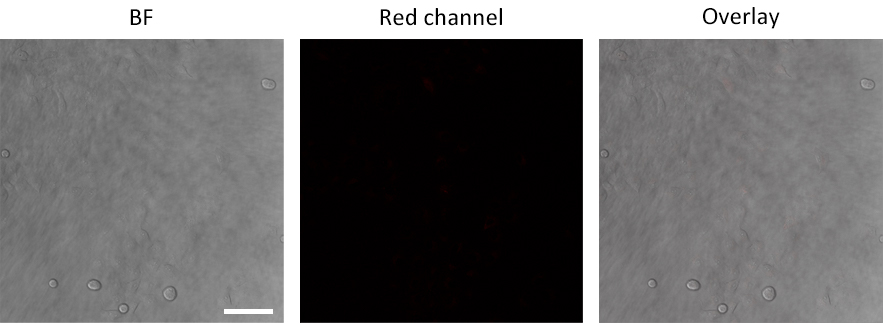
**

**Figure S23.** Cytotoxicity of DNA-ND-Lipo in HeLa cells by confocal fluorescent images. HeLa cells were incubated with 0.04% trypan blue for 5 minutes. Scale bar: 100 μm.


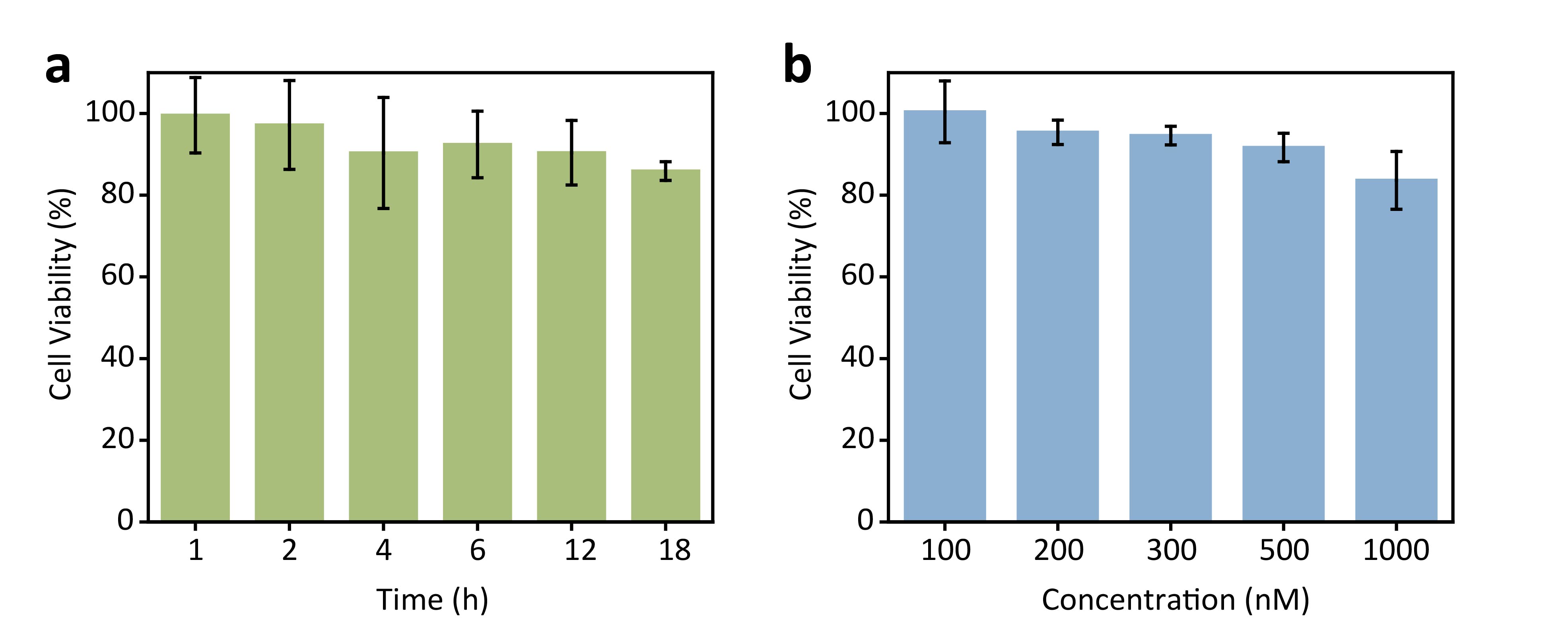


**Figure S24.** Cytotoxicity of DNA-ND-Lipo/DNA-ND-Lipo*_n_*. a, Cell viability (%) of HeLa cells incubated with DNA-ND-Lipo/DNA-ND-Lipo*_n_* after different times. b, Cell viability (%) of HeLa cells incubated with DNA-ND-Lipo/DNA-ND-Lipo*_n_* of different DNA-ND concentration for 3 h. The cell viability was tested by CCK-8 assay, using HeLa cells (100 μL, 1.0×10^5^ mL^-1^) treated with 10 μL probe solution and incubated for different times at 37 °C.

The DNA-ND-Lipo/DNA-ND-Lipo*_n_* was not found apparent toxicity on HeLa cells after 24 h treatment. When the concentration of DNA-ND reached 1 μM, HeLa cells could still maintain a high level of activity.

**
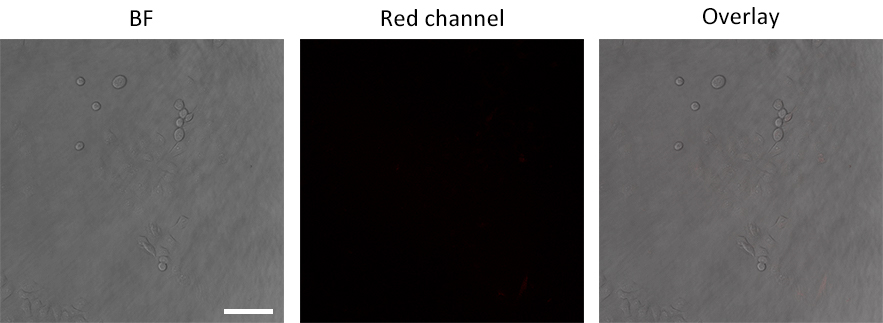
**

**Figure S25.** Cytotoxicity of DNA-ND-Lipo/DNA-ND-Lipo*_n_* in HeLa cells by confocal fluorescent images. HeLa cells were incubated with 0.04% trypan blue for 5 minutes. Scale bar: 100 μm.


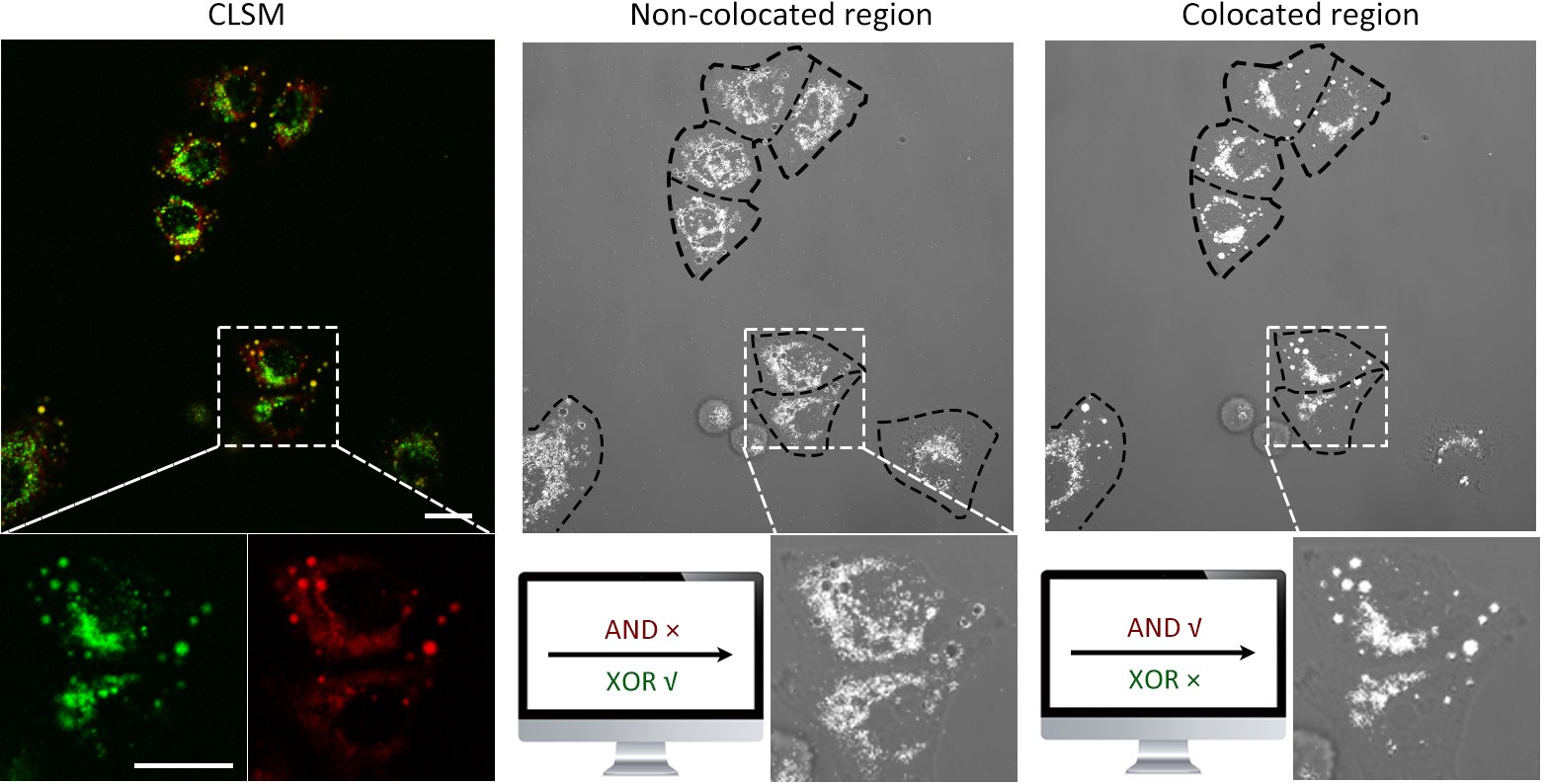


**Figure S26**. Schematic diagram of fluorescence distribution analysis using logic gates. After applying “XOR” and “AND” logic operation, fluorescence colocalization regions and non-colocalization regions can be displayed directly. Scale bar: 10 μm.

**

**

**Figure S27**. Cytotoxicity of BIBR1532. The cell viability was tested by CCK-8 assay, using HeLa cells (100 μL, 1.0×10^5^ mL^-1^) treated with BIBR1532 of different concentrations at 37 °C after 24 h. The control group was treated with 20% DMSO.

**
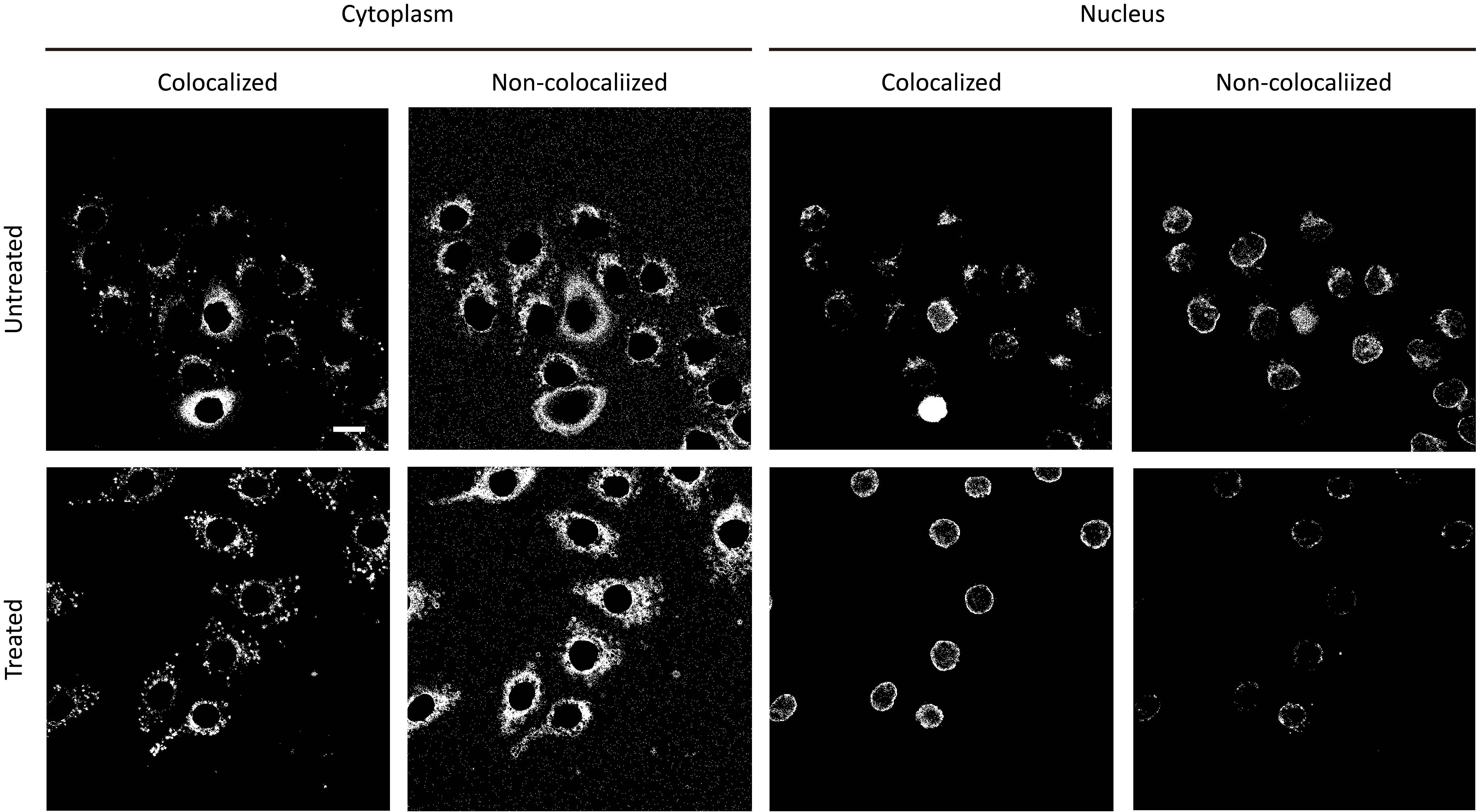
**

**Figure S28.** Logic output of HeLa cells with and without BIBR1532 treatment. Scale bar: 20 μm.


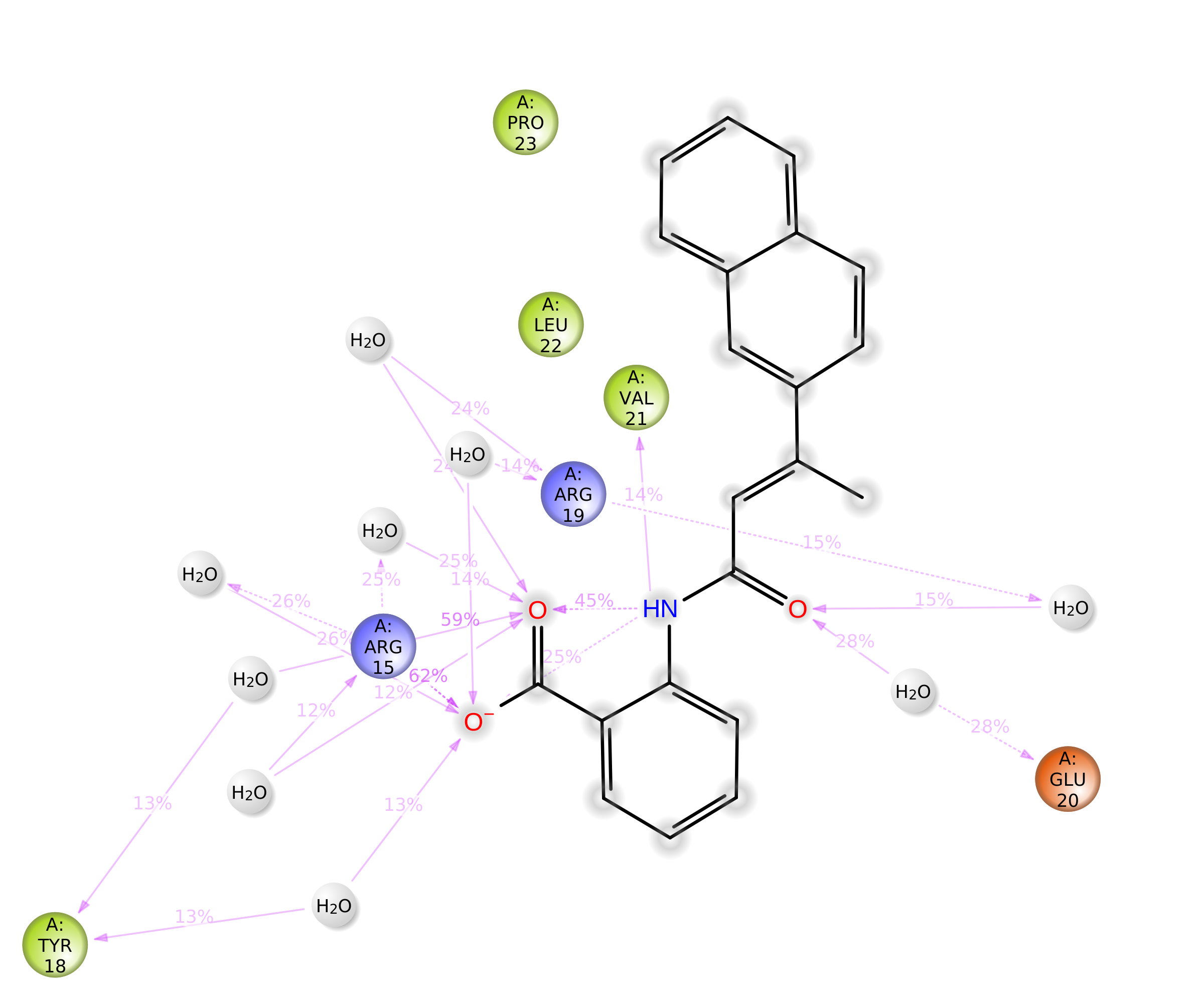


**Figure S29**. Detailed ligand atom interactions with the TERT residues. Interactions that occur more than 10.0% of the simulation time in the selected trajectory (0.00 through 100.00 nsec).

**
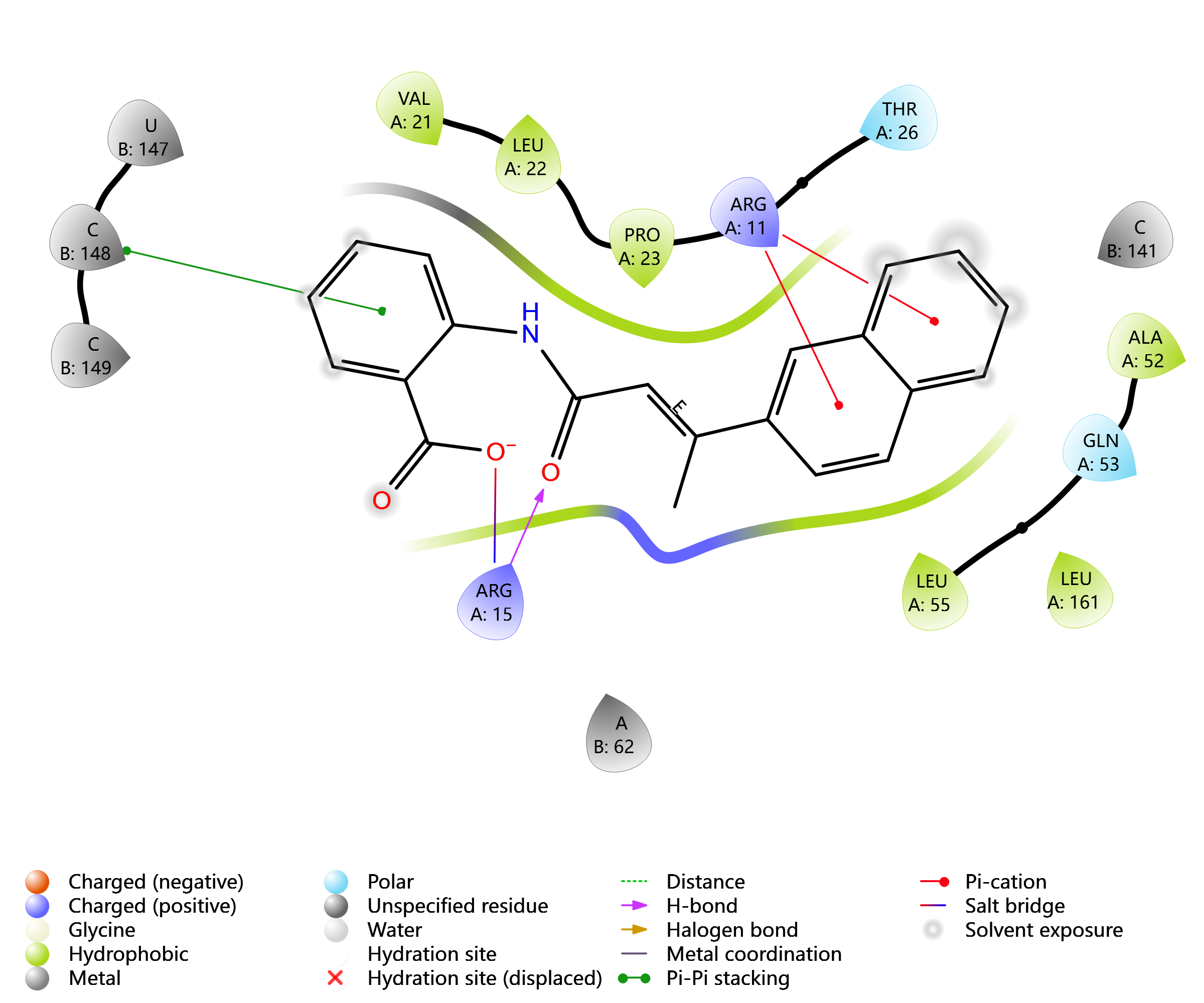
**

**Figure S30**. Template/pseudoknot of hTR and its interaction with TERT TEN domains in state 1. BIBR1532 binds to the active pocket of the TERT, forming two π cation bonds with the protein amino acid residue ARG11, a hydrogen bond and a salt bridge with ARG15, and a π-π bond with the 148th C base of hTR.


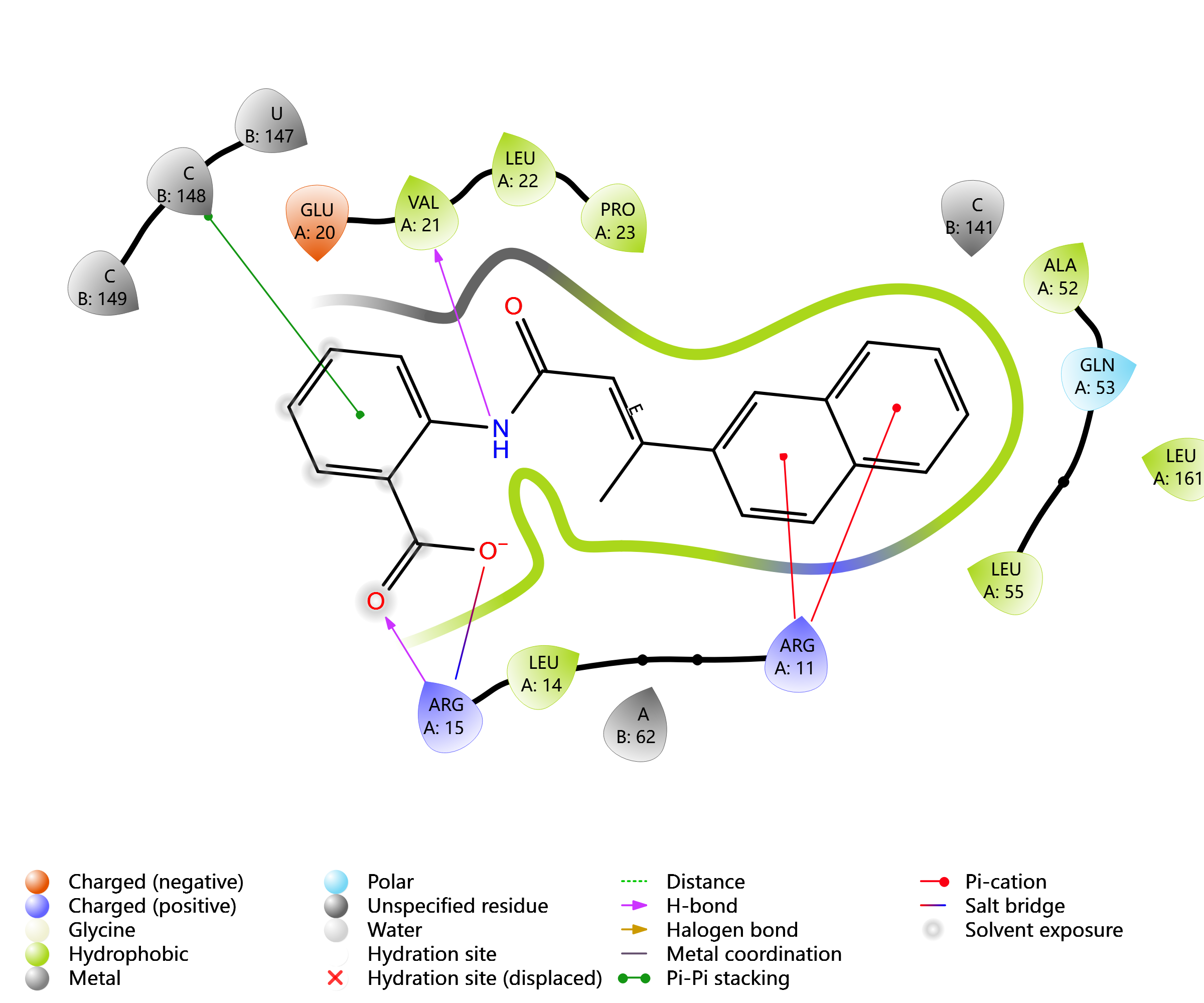


**Figure S31**. Template/pseudoknot of hTR and its interaction with TERT TEN domains in state 2. This figure shows the results of kinetic simulation in 0 ns. BIBR1532 binds in the active pocket of TERT, forms two π cation bonds with the protein amino acid residue ARG11, a hydrogen bond and a salt bridge with ARG15, a hydrogen bond with VAL21, and a π-π bond with the 148th C base of hTR.

**
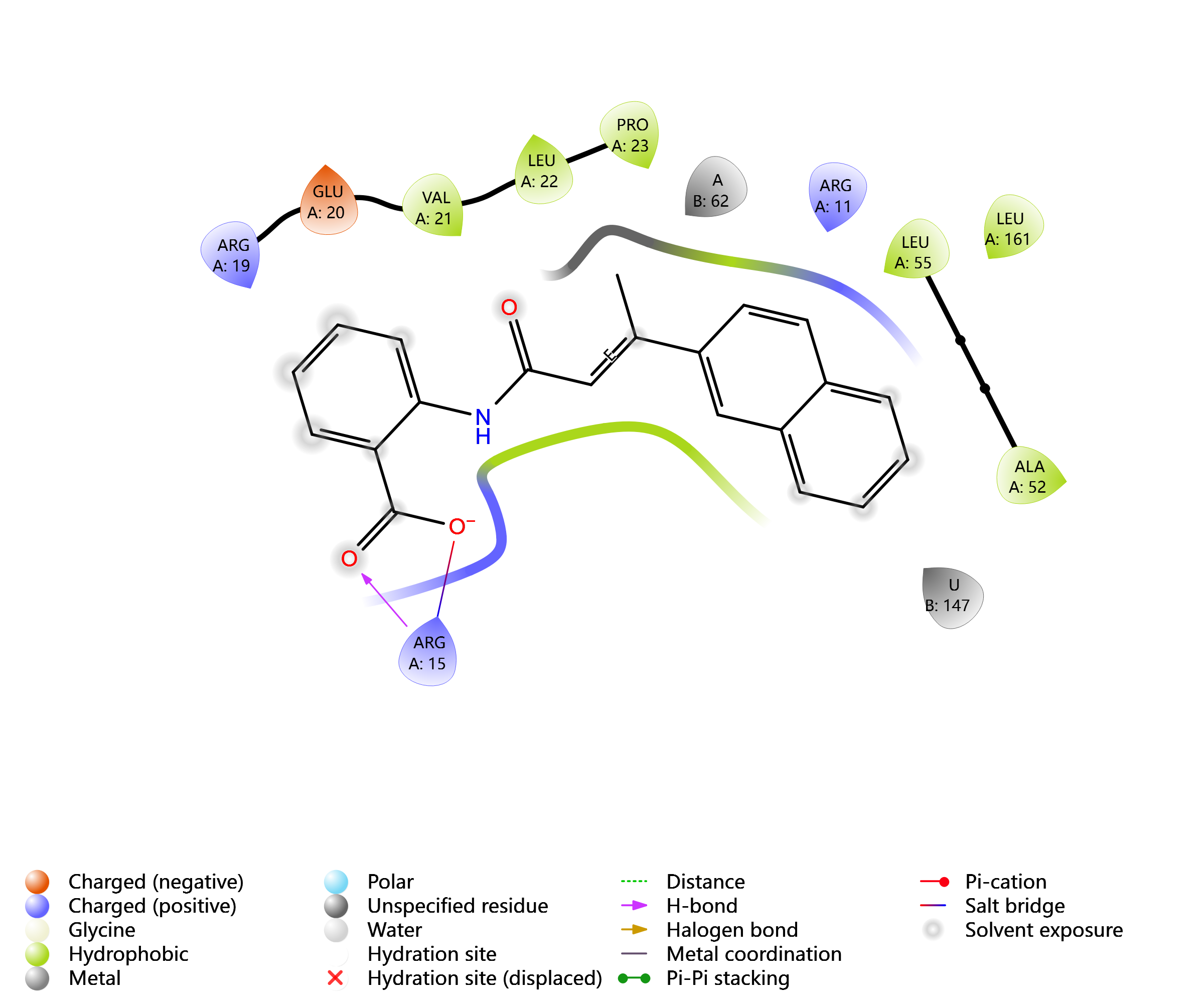
**

**Figure S32**. Template/pseudoknot of hTR and its interaction with TERT TEN domains in state 3. This figure shows the results of kinetic simulation for 15 ns, in which an allosteric reaction occurs, and the binding of the BIBR1532 to TERT changes greatly, and is no longer in a stable state, only forming a hydrogen bond and a salt bridge with the protein amino acid residue ARG15, and the rest of the binding sites have disappeared.

**
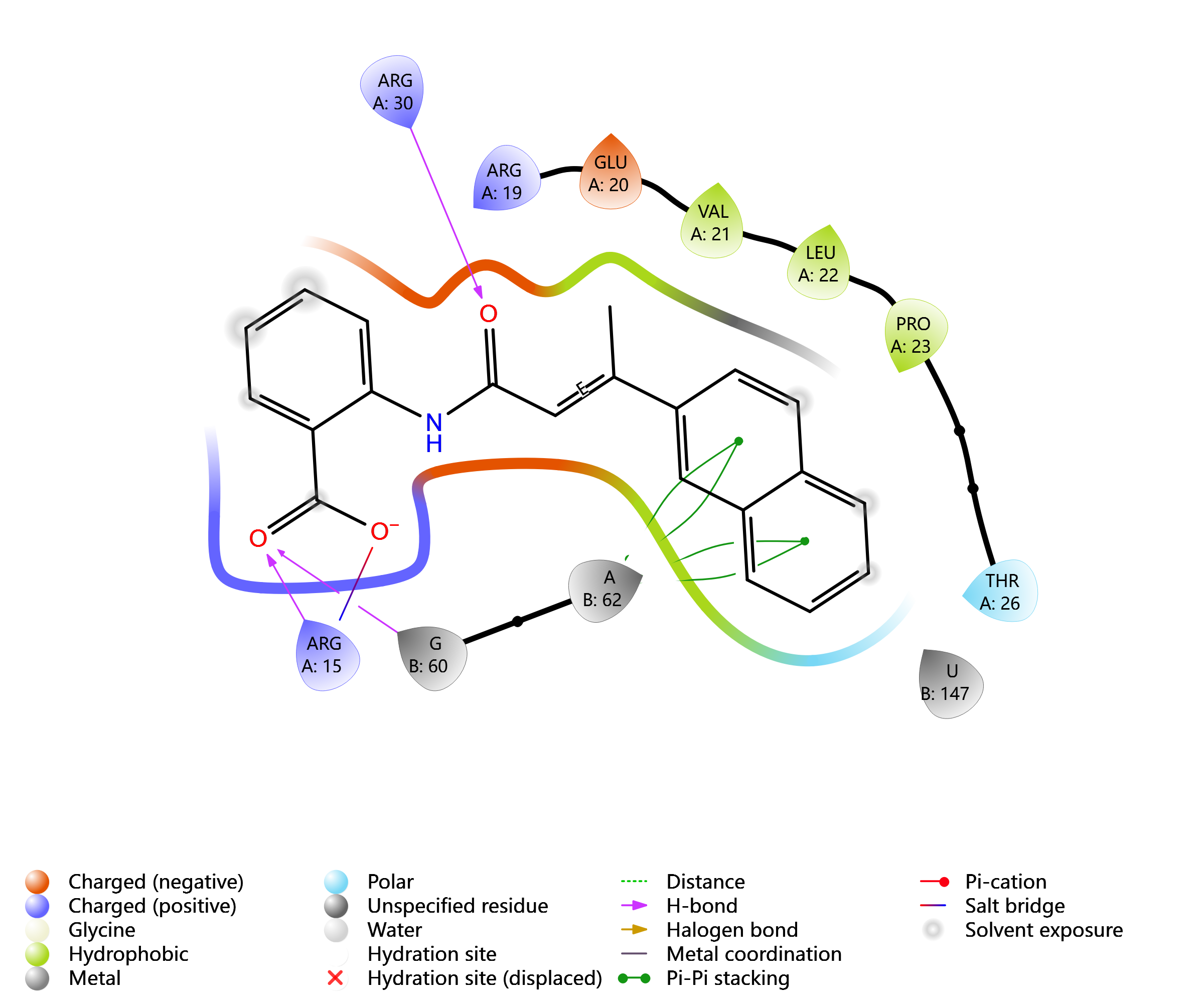
**

**Figure S33**. Template/pseudoknot of hTR and its interaction with TERT TEN domains in state 4. This figure shows the results of kinetic simulation for 100 ns, at which the BIBR1532 re-enters a new stable state by binding to TERT and BIBR1532 forms a hydrogen bond and a salt bridge with the protein amino acid residue ARG15, a hydrogen bond with ARG30, a hydrogen bond with the 60th G base of hTR, and four π-π bonds with the 62nd A base.


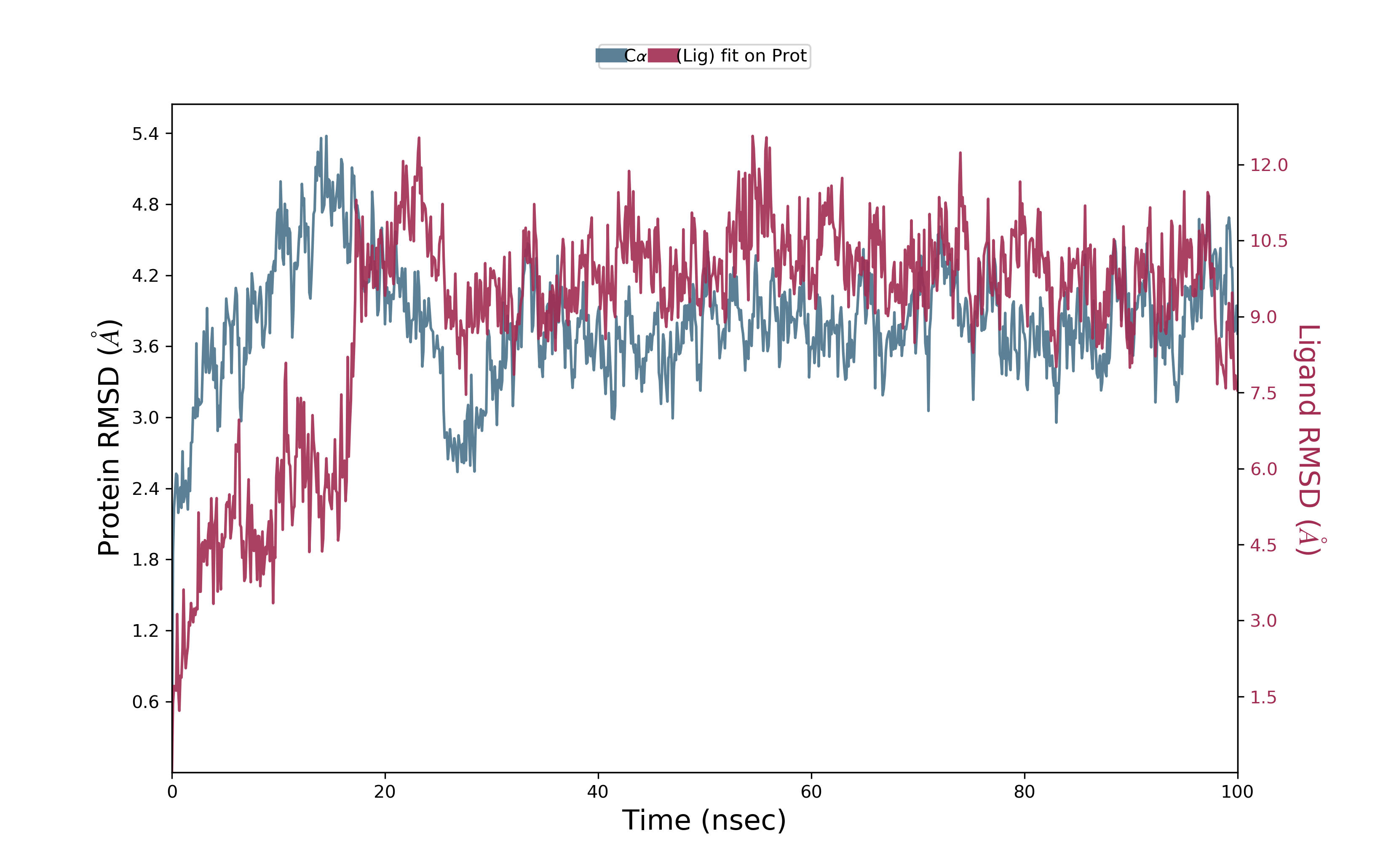


**Figure S34**. RMSD results for TERT and BIBR1532. The BIBR1532 (red) was simulated with TERT (blue) for 100 ns MD and its molecular dynamics trajectories were analyzed. The figure shows the conformational stability of RMSD over simulated time, in which small fluctuations indicate that all complexes have obtained stable conformation, and the results show that the BIBR1532 and TERT are relatively stable after 30 ns, and the system is in equilibrium.

The RMSD for frame x could be calculated as following:

${RMSD}_{x}=\sqrt{\frac{1}{N}\sum_{i=1}^{N} {\left( r_{i}^{'}\left( t_{x} \right) \right)-r_{i}((t_{ref}))}^{2}}$ equation (1)

Where *N* was the number of atoms in the atom selection, *t_ref_* was the reference time (typically, the first frame was used as the reference and regarded as t = 0)*, r*' was the position of the selected atoms in frame *x* after superimposing on the reference frame, and frame x was recorded at time *t_x_*. This procedure was repeated for each frame in the simulation trajectory. RMSD results for TERT (blue) and BIBR1532 (red) demonstrated the conformational stability of RMSD over simulated time, in which small fluctuations indicated that all complexes obtained stable conformation.


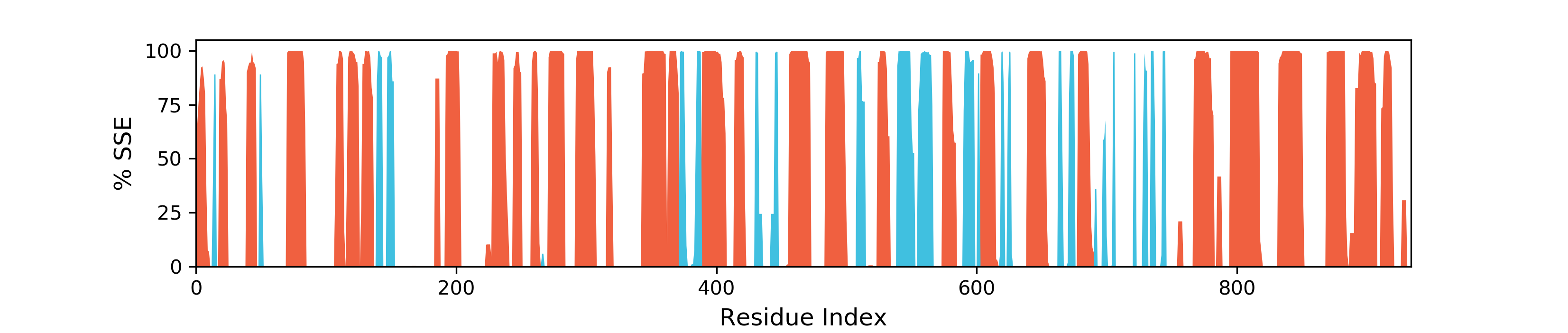


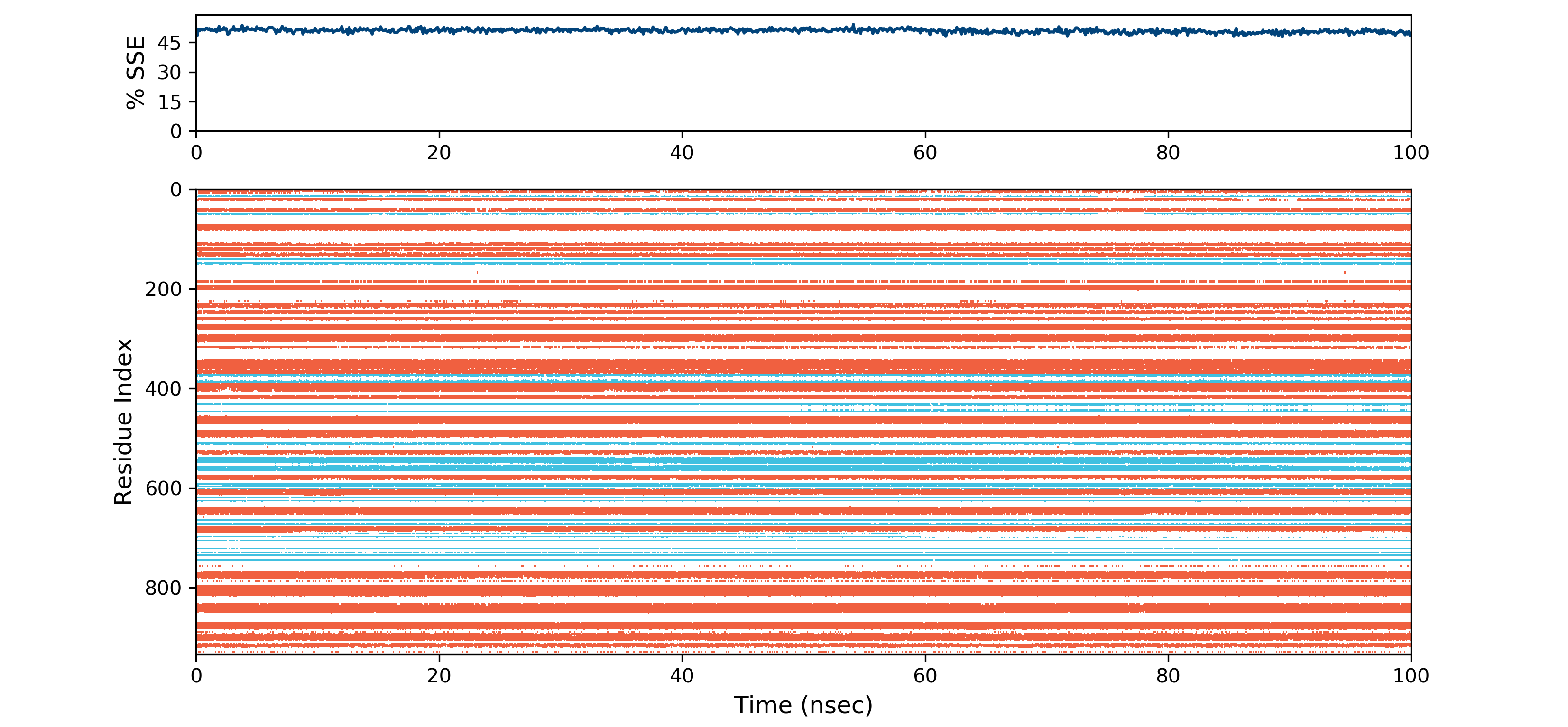


**Figure S35**. Protein decondary dtructure. The plot above reports protein secondary structure elements (SSE) distribution by residue index throughout the protein structure. The plot below summarizes the SSE composition for each trajectory frame over the course of the simulation, and the plot at the bottom monitors each residue and its SSE assignment over time.


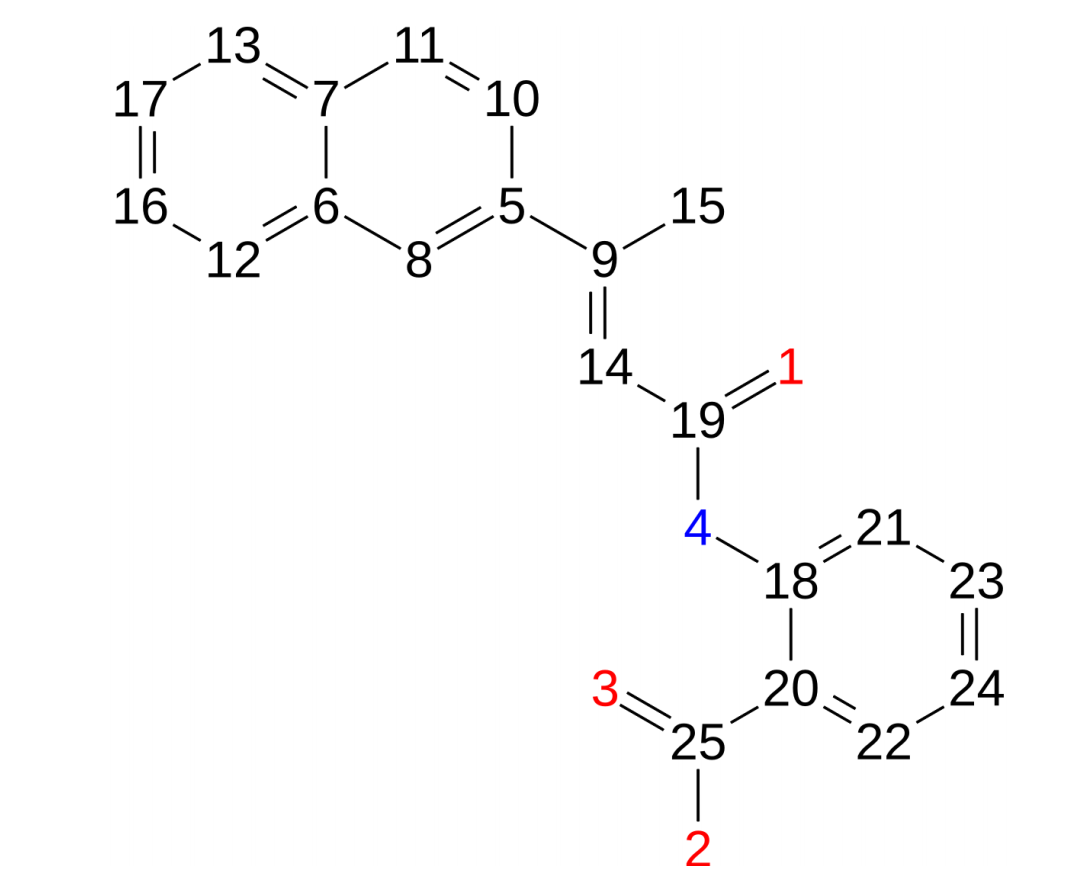

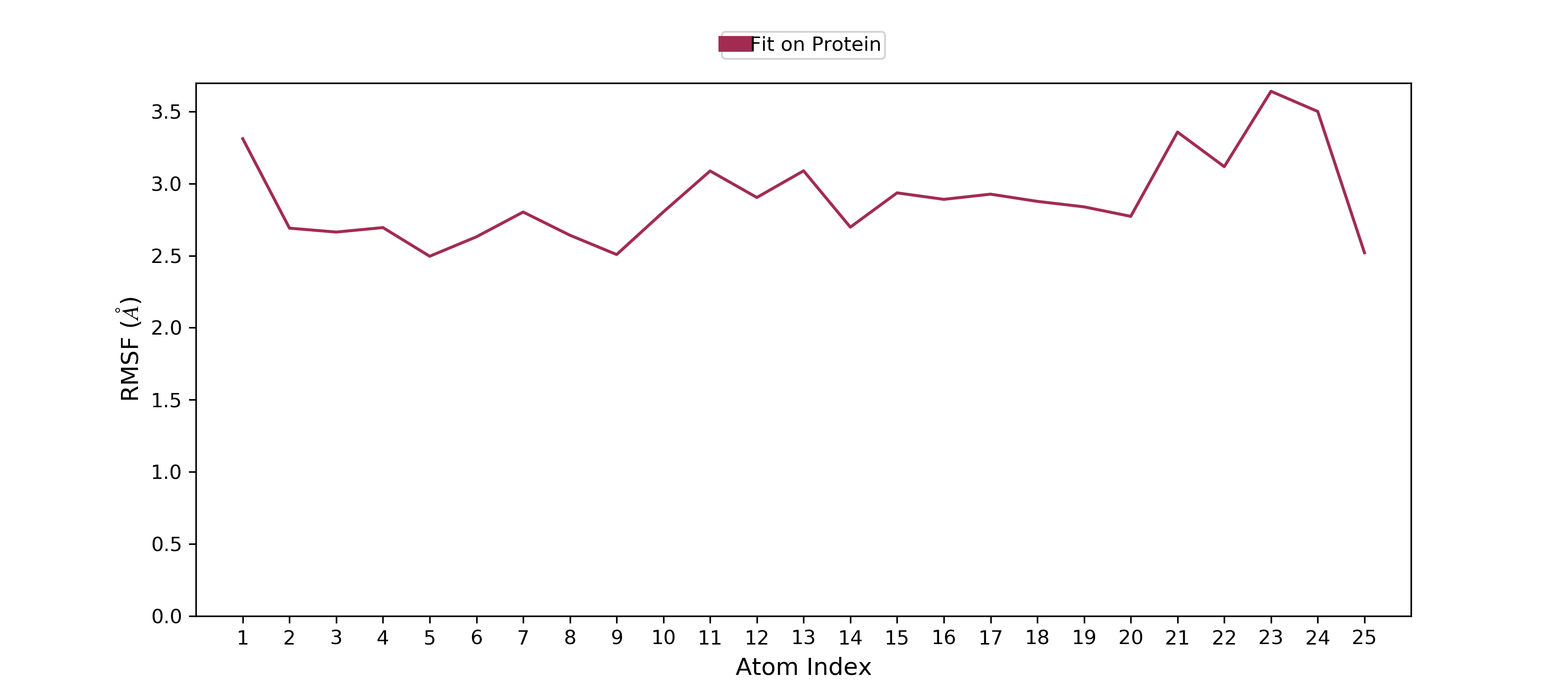


**Figure S36.** Ligand RMSF.

The Ligand Root Mean Square Fluctuation (L-RMSF) is useful for characterizing changes in the ligand atom positions. The RMSF for residue *i* could be calculated as following:

${RMSF}_{i}=\sqrt{\frac{1}{T}\sum_{t=1}^{T} <{\left( r_{i}^{'}\left( t \right) \right)-r_{i}((t_{ref}))}^{2}>}$ equation (2)

Where *t* was the trajectory time, *t*_ref_ was the reference time, *r_i_* was the position of residue *I*, and *r'* was the position of atoms in residue *i* after superposition on the reference. The angle brackets indicated that average square distance was taken over the selected atoms in the residue. For the RMSF plots, the peaks indicated dramatic fluctuations in the site of a protein.

**
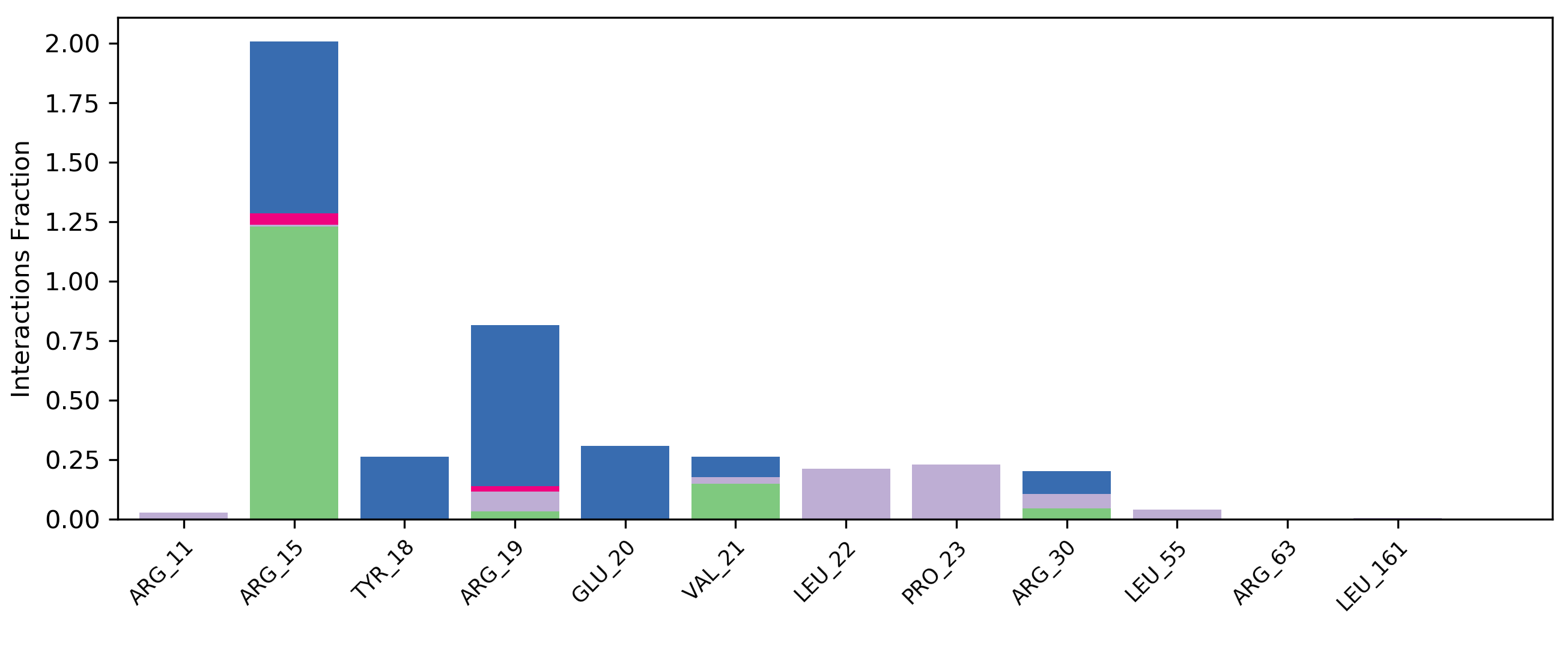
**

**Figure S37**. Contribution of amino acids at binding sites to TERT binding. Protein-ligand interactions can be monitored throughout the simulation, and their interactions can be divided into four types: hydrogen bonding, hydrophobicity, ion bridges, and water bridges. As shown in the figure above, the amino acids that play an important role in the binding of BIBR1532 to TERT are mainly ARG15 and ARG19, and their interactions are mainly water bridges and hydrogen bonds.

**
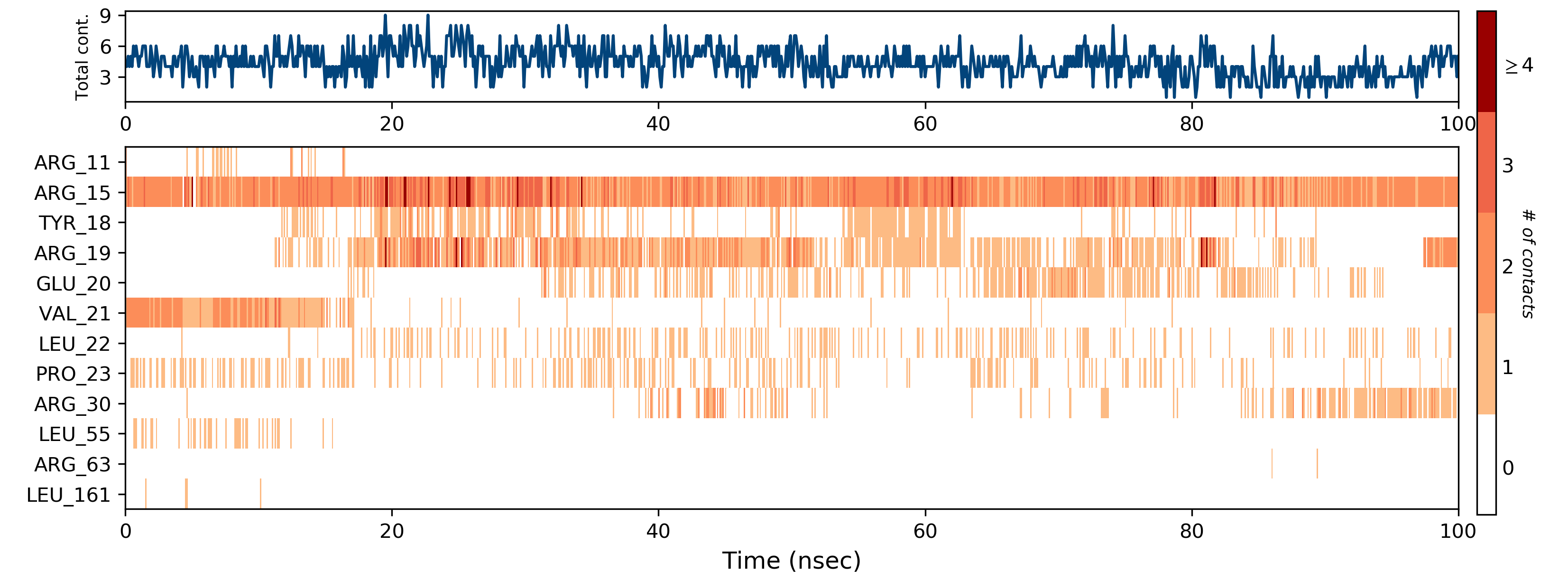
**

**Figure S38**. BIBR1532 interactions with specific amino acids of TERT over time. The amino acid residues ARG15, TYR18, ARG19, GLU20, VAL21, LEU22, PRO23, ARG30 have multiple contacts with the ligand (indicated by a darker orange color).


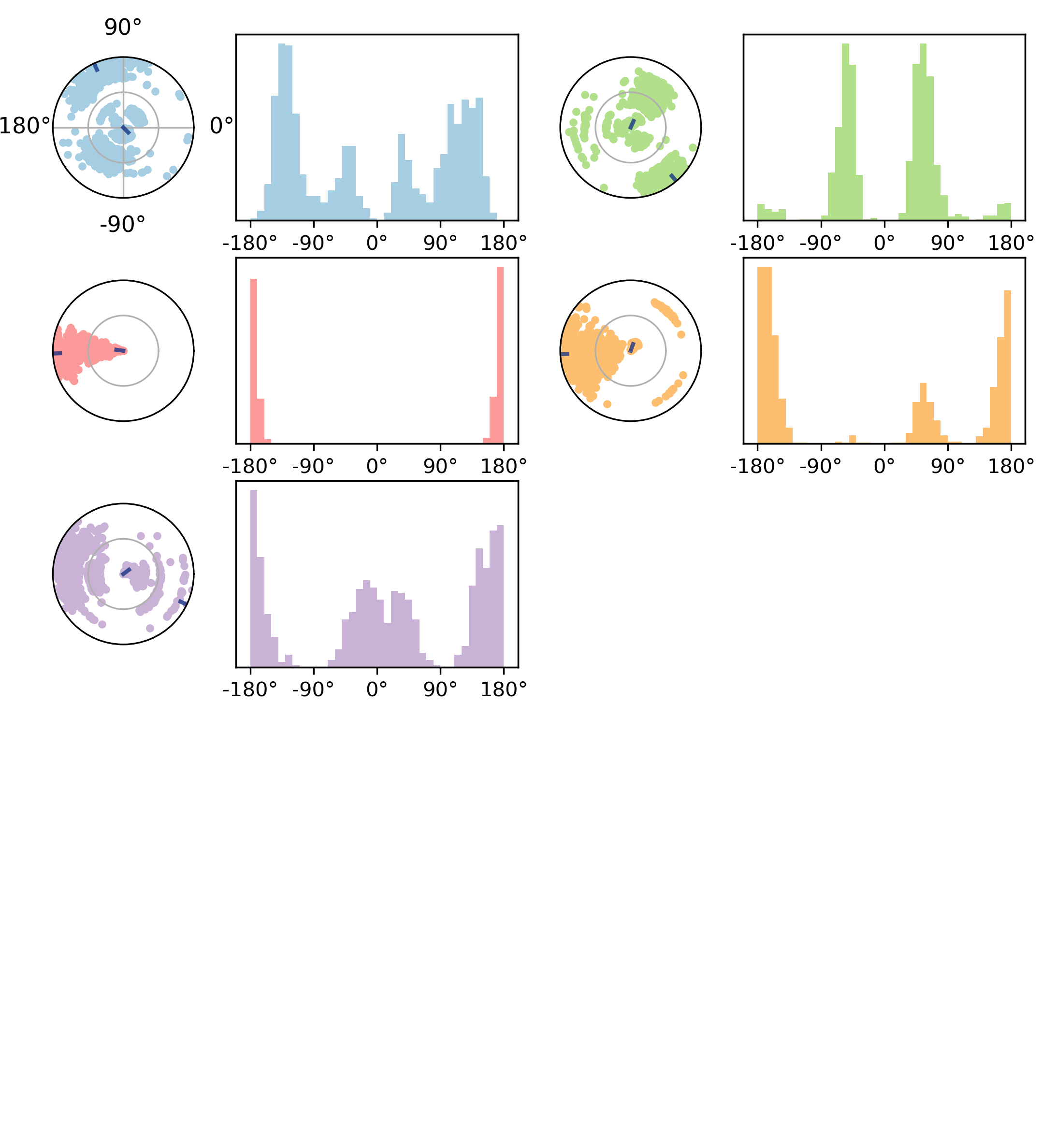
**Figure S39**. Ligand torsion diagram and dial (or radial) diagram. The ligand torsions plot summarizes the conformational evolution of every rotatable bond (RB) in the ligand throughout the simulation trajectory (0.00 through 100.00 nsec). The top panel shows the 2d schematic of a ligand with color-coded rotatable bonds. Each rotatable bond torsion is accompanied by a dial plot and bar plots of the same color. Dial (or radial) plots describe the conformation of the torsion throughout the course of the simulation. The beginning of the simulation is in the center of the radial plot and the time evolution is plotted radially outwards. The bar plots summarize the data on the dial plots, by showing the probability density of the torsion. The values of the potential are on the left Y-axis of the chart, and are expressed in kcal/mol. Looking at the histogram and torsion potential relationships may give insights into the conformational strain the ligand undergoes to maintain a protein-bound conformation.


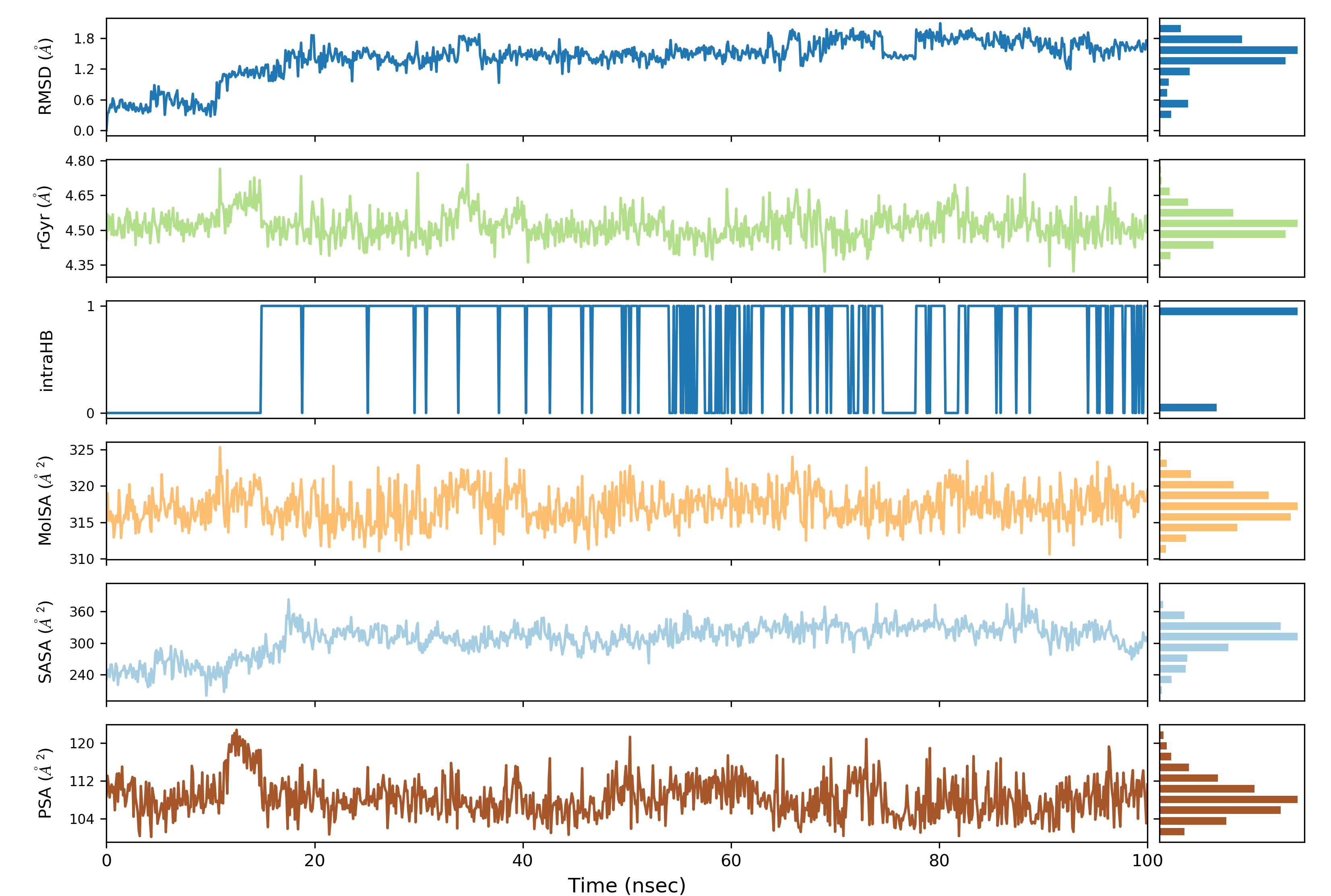


**Figure S40.** Ligand properties. Ligand RMSD: Root mean square deviation of a ligand with respect to the reference conformation (typically the first frame is used as the reference and it is regarded as time t=0). Radius of Gyration (rGyr): Measures the ‘extendedness’ of a ligand, and is equivalent to its principal moment of inertia. Intramolecular Hydrogen Bonds (intraHB): Number of internal hydrogen bonds (HB) within a ligand molecule. Molecular Surface Area (MolSA): Molecular surface calculation with 1.4 Å probe radius. This value is equivalent to a van der Waals surface area. Solvent Accessible Surface Area (SASA): Surface area of a molecule accessible by a water molecule. Polar Surface Area (PSA): Solvent accessible surface area in a molecule contributed only by oxygen and nitrogen atoms.

**

**

**Figure S41.** Cytotoxicity of Sinefungin. The cell viability was tested by CCK-8 assay, using HeLa cells (100 μL, 1.0×10^5^ mL^-1^) treated with 50 μM Sinefungin for different times at 37 °C.


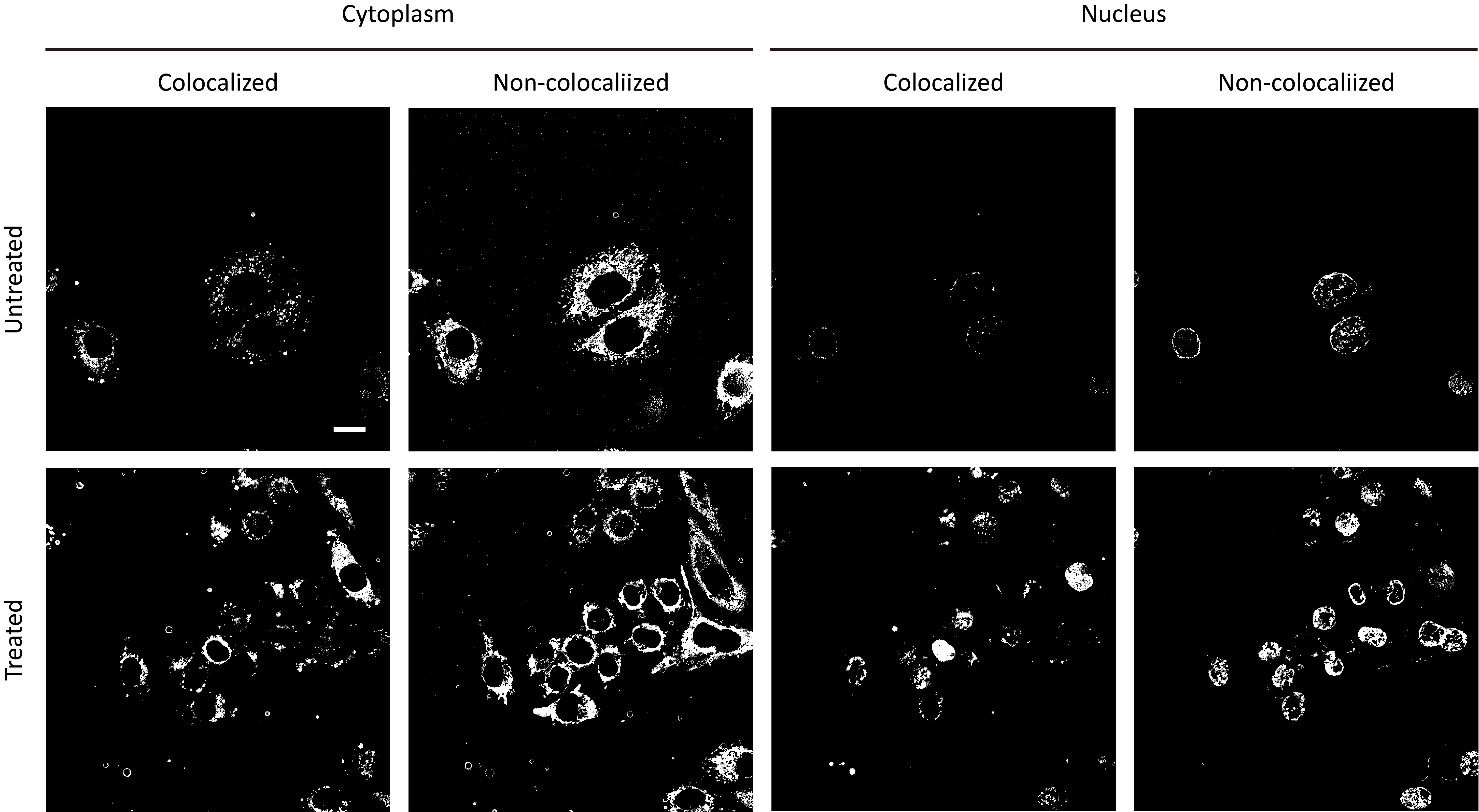


**Figure S42.** Logic output images of HeLa cells with and without Sinefungin treatment. Scale bar: 20 μm.

**Table S1.** DNA sequences.


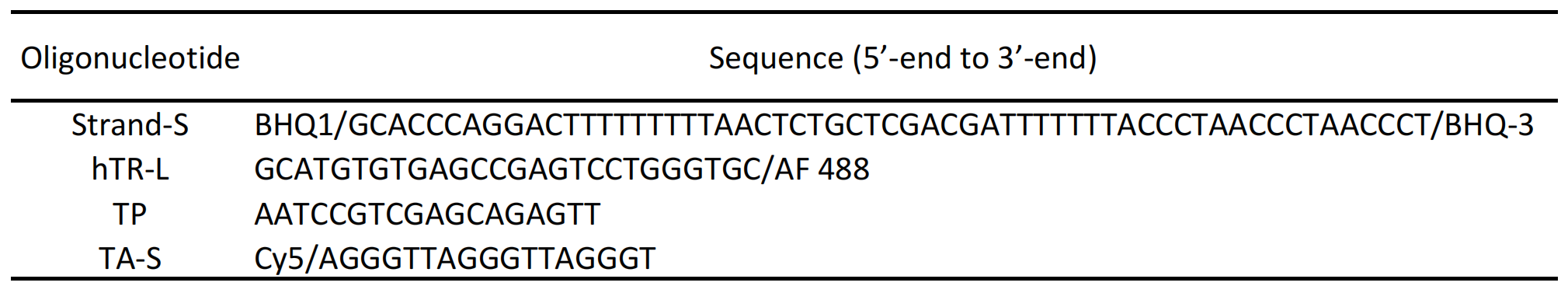


hTR-L was applied for the detection of hTR, which was an integral part of Part 1 in DNA-ND. TP was the telomerase primer strand and formed Part 2 with a telomerase activated signal strand TA-S. Strand-S was the substrate strand.

**Table S2.** DNA sequences for gel electrophoresis.


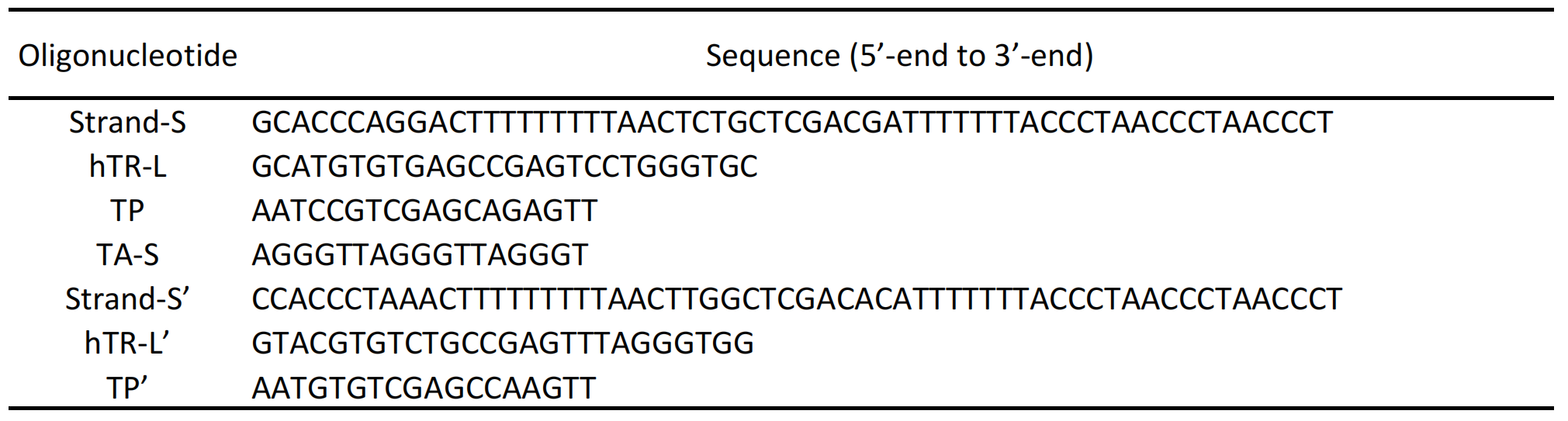


hTR-L was applied for the detection of hTR, which was an integral part of Part 1 in DNA-ND. TP was the telomerase primer strand and formed Part 2 with a telomerase activated signal strand TA-S. Strand-S was the substrate strand. These DNA sequences were used for verifying the specification of DNA-ND by gel electrophoresis in Fig.1d and Figure S10.

**Table S3.** Mismatched DNA sequences.


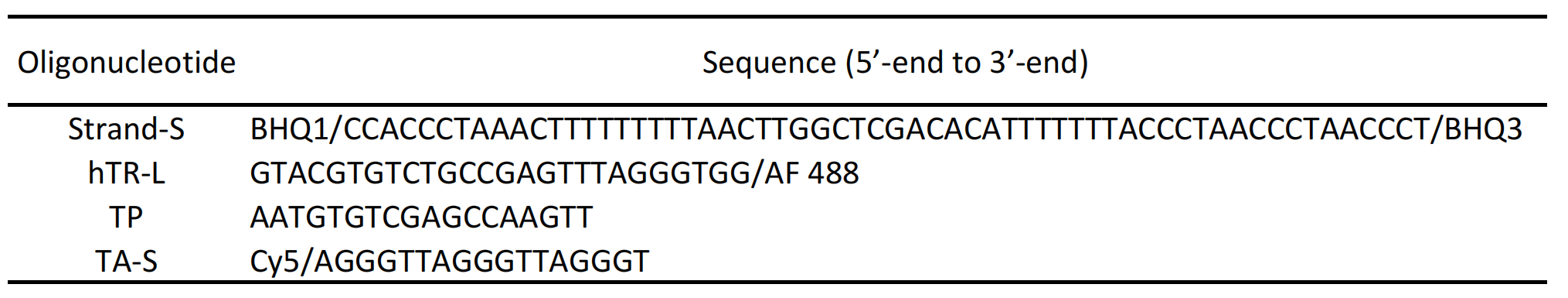


hTR-L was applied for the detection of hTR, which was an integral part of Part 1 in DNA-ND. TP was the telomerase primer strand and formed Part 2 with a telomerase activated signal strand TA-S. Strand-S was the substrate strand. These DNA sequences were used for verifying the specification of DNA-ND by FL spectrum and CLSM in Figures S8 and 20.

**Table S4.** Experimental parameters in CLSM experiments.


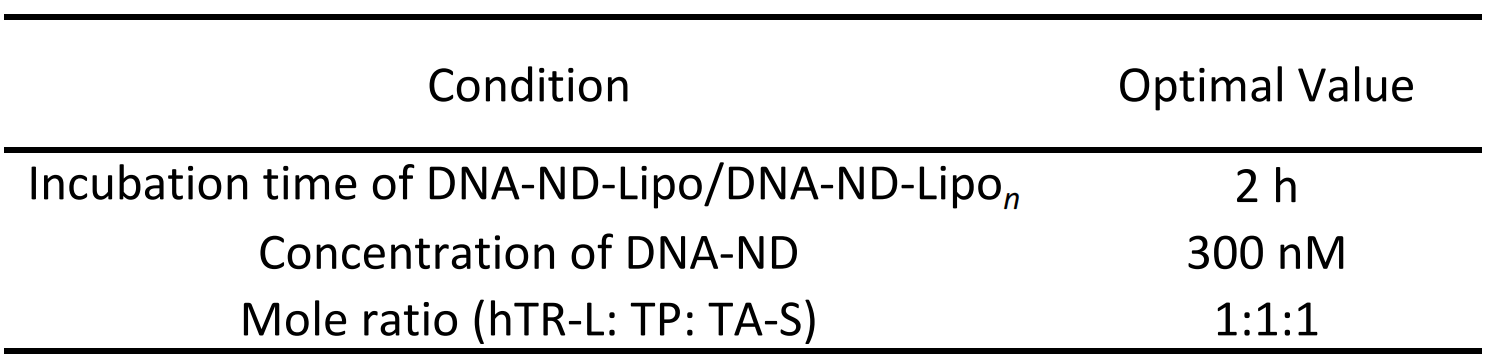


**Table S5.** XP&MM-GBSA results.


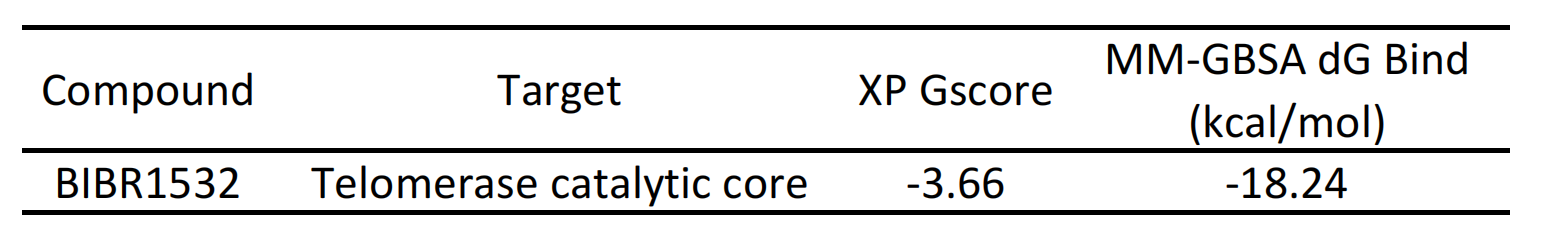


Comprehensive analysis of XP docking and MM-GBSA results showed that the docking score of BIBR1532 with TERT was -3.66, and the MM-GBSA result was -18.24 kcal/mol, with lower docking scores and free energies of binding, which indicated that the binding of BIBR1532 with TERT was more stable.

**Table S6.** BIBR1532 changes in binding energy with TERT.


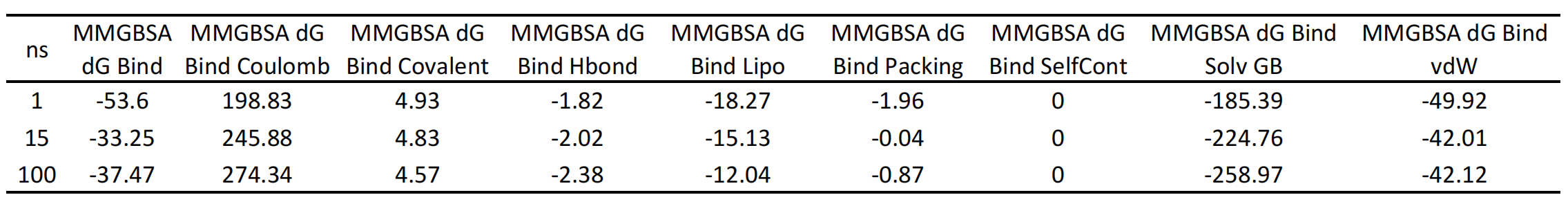


MMGBSA dG Bind = MMGBSA dG Bind (Coulomb + Covalent + Hbond + Lipo + Packing + SelfCont + Solv GB + vdW).

It can be found that the binding energy reaches the lowest state at 1 ns, but as time changes, by 15 ns the protein undergoes metastasis and the binding energy decreases to -33.25 kcal/mol, and then subsequently, as time changes, it reaches the steady state and the binding energy decreases to -37.47 kcal/mol.

**Table S7.** Model of TERT used in molecular docking and MD simulation.


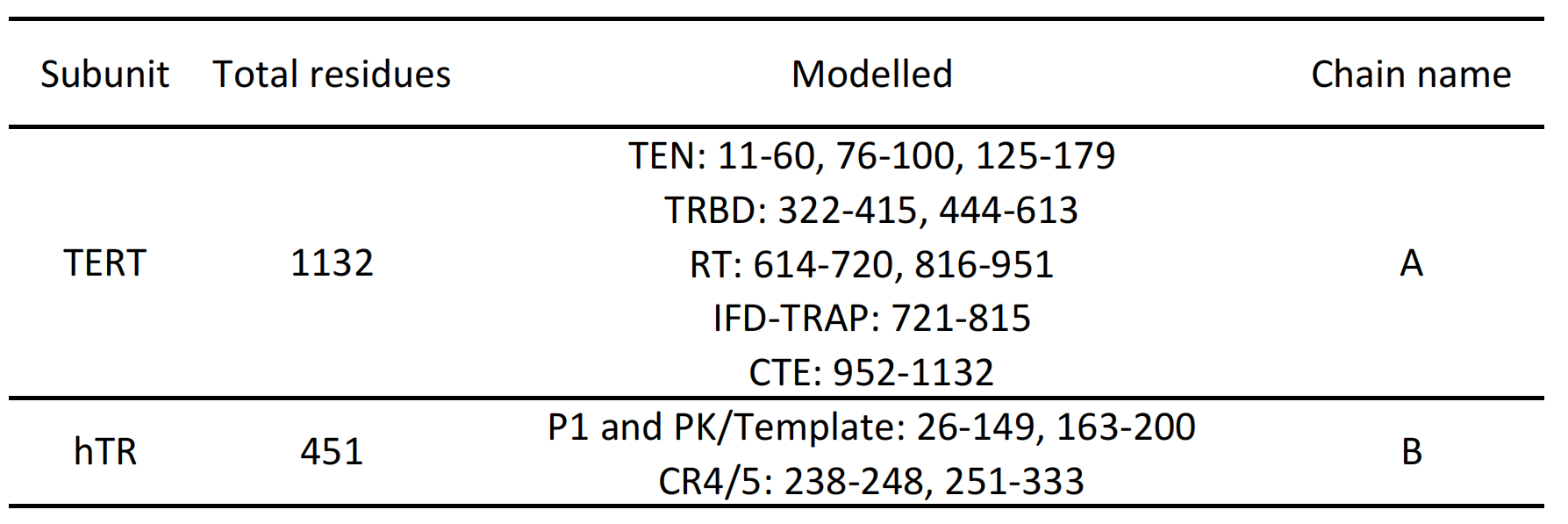


The modelled components for the catalytic core used in molecular docking are referred from Liu’s experiment[^1^](#_ENREF_1). Modifications were made from the original form to suit the needs of this study.

**MATLAB code used in logic analysis**

image1 = imread('AF 488.tif');

[height, width, ~] = size(image1);

logicMatrix1 = zeros(height, width);

for row = 1:height

for col = 1:width

red = image1(row, col, 1);

green = image1(row, col, 2);

blue = image1(row, col, 3);

if red > 40.| green > 40.| blue > 40

logicMatrix1(row, col) = 1;

else

logicMatrix1(row, col) = 0;

end

end

end

grayImage1 = mat2gray(logicMatrix1);

imshow(grayImage1);

colormap(gray);

title('Grayscale Logic Matrix for AF 488');

image2 = imread('Cy5.tif');

[height, width, ~] = size(image2);

logicMatrix2 = zeros(height, width);

for row = 1:height

for col = 1:width

red = image2(row, col, 1);

green = image2(row, col, 2);

blue = image2(row, col, 3);

if red > 40.| green > 40.| blue > 40

logicMatrix2(row, col) = 1;

else

logicMatrix2(row, col) = 0;

end

end

end

grayImage2 = mat2gray(logicMatrix2);

imshow(grayImage2);

colormap(gray);

title('Grayscale Logic Matrix for Cy5');

image3 = imread(Hochest33342.tif');

[height, width, ~] = size(image3);

logicMatrix3 = zeros(height, width);

for row = 1:height

for col = 1:width

red = image3(row, col, 1);

green = image3(row, col, 2);

blue = image3(row, col, 3);

if red > 40.| green > 40.| blue > 40

logicMatrix3(row, col) = 1;

else

logicMatrix3(row, col) = 0;

end

end

end

grayImage3 = mat2gray(logicMatrix3);

imshow(grayImage3);

colormap(gray);

title('Grayscale Logic Matrix for Hochest33342);

newLogicMatrix1 = zeros(height, width);

newLogicMatrix2 = zeros(height, width);

newLogicMatrix3 = zeros(height, width);

newLogicMatrix4 = zeros(height, width);

for row = 1:height

for col = 1:width

value1 = logicMatrix1(row, col);

value2 = logicMatrix2(row, col);

value3 = logicMatrix3(row, col);

if value3 == 1

if value1 == 1 && value2 == 1

newLogicMatrix1(row, col) = 1;

else

newLogicMatrix1(row, col) = 0;

end

end

end

end

grayImage1 = mat2gray(newLogicMatrix1);

figure;

imshow(grayImage1);

colormap(gray);

title('RG colocalized in nucleus');

for row = 1:height

for col = 1:width

value1 = logicMatrix1(row, col);

value2 = logicMatrix2(row, col);

value3 = logicMatrix3(row, col);

if value3 == 0

if value1 == 1 && value2 == 1

newLogicMatrix2(row, col) = 1;

else

newLogicMatrix2(row, col) = 0;

end

end

end

end

grayImage2 = mat2gray(newLogicMatrix2);

figure;

imshow(grayImage2);

colormap(gray);

title('RG colocalized in cytoplasm');

for row = 1:height

for col = 1:width

value1 = logicMatrix1(row, col);

value2 = logicMatrix2(row, col);

value3 = logicMatrix3(row, col);

if value3 == 1

if value1 == 1 && value2 == 1

newLogicMatrix3(row, col) = 0;

elseif value1 == 1.| value2 == 1

newLogicMatrix3(row, col) = 1;

end

end

end

end

grayImage3 = mat2gray(newLogicMatrix3);

figure;

imshow(grayImage3);

colormap(gray);

title('RG non- colocalized in cytoplasm');

for row = 1:height

for col = 1:width

value1 = logicMatrix1(row, col);

value2 = logicMatrix2(row, col);

value3 = logicMatrix3(row, col);

if value3 == 0

if value1 == 1 && value2 == 1

newLogicMatrix4(row, col) = 0;

elseif value1 == 1.| value2 == 1

newLogicMatrix4(row, col) = 1;

end

end

end

end

grayImage4 = mat2gray(newLogicMatrix4);

figure;

imshow(grayImage4);

colormap(gray);

title('RG non-colocalized in nucleus');

**Supplementary Reference**

[1] Roake, C. M. & Artandi, S. E. *Nat. Rev. Mol. Cell Biol.* **21**, 384-397 (2020). DOI:10.1038/s41580-020-0234-z.
